# Genome-wide identification of Reverse Transcriptase domains of recently inserted endogenous plant pararetrovirus (*Caulimoviridae*)

**DOI:** 10.3389/fpls.2022.1011565

**Published:** 2022-12-14

**Authors:** Carlos de Tomás, Carlos M. Vicient

**Affiliations:** Structure and Evolution of Plant Genomes Group, Centre for Research in Agricultural Genomics, CSIC-IRTA-UAB-UB, Edifici CRAG, Bellaterra, Barcelona, Spain

**Keywords:** pararetrovirus, Reverse Transcriptase (RT), *Caulimoviridae*, endogenous, virus

## Abstract

Endogenous viral elements (EVEs) are viral sequences that have been integrated into the nuclear chromosomes. Endogenous pararetrovirus (EPRV) are a class of EVEs derived from DNA viruses of the family *Caulimoviridae*. Previous works based on a limited number of genome assemblies demonstrated that EPRVs are abundant in plants and are present in several species. The availability of genome sequences has been immensely increased in the recent years and we took advantage of these resources to have a more extensive view of the presence of EPRVs in plant genomes. We analyzed 278 genome assemblies corresponding to 267 species (254 from *Viridiplantae*) using tBLASTn against a collection of conserved domains of the Reverse Transcriptases (RT) of *Caulimoviridae*. We concentrated our search on complete and well-conserved RT domains with an uninterrupted ORF comprising the genetic information for at least 300 amino acids. We obtained 11.527 sequences from the genomes of 202 species spanning the whole Tracheophyta clade. These elements were grouped in 57 clusters and classified in 13 genera, including a newly proposed genus we called *Wendovirus*. Wendoviruses are characterized by the presence of four open reading frames and two of them encode for aspartic proteinases. Comparing plant genomes, we observed important differences between the plant families and genera in the number and type of EPRVs found. In general, florendoviruses are the most abundant and widely distributed EPRVs. The presence of multiple identical RT domain sequences in some of the genomes suggests their recent amplification.

## Introduction

Endogenous viral elements (EVEs) are viral sequences that have been integrated into the nuclear chromosomes, enabling their vertical transmission and potential fixation in host populations (Feschotte and Gilbert, 2012). Viral integration within eukaryotic genomes is a widely recognized phenomenon described in many species thanks to the sequencing of whole genomes. Some of these EVEs are the consequence of a mandatory genome integration stage in the life cycle of reverse-transcribing viruses, such as retroviruses (Katzourakis and Gifford, 2010), but for others, such as all plant viruses, hepadnaviruses or the SARS-CoV-2, the integration in the host genome is not part of the virus life cycle and the mechanisms of integration are few well understood (Kojima et al., 2021; Zhang et al., 2021).

The first described plant EVE was a *Geminiviridae* element (Bejarano et al., 1996). EVEs derived from *Caulimoviridae* are abundant in plants (Diop et al., 2018). EVEs derived from another non-retroviral dsRNA, ssRNA, or ssDNA viruses have also been described as, for example, from *Narnaviridae* (Choi et al., 2021), *Partitiviridae, Betarhabdovirinae and Betaflexiviridae* (Chiba et al., 2011). The genome integration mechanism of the EVEs remains largely uncharacterized and different mechanisms for the integration were proposed. The most accepted theory is that endogenization results from a non-homologous recombination between virus and host genomes, usually in the context of either a double-stranded DNA break repair or a transposon-mediated process (Richert-Pöggeler et al., 2021).

If EVEs are integrated into or near host genes, this will be generally detrimental, and they will be removed from host population by purifying selection. In the rare cases that the integration of an EVE is beneficial, it will be fixed in the host population by positive selection, in the same way that occurs with other types of genomic elements like transposons (Catlin and Josephs, 2022). However, most of the EVEs are neutral and will become degraded due to the accumulation of disruptive mutations, insertions or deletions. Due to the random nature of these mutations, it is possible to reconstruct the sequences of the infectious viruses based on the EVEs sequences, particularly for high copy number EVEs (Aiewsakun and Katzourakis, 2015). In consequence, EVEs can be considered as genomic “fossils” and be employed for investigating viral origins and diversity and become the main tool for a new emerging field called Paleovirology. Paleovirology is the study of the ancient evolution of viruses through analyzing endogenous viral elements in the host genomes (Etienne, 2017). Due to the increasing number of sequenced genomes, numerous EVEs can be uncovered, and some of them are distinct from the currently known episomal viruses (Johnson, 2019). Another important property of EVEs is that they can be used to calibrate the timing of virus evolution. If an EVE is orthologous across several species, this gives a minimum estimate for the age of the virus that integrated into the genome (Aiewsakun and Katzourakis, 2015).

*Caulimoviridae* is a family of double-stranded DNA (dsDNA) viruses infecting plants that contain a reverse transcription stage in their replication cycle (International Committee on Taxonomy of Viruses, ICTV, https://ictv.global/). Although integration into the genome is not an essential part of their replication cycle, there are much evidence of their presence as integrated forms among genomes of the plant kingdom (Geering et al., 2014; Diop et al., 2018) and they have been included as a new category in some repetitive DNA sequence databases like Repbase (Bao et al., 2015). *Caulimoviridae* can be classified into 11 genera based on their genome organization (number of open reading frames and the arrangement of protein domains within them) and the morphology of their virus particles (ICTV, https://ictv.global/). Some of these genera have been reported as EVEs in plant genomes (Endogenous pararetrovirus, EPRVs) but, in addition, many of the EPRVs belong to a genus for which so far no episomal counterparts have been described (Geering et al., 2014; Chen and Kishima, 2016; Diop et al., 2018).

The integration of an EVE into or near a gene can potentially modify gene transcription or modify mRNA processing, resulting in mutant phenotypes. Most of the described EPRVs are inserted in intergenic regions and have no apparent deleterious effect on the host. However, there are examples of EPRVs inserted inside genes with potential effects on gene expression as, for example, in the case of *Vitis vinifera*, which has several EPRVs inserted in introns (Geering et al., 2014).

Most of the EPRVs are transcriptionally or translationally inactive because they are partial and/or comprise rearranged sequences and/or inactivating mutations. Often EPRVs form clusters resulting from the simultaneous integration of several complete or partial copies in tandem or nested (Richert-Pöggeler et al., 2003). Infrequently, these integrated sequences are transcriptionally active and the resulting RNAs can serve as precursors of extrachromosomal viral DNA and lead to systemic and vertically transmitted infections (Hohn et al., 2008; Gayral et al., 2008). Transcriptional activation can be driven by viral promoters present within the integrated element or plant promoters in the vicinity of the EPRV sequence (Lockhart et al., 2000; Kuriyama et al., 2020). On the other hand, EPRV derived RNAs can also be inducers for RNA interference (RNAi) and gene silencing mechanisms through the generation of small interfering RNAs (siRNAs) (Bertsch et al., 2009; Ricciuti et al., 2021).

RNA-directed DNA polymerase (Reverse Transcriptase, RT) coding sequences are present in a wide variety of genetic elements and contains a relatively well conserved central domain, allowing its use for phylogenetic analyses (Hansen and Heslop-Harrison, 2004) and for searches for homologues of, for example, EPRVs in genome sequences (Diop et al., 2018).

Previous studies have examined the EPRVs diversity in plant genomes based on the limited number of genome sequences available in each case (Geering et al., 2014; Diop et al., 2018) Nowadays, the number of sequenced plant genomes have increased significantly, and we decided to screen them for the presence of EPRVs, obtaining a broader picture of the distribution of these endogenous elements. We identified the major EPRV lineages and analyzed their distribution in the different plant orders and genera. We also describe a new possible genus of *Caulimoviridae* present only as EPRVs we called *Wendovirus*.

## Materials and methods

### Discovery and analyses of recently inserted endogenous *Caulimoviridae*

We built a library containing an assortment of 182 RT central domain amino acid sequences (**Supplementary Data 1**). This collection includes one sequence from *Retroviridae*, 14 from Ty3/Gypsy LTR retrotransposons of the six most abundant genera in plants (Athila, CRM, Galadriel, Ogre, Reina, Retand and Tekay), 104 from the eleven genera of *Caulimoviridae* (*Badnavirus*, *Caulimovirus*, *Vaccinivirus*, *Soymovirus*, *Cavemovirus*, *Solendovirus*, *Dioscovirus*, *Rosadnavirus*, *Tungrovirus*, *Petuvirus* and *Ruflodivirus*), and 63 from six groups of exclusively endogenous *Caulimoviridae* (*Florendovirus*, *Xendovirus*, *Yendovirus*, *Zendovirus*, *Gymnendovirus* and *Fernendovirus*) (hereafter referred to as operational taxonomic units (OTUs) following the nomenclature proposed by Diop et al., 2018. For further analyses, we selected ten sequences representatives of the *Caulimoviridae* groups (**Supplementary Data 2**).

We selected 278 genome assemblies corresponding to 267 species (**Supplementary Data 3**): two from *Bacteria*, one from *Chromista*, two from *Protozoa*, 13 from *Animal*, six from *Fungi* and 254 from *Plantae* kingdom. *Plantae* kingdom’s genomes include three *Rodophyta*, seven *Chlorophyta*, three *Bryophyta*, one *Marchantiophyta* and 240 *Tracheophyta* genomes. *Tracheophyta* includes one *Lycopodiopsida*, four *Pinopsida*, 35 *Liliopsida* (11 families) and 200 *Magnoliopsida* (46 families) genomes. The genomes outside the *Plantae* kingdom were used as negative controls.

We compared the ten RT sequences with the 278 genome assemblies using tBLASTn with default parameters (except –e option set to 1e−10). Only the hits with at least 300 amino acid residues and no stop codons nor frameshifts were selected for further analysis. To avoid the inclusion in the selection of tandem duplications, we removed a hit if it was located less than 1500 bp to another (**Supplementary Data 3**). For each genome assembly, the selected set of RT sequences were clustered with the 182 RT selected reference domains and those having higher similarity with retrotransposons were removed from the analyses. RT sequences having higher similarity with *Caulimoviridae* were used for further analyses (**Supplementary Data 4**).

For cluster determination, the selected sequences from the genome assemblies were grouped using CD-HIT with a sequence identity cut-off of 60% (Cluster60) or of 100% (Cluster100), a bandwidth of alignment of 20 and a length of sequence to skip of 10. One sequence was then selected to be representative of each cluster60 (**Supplementary Data 5**). Only in the case of cluster60-8 we selected two sequences because the sequences in this cluster were clearly divided in two groups.

The cluster representative sequences were aligned with the representative sequences of episomal or endogenous *Caulimoviridae* (**Supplementary Data 1**) using MEGA-X (Kumar et al., 2018). The resulting alignment was then used to build a phylogenetic reconstruction using the maximum likelihood (ML) method and 500 bootstrap replicates using MEGA-X. The resulting tree was then used as a reference to classify the EPRV-RTs found in the genome assemblies.

The minimum ages of the integration events reported in this study were inferred by identifying the most distantly related pair of host species sharing a particular cluster of EPRVs and applying the estimated species divergence dates in TimeTree (http://www.timetree.org/) (Kumar et al., 2017).

Potential ORFs were predicted using ORF Finder (https://www.ncbi.nlm.nih.gov/orffinder/) and the presence of Pfam domains in their encoded polypeptides was confirmed using MOTIF Search (https://www.genome.jp/tools/motif/).

## Results

### Distribution of genomic sequences encoding Reverse Transcriptase domains of recently inserted endogenous pararetroviruses (*Caulimoviridae*)

The objective of the work was to determine the presence of sequences encoding complete conserved RT domains corresponding to endogenous pararetrovirus (*Caulimoviridae*) within a collection of publicly available genome sequence assemblies from plant species and using some non-plant genome assemblies as negative controls. To identify them, we used a custom designed tBLASTn-based discovery pipeline, using as a probe a collection of 10 representative RT sequences of the different *Caulimoviridae* genera and OTUs (**Supplementary Data 2**). To give priority to the recently inserted copies, we only select sequences encoding RT domains of at least 300 amino acids that contain uninterrupted reading frames. Frequently EPRVs are inserted in tandemly arranged structures. To remove these duplications, when a RT coding region was located less than 1500 bp of another we only kept one of them. Due to their high sequence similarity, this first selection also contained RT sequences from Ty3/gypsy LTR-retrotansposons (*Metaviridae)*. To remove them, EPRVs were confirmed by phylogenetic analyses. They were aligned with RT sequences of representative *Caulimoviridae* and LTR retrotransposons (**Supplementary Data 1**). Those sequences showing higher similarity with the *Metaviridae* than with *Caulimoviridae* were removed. Finally, we obtained 11.527 RT-EPRV sequences (**Supplementary Data 4**).

None of the analyzed genomes outside *Plantae* Kingdom contain RT- EPRV sequences and among the genomes of the *Plantae* kingdom, we did not find RT-EPRVs in *Chlorophyta*, *Rodophyta, Bryophyta* or *Marchantiophyta*. Among the *Tracheophyta* species, we did not find RT-EPRVs in the class *Lycopodiopsida* (*Selaginella moellendorffii*) but we found RT-EPRVs in genomes of all *Tracheophyta* classes (*Pinopsida*, *Liliopsida* and *Magnoliopsida*), confirming previous results (Gong and Han, 2018). All the four *Pinopsida* genomes analyzed contain RT-EPRV sequences (between 4 and 46). We included 35 genomes of species of the class *Liliopsida* and we found RT-EPRV sequences in 22 of them (63%) (between 1 and 63). Finally, we found RT-EPRV sequences in 180 of the 201 *Magnaliopsida* genomes (88%) (between 1 and 1186).

When comparing the results with the genomes of species belonging to the same genus, or varieties of the same species, the results obtained are, in general, similar. For example, the genomes of the two species of *Kalanchoe* contain 20 and 24, the two of *Vitis* contain 24 and 29 and the three of *Solanum* between 29 and 35. However, this is not always the case, and we can observe important differences in the number of RT-EPRVs in species of the same genus. For example, in the genera *Arachis* (between 56 and 473), *Prunus* (between 3 and 144), *Rosa* (between 76 and 340), *Citrus* (between 63 and 306) and *Nicotiana* (between 12 and 130). Some of these differences can be due to differences in the quality of the genome assemblies. For example, the presence of undetermined nucleotides can give rise to a reduction in the number of RT-EPRVs we detected. However, there are cases in which the best quality genome is the one with the least number of sequences. For example, we included three species of the genera *Arabidopsis* and the genome with the least number of sequences is the one with the best quality *(Arabidopsis thaliana*). All these results suggest that in some of the species there have been very recent integrations of EPRVs.

### Classification of the RT-EPRVs present in plant genomes

**Figure 1:**
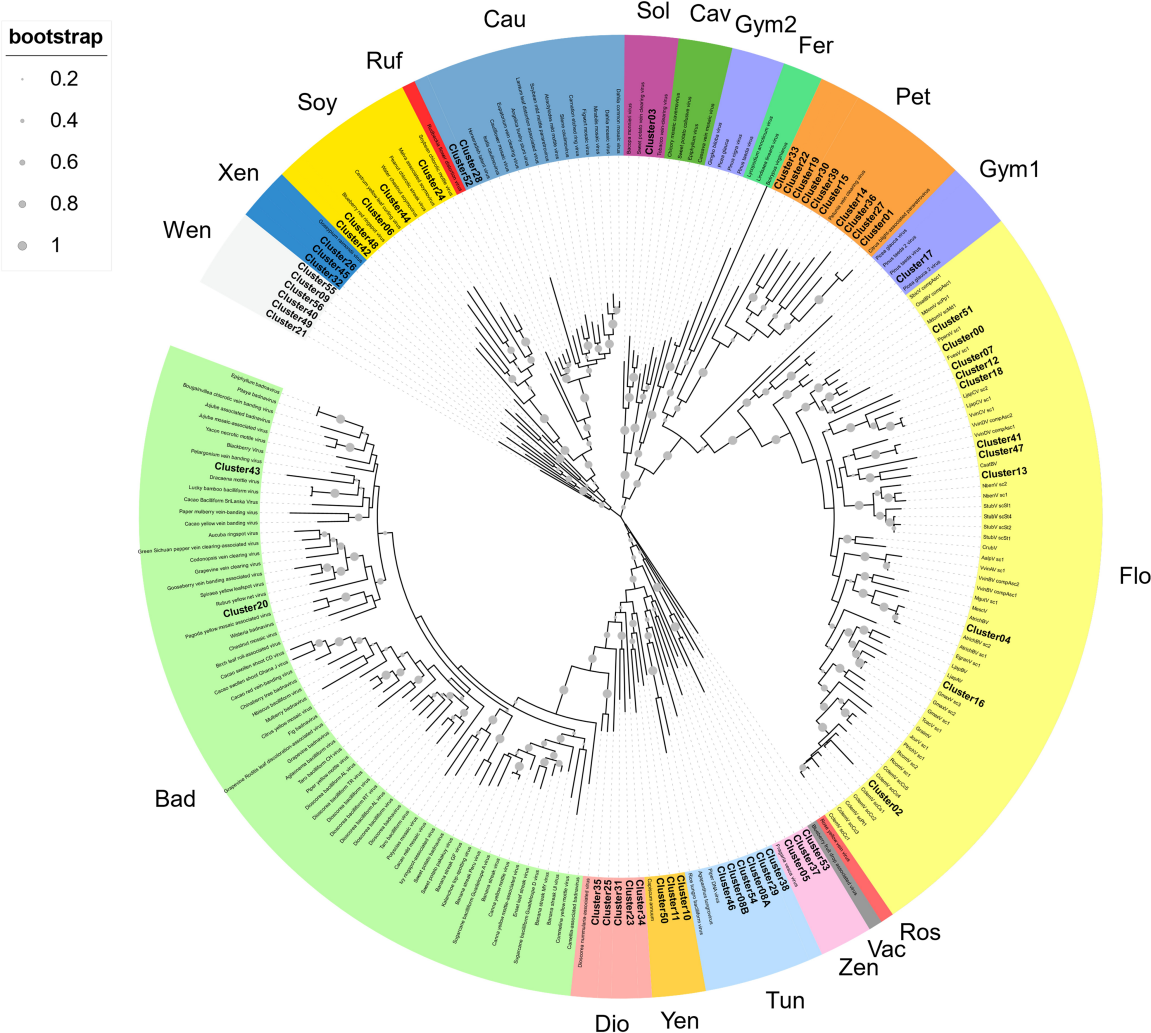
Phylogenetic relationships within the episomal and endogenous *Caulimoviridae*. Phylogram obtained from a maximum likelihood analysis with protein sequence data from RT conserved domains using 500 bootstrap replications. The size of the point indicated the bootstrap support of the tree branch. Known episomal and endogenous pararetrovirus are shown in grey and small letters. New endogenous Clusters60 are shown in bold letters. The color of the branch indicates the genus of *Caulimoviridae*; Bad, *Badnavirus*; Dio, *Dioscovirus*; Yen, *yendovirus*; Tun, *tungrovirus*; Zen, *zendovirus*; Vac, *vaccinivirus*; Ros, *rosadnavirus*; Flo, *florendovirus*; Gym1 and Gym2, *gymnendovirus*1 and 2; Pet, *petuvirus*; Fer, *fernendovirus*; Cav, *cavemovirus*; Sol, *solendovirus*; Cau, *caulimovirus*; Ruf, *ruflodivirus*; Soy, *soymovirus*; Xen, *xendovirus*; and Wen, *wendovirus*.

To provide a classification, RT-EPRV sequences with at least 60% amino acid identity to each other were grouped, yielding a total of 57 clusters. The total number of sequences and genomes represented in each cluster varies greatly (**Table 1**). We performed a phylogenetic analysis using representative sequences of each cluster (**Supplementary Data 5**) and representatives of all *Caulimoviridae* genera and OTUs (**Supplementary Data 1**). Our phylogenetic analysis clustered together all the previous known sequences corresponding to the same genera and OTU of the *Caulimoviridae*, confirming the robustness of the analysis (**Figure 1**). This phylogenetic reconstruction allowed us to determine the diversity and nature of our collection of RT-EPRV sequences (**Table 2**). They were separated into 13 *phyla*. 30 of the clusters were associated with sequences of *Caulimoviridae* with episomal forms: 10 *Petuvirus*, 5 *Dioscovirus*, 5 *Soymovirus*, 5 *Tungrovirus*, 2 *Badnavirus*, 2 *Caulimovirus* and 1 *Solendovirus*. We did not find any representative of the genera *Cavemovirus*, *Rosadnavirus* or *Vaccinivirus*, and neither from the recently proposed genera *Ruflodivirus*. This result suggests that the virus species of these genera do not carry out endogenization, at least not recently or as frequently, or they only do it in a small range of species whose complete genomic sequence is not yet available. Of the rest, 20 clusters corresponded to OTUs from which only endogenous forms have been found: 11 *Florendovirus*, 3 *Xendovirus*, 3 *Yendovirus*, 3 *Zendovirus* and 1 *Gymnendovirus*. As we will describe later in detail, the remaining 6 clusters were associated with each other, forming a new OTU we called *Wendovirus* (**Figure 1**).

**Table 1:** Cluster60 statistics.

Cluster	Cluster N.	EPRV-RT-seqs	N.Classes	N.Orders	N.Families	N.Genus	N.Species	A	B	Max.Age (MY)
**BADNAVIRUS**		80	3	10	11	12	13		
Badnavirus-01	20	75	2	9	10	12	12	Dioscorea	Amborella	191
Badnavirus-02	43	5	1	2	2	2	2	Phalaenopsis	Musa	117
**CAULIMOVIRUS**		38	1	4	4	6	9			
Caulimovirus-01	28	36	1	3	3	5	8	Helianthus	Arabidopsis	118
Caulimovirus-02	52	2	1	1	1	1	1	Gossypium	Gossypium	0
**DIOSCOVIRUS**		144	2	5	5	7	9			
Dioscovirus-01	23	49	1	3	3	4	5	Cynara	Cajanus	118
Dioscovirus-02	25	43	1	1	1	1	2	Dioscorea	Dioscorea	0
Dioscovirus-03	31	24	1	1	1	2	2	Glycine	Vigna	23
Dioscovirus-04	34	16	1	1	1	1	1	Macadamia	Macadamia	0
Dioscovirus-05	35	12	1	1	1	1	2	Dioscorea	Dioscorea	0
**PETUVIRUS**		1693	2	14	16	47	66			
Petuvirus-01	1	1202	1	5	5	10	19	Arachis	Citrus	108
Petuvirus-02	14	131	1	9	9	16	18	Amborella	Helianthus	191
Petuvirus-03	15	129	1	3	4	6	9	Coffea	Gossypium	118
Petuvirus-04	19	78	1	1	1	11	13	Brassica	Rorippa	27
Petuvirus-05	22	52	1	1	1	1	1	Ipomoea	Ipomoea	0
Petuvirus-06	27	39	1	1	1	7	9	Arachis	Cicer	59
Petuvirus-07	30	24	1	3	3	4	6	Populus	Gossypium	108
Petuvirus-08	33	18	1	1	1	3	8	Citrus	Atalantia	18
Petuvirus-09	36	12	1	2	2	2	2	Durio	Macadamia	123
Petuvirus-10	39	8	1	1	1	1	1	Eucalyptus	Eucalyptus	0
**SOLENDOVIRUS**		1124	1	2	2	5	8			
Solendovirus-01	3	1124	1	2	2	5	8	Nymphaea	Nicotiana	179
**SOYMOVIRUS**		454	1	5	6	12	14			
Soymovirus-01	6	391	1	1	1	1	3	Arachis	Arachis	0
Soymovirus-02	24	49	1	4	5	6	6	Lactuca	Cleome	118
Soymovirus-03	42	6	1	1	1	1	1	Chenopodium	Chenopodium	0
Soymovirus-04	44	5	1	1	1	3	3	Brassica	Cakile	13
Soymovirus-05	48	3	1	1	1	1	1	Medicago	Medicago	0
**TUNGROVIRUS**		308	2	5	5	10	32			
Tungrovirus-01	8	251	1	3	3	10	29	Prunus	Vitis	117
Tungrovirus-02	29	32	1	1	1	1	1	Lindenbergia	Lindenbergia	0
Tungrovirus-03	38	9	1	1	1	1	1	Cinnamomum	Cinnamomum	0
Tungrovirus-04	46	4	1	1	1	1	2	Malus	Malus	0
Tungrovirus-05	54	2	1	1	1	1	1	Citrus	Citrus	0
**FLORENDOVIRUS**		6162	3	29	40	91	151			
Florendovirus-01	0	3207	2	27	34	70	114	Asparagus	Amborella	191
Florendovirus-02	2	1188	1	6	8	21	35	Brassica	Nicotiana	118
Florendovirus-03	4	949	2	21	27	38	47	Asparagus	Amborella	191
Florendovirus-04	7	317	1	2	2	2	3	Coffea	Lindenbergia	77
Florendovirus-05	12	133	1	1	1	3	5	Arachis	Lotus	59
Florendovirus-06	13	132	1	2	2	5	8	Lindenbergia	Nicotiana	79
Florendovirus-07	16	120	1	8	9	13	18	Amborella	Brassica	191
Florendovirus-08	18	79	1	2	2	7	8	Glycine	Manihot	101
Florendovirus-09	41	7	1	1	2	2	2	Capsicum	Nicotiana	24
Florendovirus-10	47	4	1	1	1	2	2	Cucumis	Momordica	48
Florendovirus-11	51	2	2	2	2	2	2	Asparagus	Prunus	160
**GYMNENDOVIRUS**		95	1	1	1	2	3			
Gymnendovirus-1-1	17	95	1	1	1	2	2	Pinus	Picea	130
**WENDOVIRUS**		282	1	7	7	10	17			
Wendovirus-01	9	200	1	1	1	4	11	Citrus	Atalantia	18
Wendovirus-02	21	70	1	2	2	3	3	Helianthus	Coffea	101
Wendovirus-03	40	7	1	2	2	3	4	Citrus	Solanum	118
Wendovirus-04	49	3	1	1	1	1	1	Lindenbergia	Lindenbergia	0
Wendovirus-05	55	1	1	1	1	1	1	Olea	Olea	0
Wendovirus-06	56	1	1	1	1	1	1	Portulaca	Portulaca	0
**XENDOVIRUS**		65	1	6	6	8	10			
Xendovirus-01	26	41	1	4	4	6	8	Vaccinium	Rosa	118
Xendovirus-02	32	19	1	1	1	1	1	Olea	Olea	0
Xendovirus-03	45	5	1	1	1	1	1	Ipomoea	Ipomoea	0
**YENDOVIRUS**		334	2	6	7	17	23			
Yendovirus-01	10	190	1	1	1	9	11	Oryza	Eleusine	47
Yendovirus-02	11	142	2	5	5	8	12	Dioscorea	Solanum	160
Yendovirus-03	50	3	2	2	2	2	2	Ananas	Nymphaea	179
**ZENDOVIRUS**		781	1	2	2	5	19			
Zendovirus-01	5	768	1	1	1	4	18	Fragaria	Rubus	41
Zendovirus-02	37	11	1	1	1	2	4	Fragaria	Rosa	31
Zendovirus-03	53	2	1	1	1	1	1	Pistacia	Pistacia	0

**Table 2:** Distribution of Cluster60 in plant families.

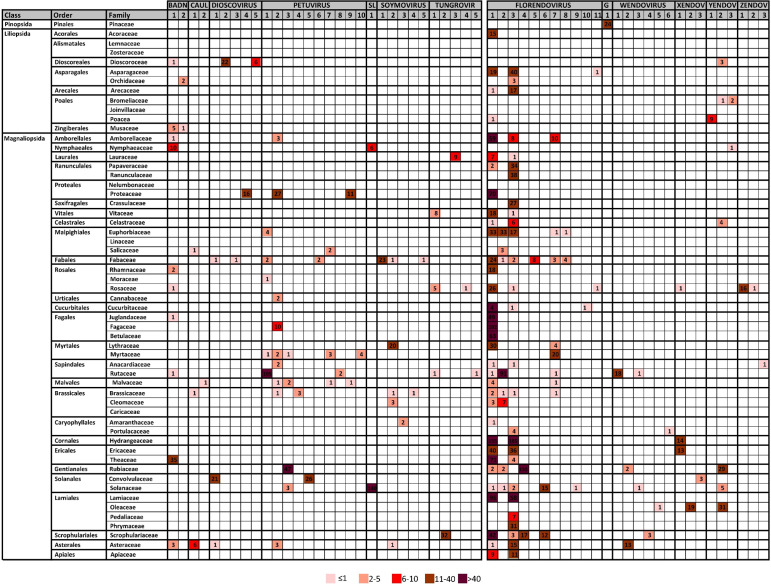

Numbers are the average number of RT-EPRV sequences per genome of each cluster 60. The genomes are grouped according to the plant families. BADN, *Badnavirus*; CAUL, Caulimovirus; SL, Solendovirus; G, Gymnendovirus.

We observed important differences between genera for both the number of RT- EPRV sequences and the diversity of species in which they were found (**Table 1**). *Florendovirus* are clearly the most abundant followed by *Petuvirus*, *Solendovirus* and *Zendovirus*. However, whereas *Florendovirus* is present in genomes of 40 families of species, *Petuvirus* is present in 14 and *Solendovirus* and *Zendovirus* in only two. Interestingly, although we only detected 80 RT-EPRV sequences corresponding *Badnavirus*, they present a wide distribution (3 Classes, 10 Orders and 11 Families). On the opposite, *Gymnendovirus* are only present in *Pinopsida*.

If we look at the different classes of plants, we observed important differences. *Pinopsida* only contains *Gymnendovirus*. *Magnolids* contains *Badnavirus*, *Petuvirus*, *Solendovirus*, *Tungrovirus*, *Florendovirus* and *Yendovirus*. *Liliopsida* contains *Badnavirus*, *Dioscovirus*, *Florendovirus* and *Yendovirus*. Finally, *Magnaliopsida* contains all the genera except *Gymnendovirus*.

If we look at the distribution of the clusters in the different plant species, we observed a wide diversity (**Table 2**). Some of them are exclusively present in one class. For example, Gymnendovirus-1 is only present in *Pinopsida*, Tungrovirus-3 is only present in *Magnolids*, Badnavirus-2, Dioscovirus-2 and -5 and Yendovirus-1 are only present in *Liliopsida*, and many clusters are only present in *Magnaliopsida*. On the opposite, Badnavirus-1, Florendovirus-1 and Florendovirus-3 are present in *Magnolids*, *Liliopsida* and *Magnoliopsida*. Looking at more detail, 31 of the 57 clusters are present in genomes of only one family of plants, whereas two are present in genomes of more than 20 plant families (both florendovirus). These differences of distribution are reflected in the Maximum Age Value (**Table 1**), which depends on the maximum phylogenetic distance between the species present in the cluster.

### Very recent EPRV amplification in plant genomes

The above results suggest that, at least in some species, there has been a recent amplification in the number of EPRV sequences inserted in their genomes. To try to delve further into this aspect, we decided to select those cases in which 100% identical RT-EPRV sequences were present in 10 or more copies in the same genome. Using this highly restrictive criterion, we detected 31 clusters grouping a total of 1534 sequences (**Table 3**). These clusters (clusters100) involve 19 genomes. Only one corresponds to a *Liliopsida (Hordeum vulgare*) and the remaining 18 are genomic sequences of *Magnaliophyta*. Nine EPRV OTUs are represented in the Clusters100 including *Caulimovirus*, *Dioscovirus*, *Florendovirus*, *Petuvirus*, *Solendovirus*, *Tungrovirus*, *Yendovirus*, *Zendovirus* and the newly proposed *Wendovirus*.

**Table 3:** Cluster 100 with 10 or more copies.

Cluster 100%	Num. Seq.	Genome	EPRV group
1	951	*Capsicum annuum*	Solendovirus-01
2	77	*Lotus japonicus*	Florendovirus-01
3	53	*Citrus maxima*	Petuvirus-01
4	43	*Hydrangea quercifolia*	Florendovirus-01
5	27	*Citrus medica*	Petuvirus-01
6	26	*Citrus medica*	Petuvirus-01
7	24	*Salvia splendens*	Florendovirus-03
8	22	*Ipomoea triloba*	Petuvirus-05
9	21	*Capsicum annuum*	Yendovirus-02
10	20	*Capsicum annuum*	Florendovirus-03
11	20	*Atalantia buxifolia*	Petuvirus-01
12	19	*Fortunella hindsii*	Florendovirus-02
13	19	*Atalantia buxifolia*	Petuvirus-01
14	16	*Helianthus annuus*	Wendovirus-02
15	16	*Ipomoea triloba*	Dioscovirus-01
16	14	*Fortunella hindsii*	Florendovirus-02
17	13	*Lactuca sativa*	Florendovirus-03
18	12	*Castanea dentata*	Florendovirus-01
19	12	*Atalantia buxifolia*	Florendovirus-02
20	12	*Nicotiana tabacum*	Solendovirus-01
21	12	*Lindenbergia philippensis*	Tungrovirus-02
22	11	*Lactuca sativa*	Caulimovirus-01
23	11	*Lotus japonicus*	Florendovirus-01
24	11	*Atalantia buxifolia*	Florendovirus-02
25	11	*Capsicum annuum*	Florendovirus-03
26	11	*Capsicum annuum*	Solendovirus-01
27	10	*Fragaria nilgerrensis*	Florendovirus-01
28	10	*Arachis hypogaea*	Florendovirus-01
29	10	*Nicotiana sylvestris*	Solendovirus-01
30	10	*Hordeum vulgare*	Yendovirus-01
31	10	*Rosa chinensis*	Zendovirus-01

Cluster100-10 is particularly noteworthy as it includes 951 sequences present in the genome of pepper (*Capsicum annuum*). Another four groups also correspond to the same genome, with a total of 1014 sequences (962 are *Solendovirus*, 31 are *Florendovirus* and 21 *Yendovirus*). In total, we found 1183 RT-EPRV sequences in this genome and more than 81% are present in the Cluster100 selection. This is a very clear indication of a relatively recent proliferation of EPRVs in the pepper genome.

**Figure 2:**
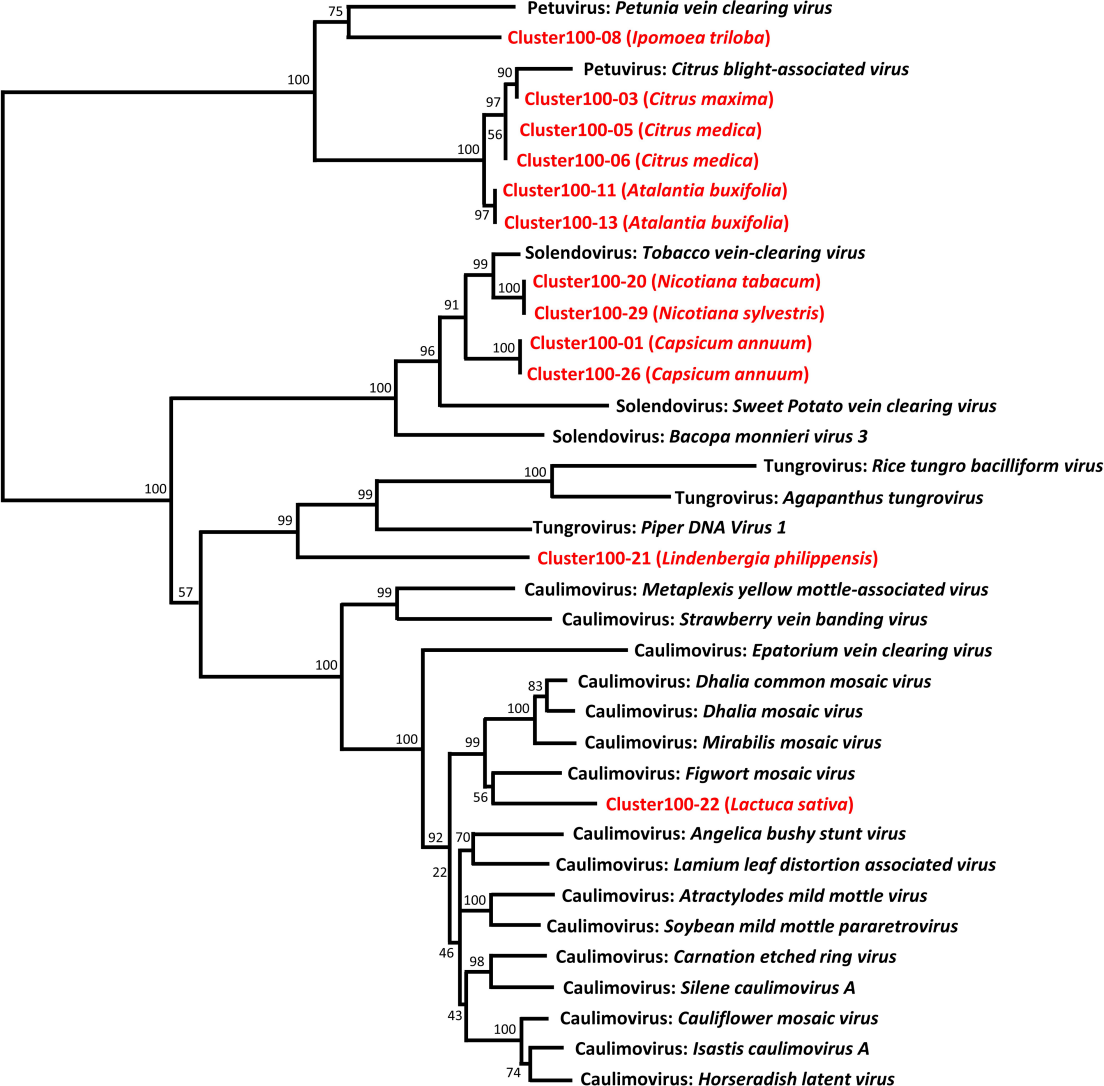
Phylogenetic relationships of representative sequences of the Cluster100. Representative sequences of the RT-EPRV Cluster100 (in red) were aligned with RT sequences of pararetroviral elements (in black), and a phylogenetic tree was constructed using the NJ method and 1000 bootstrap replications.

Next, we perform a phylogenetic analysis of representatives of each Cluster-100 and from the described OTUs from *Caulimoviridae* (**Figure 2**). The sequences of some of the clusters100 are very similar and, probably, they correspond to the same virus. This is the case of clusters100-1 and -26 (*Solendovirus* of *Capsicum annuum*), clusters100-11 and -13 (*Petuvirus* of *Atalantia buxifolia*) and Clusters100-5 and -6 (*Petuvirus* of *Citrus medica*). The sequences of clusters100-12 and -16 (*Florendovirus* of *Fortunella hindsii*) and of clusters100-19 and -24 (*Florendovirus* of *Atalantia buxifolia*) are also near identical. The sequences of the Clusters100-20 and 29, that correspond to two different but closely related species (*Nicotiana tabacum* and *Nicotiana sylvestris*), are also almost identical, which suggests that they could come from the same virus capable of infecting both species. **Figure 2** also shows that some of the endogenous sequences grouped in Clusters100 are very similar to the sequences of episomal virus. For example, the RT sequence of the citrus blight associated virus is highly similar to the sequences of cluster100-3, -5 and -6, all of them belonging to genomes of the genus *Citrus*, and the sequence of the tobacco vein clearing virus is similar to clusters100-20 and -29, belonging to genomes of the genus *Nicotiana*.

### Wendovirus, a new group of Caulimoviridae

Six of the Cluster60 and one of the Cluster100 correspond to a new group of endogenous *Caulimoviridae* with distinctive characteristics that, following the nomenclature proposed by Diop et al. (2018) (*Zendovirus*, *Xendovirus* and *Yendovirus*), we have called them *Wendovirus* (**Supplementary Data 4** and **Table 3**).

**Figure 3:**
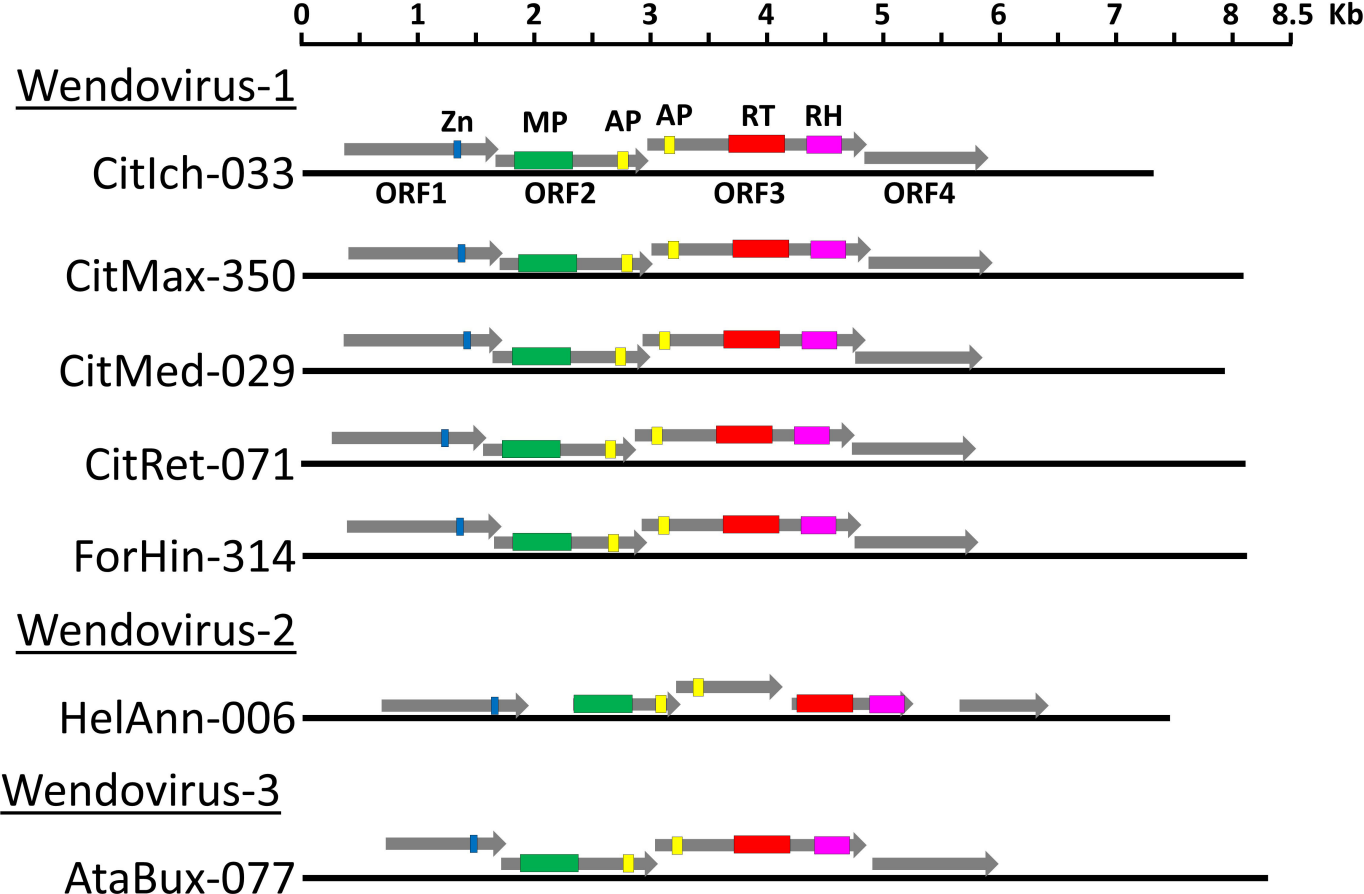
Schematic representation of *wendovirus* endogenous pararetrovirus. A scaled linear view of the genome organization of *Wendovirus*. The name of the sequences is the same as in **Supplementary Data 4**. Grey arrows mark open reading frames and colored regions within ORFs are conserved protein domains: blue, zinc finger typically present in the coat proteins; green, Movement Protein; yellow, Aspartic Proteinase; red, Reverse Transcriptase; pink, RNaseH.

We were able to reconstruct the structure of the *Wendovirus* for seven genomes corresponding to Cluster60 (**Figure 3**; **Supplementary Data 6**). The structure was very similar in all of them, with four partially overlapping ORFs. Comparisons with protein motif databases allowed us to find different conserved domains (**Supplementary Data 6**). The ORF1 encodes for a zinc finger motif, which is typical of the *Caulimoviridae* coat proteins. The ORF2 encodes for a movement protein and an aspartic proteinase. The ORF3 encodes a second aspartic proteinase, the RT and the RNAseH. Finally, the ORF4 encodes a protein without significant homologies to other reference proteins and without known protein domains but that is well-conserved in all the wendovirus elements. The most noticeable aspect of these structures is the presence of two aspartic proteinase domains instead of one, as usual. They are located close to each other, but in two different ORFs (2 and 3). In the case of the HelAnn-006 element (Wendovirus2 cluster), although the domains and their order are conserved, the ORF2 is shorter and the ORF3 is divided in two. When compared to databases, the highest similarities of these two aspartic proteinase domains are with members of *Caulimoviridae*.

## Discussion

Endogenous viral elements (EVEs) are viral sequences integrated in host genomes that are inherited as host DNA sequences (Holmes, 2011). Some of the EVEs, are derived from viruses in which integration into the genome is part of their replication cycle, for example, mammalian retroviruses. However, many viruses in which integration into the genomic DNA is not a part of their normal replication cycle can also be found as EVEs, as is the case of the endogenous *Caulimoviridae* (Endogenous Pararetrovirus, EPRVs). The presence of EPRVs has been described in the genomes of different plant species (Hohn et al., 2008). In this work we have focused on determining the presence of EPRV sequences relatively recently integrated, based on the selection of elements with complete and conserved RT domains.

Based on the RT domain sequence similarity we detected 11.527 sequences distributed in 57 clusters corresponding to 13 OTUs. Twelve of these groups had already been described (Diop et al., 2018) and one is shown here for first time, we called *Wendovirus*. Contrary to what has been observed in other plant viruses as *Geminivirus* or *Nanovirus* (Nino Barreat and Katzourakis, 2021), EVEs from *Caulimoviridae* are exclusively present in plants. Recently integrated RT-EPRVs are present in genomes of *Lycopodiopsida*, *Pinopsida*, *Liliopsida* and *Magnoliopsida*, but not necessary in all the genomes of these groups. For example, they are not present in the genomes of *Arabidopsis thaliana*, *Zea mays*, *Triticum aestivum*, *Phaseolus vulgaris*, *Theobroma cacao* or *Spinacia oleracea*. They are also absent in the *Selaginella moellendorffii* (*Marchantiophyta*) and in *Rhodophyta*, *Chlorophyta* or *Bryophyta*.

We have found that, in some cases, the integration events can be considered very recent. Once in the genome, the EPRV sequences begin to accumulate point random mutations, so, if the sequences are identical that means that they probably integrated recently in the genome. We have found multiple sequences encoding identical RT domains in different species being the most extreme case *Capsicum annuum* in whose genome we found up to 951 sequences encoding identical RT domains. Recent genome integrations of *Caulimoviridae* sequences have been described in some species, such as banana (Gayral et al., 2010). It is interesting to note that, in some cases, these identical RT sequences correspond to groups that have only been detected as endogenous forms (*Florendovirus*, *Yendovirus*, *Zendovirus*, *Wendovirus*) suggesting that probably at least some of them may have their corresponding episomal virus species that have not been yet identified.

The distribution of the different clusters of EPRVs between species shows a great diversity. Some clusters are present exclusively in certain plants as, for example, *Gymnendovirus* in *Pinopsida*, *Zendovirus*1 in the tribus *Potentilleae* and *Roseae*, *Soymovirus*1 in the genus *Arachis* or *Wendovirus*1, only present in *Rutaceae*. In other cases, such as *Florendovirus*1 and 3, the distribution is very wide, including *Lilipsida* and *Magnoliopsida*. In general, the distribution of the different groups of EPRVs is consistent with the phylogeny, but not always. For example, *Petuvirus*2 are present in *Amborella trichopoda* and in eight *Magnoliopsida* orders, *Florendovirus*7 are present in *Amborella trichopoda* and in seven *Magnoliopsida* orders and *Solendovirus*1 are present in *Nymphaea colorata* and in *Solanaceae*. A possible explanation for these species distributions is the horizontal transmission of the virus between species. There are data suggesting multiple viral jumps between different animal species in *Hepadnavirus* (Dill et al., 2016), and previous data also suggests such horizontal transfers can occur for EPRVs in plants (Diop et al., 2018; Gong and Han, 2018).

We have detected differences in the number of EPRVs in the different genomes. Sometimes the differences are also observed comparing the genomes of species of the same genus or varieties of the same species. The number of EPRVs observed results from the combination of the virus integration and the mechanisms of amplification or reduction of the integrated sequences. First, *Caulimoviridae* integration requires the presence of viruses that are infectious for the species and that the defense mechanisms of the plant are not able to eliminate, or not completely. Second, the main integration mechanism is thought to involve illegitimate recombination, which requires the existence of DNA double-strand breaks and subsequent repair mechanisms (Richert-Pöggeler et al., 2021). Furthermore, to be transmitted, integration must occur in reproductive cells. Third, once integrated, EPRVs, copies are inactivated by sequence degeneration or fragmentation, or by the insertion of transposable elements, and subjected to epigenetic silencing (reviewed by Richert-Pöggeler et al., 2021). All these processes lead to the degeneration of the coding sequences. Finally, it has also been proposed that once integrated, the sequences can be amplified, and different mechanisms have been suggested such as transposition like retroelements, rolling circle amplification, unequal meiotic crossing-over of tandem arrays, or ectopic recombination between EPRV clusters on non-homologous chromosomes (reviewed by Richert-Pöggeler et al., 2021). Variations in any of these processes together with the time elapsed since the last event of integration could explain the observed differences in the number of EPRVs in the analyzed genomes. Nor can we rule out that the different quality of the genome assemblies may also affect.

We have identified a new putative genus of the *Caulimoviridae*, tentatively named ‘*Wendovirus*’. *Wendovirus* genomes are about 7,7 Kb long and are present in the genomes of different *Magnaliopsida* species, especially in *Rutaceae* and in sunflower. Our phylogenetic analysis shows that *wendovirus* are related to *Xendovirus* and *Soymovirus*. They contain four ORFs that encode the typical protein domains in *Caulimoviridae*: Zinc-Finger, Movement Protein, Aspartic Proteinase, Reverse Transcriptase and RNAseH. A remarkable feature of *wendovirus* is the presence of two protease coding domains located in two different ORFs (**Figure 3**). Although both encode aspartyl proteases, the domains are different (PF13975 in ORF2 and PF00077 in ORF3), so the hypothesis that their origin was a genomic duplication can be discarded. When compared to protein bases, all these described domains, including the two aspartic proteinase domains, show the greatest similarities against other members of *Caulimoviridae*. Therefore, it seems to be ruled out that the second proteinase domain could come from some other families of viruses. Recombination between EPRV fragments has been observed (Chabannes and Iskra-Caruana, 2013) and many viruses have modularly acquired domains and ORFs (Smyshlyaev et al., 2013; Koonin et al., 2015). Encapsidation of genomes (or genome fragments) of different species of *Caulimoviriridae* in the same capsid can lead to recombination and formation of chimeric genomes. Virus-like particles (VLPs) containing host RNAs were found to be produced during agroinfiltration of cucumber necrosis virus, some of them corresponding to retrotransposon or retrotransposon-like RNA sequences (Ghoshal et al., 2015). On the other hand, template switching between two RNA molecules during reverse transcription has been shown for retroviruses, LTR retrotransposons and is proposed for *Caulimoviridae* (Froissart et al., 2005; Tromas et al., 2014; Sanchez et al., 2017; Richert-Pöggeler et al., 2021). Such an acquisition of ORFs likely contributed to the evolution of the *Wendovirus*, although the possible functions of this second proteinase domain remain unknown.

## Data availability statement

The original contributions presented in the study are included in the article/**Supplementary Material**. Further inquiries can be directed to the corresponding author.

## Author contributions

All authors contributed equally to the design and processing data. All authors have read and approved the final manuscript.

## Supplementary material

Supplementary Data Sheet 1 RT domain sequences of *Caulimoviridae*, retrovirus and LTR-retrotransposons used in this study.

Supplementary Data Sheet 2
*Caulimoviridae* RT sequences used in the tBLASTn analyses.

Supplementary Data Sheet 3 Genome assemblies used in the tBLASTn analyses.

Supplementary Data Sheet 4 RT-EPRV sequences found.

Supplementary Data Sheet 5 Representative RT-EPRV sequences for the clusters60 used in the phylogenetic analysis.

Supplementary Data Sheet 6
*Wendovirus* sequences and polypeptides encoded.
