# Supplementary material for: Genome-wide identification of Reverse Transcriptase domains of recently inserted endogenous plant pararetrovirus (Caulimoviridae)
Source: Front Plant Sci. 2022 Dec 14;13:1011565. doi: 10.3389/fpls.2022.1011565 (PMC9794742; doi:10.3389/fpls.2022.1011565)
Supplement: Supplementary Data Sheet 1 — RT domain sequences of Caulimoviridae, retrovirus and LTR-retrotransposons used in this study. [file DataSheet_1.docx]

>Badnavirus01; Agave badnavirus A_MH898467;

IDKLKKAGIIGNDPLKFWEKNQVKCKLEIINPNLIIEDRPLKHVTPAMRDQMRKHIKALLDLKVIRPSTSKHRTTAMLVNSGSDVISDQDAQGRKIQREVKGKERLVFNYKRLNDNTEKDQYSLPGINTIIQRIGHSKIYSKFDLKSGFHQVAMDPESIPWTAFWAIDGLYEWLVMPFGLKNAPAIFQRKMDQCFKGMEDFTAVYIDDILVFSETPEAHARHLTQVLQVCEKEGLILSPTKMKIGVSTIDFLGATIGDAKIKLQPHIIKKILEVRNDDLETTKGLRAWLGILNYARSYIPQLGRILGPLYAKVSPTGERRM

>Badnavirus02; Aglaonema bacilliform virus_NC_055236;

LDELKRAGYVGEDPLKFWASNKIMCHLDIKNPDLIIEDRPLKQISPTLEAAYKKHIDALLALHVIRPSTSRHRTAAIIVNSGISIDPVTQQEVRGKERLVFNYKRLNDNTHKDQYSLPGINTIMQRIGHSKIYSKFDLKSGFHQIAMHPDSIEWTAFWTPQGLFEWLVMPFGLKNAPSIFQRKMDNCFKDTEGFIAVYIDDILVFSQNETEHEKHLRIMLNICRKHGLILSPSKMKIGVPIIHFLGATIGNQKIQLQEHIVKKILHYDDKELMTKKGLRSWLGILNYARNYIPHLGKLTGPLYAKTSPTGEKAM

>Badnavirus03; Aucuba ringspot virus LC487411;

LQDLKDQGFIGDDPLKHWSKNGVHCKLEIINPDITIEDKPPGHLTVEEKARYQAHINILLDLGVIRPSKSRHRTAAFIVKSGTTIDPETGKEKKGKERMVFNYKMLNDNTHKDQYSLPGINSIIKAIGNAKVFSKFDLKSGFHQVAMEESSIPWTAFITPDGLYEWLVMPFGLKNAPAVFQRKMDNCFRGTEDFIAVYIDDILVFSENLQEHEEHLKIMLEICRKNGLVLSPTKMKIACSEIEFLGAVIKRGKIKLQEHIIKRIAQVDDDSLKTLKGLRSWLGVINYARAYIPRCGVLLGPLYSKTSEHGDRRW

>Badnavirus04; Banana streak UI virus HQ593108;

MEEMKELGYIGDEPLKHWNNNKIKCHIRIKNPELTIQDKPQKMVTPQIKEQMKKHMDELLLRRVIRPSTSRHRTNAFIVNSGTSIDPITMKEVKGKPRLVFNYKRLNDNTEKDQYSLPGINALLKSVGNAKIYSKFDLKSGFHQVAMEEESIEWTAFWAITGLYEWLVMPFGLKNAPAIFQRKMDLCFQGTESFIAVYIDDILIFSQNEEEHAEHLYKMMEICKKNGLILSPTKMKIGVKVVDFLGSTVGENHLDLQPHIVQKIVDFDEEKLKTKKGLKSWLAILNYARGHIKNMGKILGPLYPKTSEKGEKRL

>Badnavirus05; Banana streak MY virus KF724854;

FKELKDQGYIGEEPLKHWQKNMVKCKLELKNPDITIQDKPLKHVTAKMRETMRAHIDKLLQLKVIRPSSSRHRTTAMIVESGTEVDPKTGQEKRGKERLVFNFKRLNDNTEKDQYSLPGINTIISRIGNAKVYSKFDLKSGFHQVAMDPESIPWTAFLANNELYEWLVMPFGLKNAPAIFQRKMDTCFKGTEAFIAVYIDDILVFSETEQLHRDHLRKFLEISKANGLILSPTKMKIGVKTIDFLGASIGNSKIKLQPHIIKKIADFDDHRLKETKGLRAWLGILNYARNYIPNLGKTLGPLYSKISPNGEKRM

>Badnavirus06; Banana streak GF virus KJ013507;

IDRLKRLGFIGEEPLKHWKKNQIKCKLEIKNPDLIIEDRPLKHVTPAMKETMTKHVQRLLDIKVIRPSTSKHRTTAIMVNSGTEIDPITGAEKKGKERLVFNYKRLNDNTEKDQYSLPGINTIIARISHSKIYSKFDLKSGFHQVAMEEESIPWTAFWAINGLYEWLVMPFGLKNAPAIFQRKMDNCFRGTEKFIAVYIDDILIFSDSKEAHRTHLRQFITICEENGLVLSPTKMKIGVQQVDFLGATIGDSKVRLQPHIVKKVLETKEESLSETKALRRWLGILNYARAYIPDLGKILGPLYSKTSGKGERKL

>Badnavirus07; Banana streak Peru virus MN187554;

INRMKKLGYIGEEPLKHWEKNQVKCRIEVKNPDMIIEDKPLKHVTPAMKETMAKHVKKLLELKVIRPSQSKHRTTAIIVQSGTEIDPVTGKEKRGKERLVFNYKRLNDNTEKDQYSLPGINTIIRRIGNAKIYSKFDLKSGFHQVAMDPESIPWTAFWAIDGLYEWLVMPFGLKNAPAIFQRKMDSCFRGTEEFIAIYIDDILIFSETVQQHREHLKKFMEICEKNGLVLSPTKMKIGTRQVDFLGATIGNSRIKLQPHIIQKIIDIKDEELKEVKGLRKWLGILNYARSYIPKLGKILGPLYSKTSPNGERRM

>Badnavirus08; Banana streak virus strain Acuminata Vietnam AY750155;

IRKLKDLGYIGEEPLKHWAKNQVKCRIEIKNPDLIIEDRPLKHVTPAMKESMKKHVDKLLELKVIRPSTSKHRTTAIIVQSGTEIDPLTGKEKRGKERLVFNYKRLNDNTEKDQYSLPGINTIISRIGKSKIYSKFDLKSGFHQVAMDPESIPWTAFWAIDGLYEWLVMPFGLKNAPAIFQRKMDNCFRGTEEFIAVYIDDILIFSDNISDHRKHLSKFLEICKANGLVLSPTKMKIGAKEIDFLGATIGNSKIKLQPHIIKKIIETKDEELKETKGLRKWLGVLNYARAYIPNLGKTLGPLYSKTSINGEKKM

>Badnavirus09; Birch leaf roll-associated virus isolate BpubFin407501 MG686420;

MEELKAQGFIGEKPLQHWSLNRIQCKLDIINPHLTIECRPLKHVTPAMKDQFKRHTDQLLKLGVIRPSSSRHRTMAIIVQSGTSIDPKTGKEVRGKERMVLDYRSLNQNTHKDQYSLPGINTIVQRIGNAKVFSKFDLKSGFHQVTMDEESIPWTAFLTPDGLYEWLVMPFGLKNAPAVFQRKMDNCFKGTEEFIAVYIDDILVFSENHKQHEGHLRKMLEIVRQNGLVLSPTKMKIACSEIDFLGATIGNSRIQLQPHIVQKIADKPEKEIMTTTGLRSWLGIINYARNYLPKCGTLLGPLYNKVGAHGDKRW

>Badnavirus10; Blackberry Virus F KJ413252;

LERLKEQGYVGEEPMRHWSKNQVKCKLEIINPDITIQDKPMKHVTPAMKDQFQKHTQALLKLGVIRPSKSRHRTMAMIVYSGTSVDEKTGKEVKGKERMVFNYKTLNDNTFKDQYSLPGINTILQRIGKSKIYSKFDLKSGFHQVAMDEESIPWTAFITPEGLYEWLVMPFGLKNAPAIFQRKMDNCFRGTEEFIAVYIDDILVFSETEEQHAKHLQIMLEICEKNGLVLSANKMKIAVKEVEFLGAVICDRKIKLQPHIIKKITSVPEDSLKEKKGLRSWLGILNYARTYIPKLGTLLGPLYEKTSPHGDKRM

>Badnavirus11; Bougainvillea chlorotic vein banding virus MK473389;

IEDLKAQGIIGEDPLYHWKKNQVKCKLDVINPDITISDKPLKHVSVGLKQQFQNQLDPLLKMGLIRPSTSRHRTMAMIINSGTTIDPVTGQEKKGKERMVFNYKTLNDNTYRDPYSLPGINTIIQKVGRAKIYSKFDLKSGFHQVAMDPESIPWTAFLTPQGLFEWLVMPFGLKNAPAIFQRKMDNCFQKYGDFIAVYIDDILVFSENEKDHEKHLKVMLQICRENGLVLSPTKMKVAVQTIEFLGAILGNQTIKLQPHIIQKIVEFDTEHLQTKQGIRSWLGILNYARAYIPNIGTLLAPFYEKTSPHGDKRL

>Badnavirus12; Cacao Bacilliform SriLanka Virus isolate A MF642736;

LESLAKEGFVGLNPLQHWAKNGIKCKLDIINPSLTIQDKPLKHVTPAMIQSFQKHIKELLDLQVIRPSKSRHRTMAMIVASGTSIDKDGKEVKGKERMVFNYRSLNDNTHKDQYSLPGINTILKKIGEAKVFSKFDLKSGFHQILMDEESIEWTAFTAPSELYEWLVMPFGLKNAPAIFQRKMDFCFKGTEDFIAVYIDDILVFSRSIEEHAKHLEKLIQIIQKNGLVLSPTKMKVASPTIEFLGVIIDRGRIQLQPNIISKIVDFDQKQFADKKGLRSWLGVLNYARSFIPNMGPLLGPLYEKTSPHGDKRM

>Badnavirus13; Cacao red vein-banding virus MH029281;

LTQLKEQGYIGDNPMKHWAKNKIVCRLEIKNPDLVIEDKPIKHLTPAMEQQFKRHIQALLDIGVIRPSKSKHRTTAFIIQSGTTVDPVTKKTIHGKERMVFNYKRLNDNTEKDQYSLPGIQTVLKKVGNSQIFSKFDLKSGFHQVAMAEESIPWTAFWVPQGLYEWLVMPFGLKNAPAVFQRKMDQCFKGTEDFIAVYIDDILVFSNNMTDHIRHIQIMLTICQENGLILSPTKMNLAQKEIEFLGTTITQGKMKLQTHIIKKLVNFANSELETTKGLRSFLGLLNYARIYIPNLGKKLSPLYAKTSPTGERRF

>Badnavirus14; Cacao yellow vein banding virus KX276641;

LQQMEELNFIGPNPQIHWAKNRVVCKIDIINPDLTIEDRPLKHITPEMKAQFSRHTEALLKLGVIRKSSSRHRTNAMIVRSGTSVDPKTGEFRHGKERMVFDYRKLNQNTHKDQYSLPGINTIIKLVGNSKIYSKFDLKSGFHQVAMEESSIPWTAFWVPDGLYEWLVMPFGLKNAPAIFQRKMDNCFKGTEEFIAVYIDDILVFSQTEEEHVRHLQRFFQICQKHGLVLSKDKMAIAVPQIEFLGAIIGNRKIKLQPHIIRKIANFPDDQLKEKKGLRSWLGILNYARNYIPKLGQLLGPLYNKTSPMADKRM

>Badnavirus15; Cacao swollen shoot CD virus NC_038378;

MGQLKDQGFIGENPMKHWASNKIHCKLDIKNPDLIIEDKPIKHLTPAMEKQFAKHIKALLDIGVIRPSKSRHRTTAFIVESGTTIDPKTKKTIHGKERMVFNYKRLNDNTEKDQYSLPGIHTILKRVGNKKIFSKFDLKSGFHQVAMAEESIPWTAFWVPQGLYEWLAMPFGLKNAPAIFQRKMDVCFKGTEDFIAVYIDDILVFSETMEEHENHISRMLEICKRHGLVLSPNKMSIAQEEIEFLGTIISKGRMKLQAHVIKKIVSKAQMELSTTKGLRSFLGLLNYARIYIPNLGRKLSPLYAKVSPTGEKKL

>Badnavirus16; Camellia-associated badnavirus MT036049;

IKALQQQSIFGNDPMRYWQLNQIKCSLDLKNPDLTIDDKPWKHITPALRESFQRHVDELLGLKVIRPSKSRHRTCAFMVQSGTYVDPITKLEKKGKERMVCNYKRLNDNTHKDQYSLPGIDTIVARIGHSKIYSKFDLKSGFHQVAMDPQHIEWTAFWTPVGLYEWLVMPFGLKNAPAVFQRKMDRIFNPYAGFIAVYIDDILIFSPTPEAHKKHLAQFVEICKEHGLVLSPTKMKVAQAQIDFLGSTITNGGLQMQPHIIQKVVDFDEEKLKTTNGLRSWLGLLNYARSYLKNIGVILGPLYSKVSPNGERRM

>Badnavirus17; Canna yellow mottle virus KY9714931;

FRSLKEAGYIGEEPLKHWSQNQIKCRLEIKNPDMVIEDRPLKHVTPKLKEDMQKHIDQLLKLKVIRPSASKHRTTAMLVESGTEVDPKTGLEKKGKQRLVFNYKRLNDNTEKDQYSLPGINTIIQRIGRSKIYSKFDLKSGFHQVAMEAESIPWTAFWAIDGLYEWLVMPFGLKNAPAVFQRKMDSCFRGTEEFIAVYIDDILVFSETPQQHVQHLRKFLEIVKKNGLVLSPTKMKIGVSQIDFLGATIGQSRIKLQPHIIKKVTEFQEEKLNDTKGLRQFLGILNYARNYIPNLGKTLGPLYSKISPKGEKRM

>Badnavirus18; Chestnut mosaic virus MT261366;

IKELERQGYVGEEPLKHWQKNGIKCKLDIINPDITIESKPFGKIPPAMEDQYRRHIDALLKVGVIRPSKSRHRTKAMIVNSGTTIDPKTGKEVRGKERMVLDYRALNMNTHKDQYSLPGITSIIKRVAGSKVFSKFDLKSGFHQVVMEEESIPWTAFVVPGGLYEWLVMPFGLRNAPATFQRKMDQCFKGTEEFIAVYIDDILVFSPDLESHAKHLEKMLAICKDNGLVLSPTKMKVAVKEVDFLGATIRDRKVKLQPHIVKKISEVNEEDLKTLKGLRSWLGVINYARAHIPKCGTLLGPLYQKVGNHGDKRW

>Badnavirus19; Chinaberry tree badnavirus 1 OL630968;

IEELKNQGYIGNEPMKYWIKNQVTCKLDIKNPEMVIEDKPLKHVTPAMEEQFRSHIKALIDLKVIRPSKSRHRTTAFMVNSGTTIDPITKKVVHGKERMVFNYKRLNDLTHKDQYSLPGIHTIMRRIGQAQIFSKFDLKSGFHQVAMDPESVEWTAFWVPDGLYEWLVIPFGLKNAPAIFQRKMDQCFKGTEKFIAVYIDDILVFSKNEEEHAGHVRKMLEIVKSNGLILSPTKMCLAQRSVEFLGAIITGGKLQLQPHIIKKVVQFDKPSLETTKGLRGFLGLLNYARAYIPNLGRLLSPLYAKTSPTGERRF

>Badnavirus20; Citrus yellow mosaic virus EU708316;

IQELKAQGYIGEEPMKYWAKNKVVCHLDIKNPDMVIEDRPLKHVTPQMEESFRKHVEALLKIGAIRPSKSRHRTTAIIVNSGTSIDPITGKEVKGKERMVFNYKRLNDLTNKDQYSLPGIQTILQRLKGSTIFSKFDLKSGFHQVAMHPDSIEWTAFWVPSGLYEWLVMPFGLKNAPAVFQRKMDHCFKGTEAFIAVYIDDILVFSKSEREHEEHLKIMLSICQKNGLILSPTKMKIAQVEIEFLGAIIHNGLIKLQPHIVQKLLTFTNKQLEEVKGLRSWLGLLNYARSYIPHMGRLLSPLYAKVSPTGERRM

>Badnavirus21; Codonopsis vein clearing virus MK044821;

LEELKEQGFIGDDPLKHWSKNEVKCKLDIINPEITIEDKPLSHLTLEDKAAYQRHIDALLALKVIRPSTSRHRTAAFIVKSGTTVDPITGEEKRGKERMVFNYKMLNDNTYKDQYTLPGINSIIKSLGTAKIYSKFDLKSGFHQVVMEEESIPWTAFVCPAGLFEWLVMPFGLKNAPAIFQRKMDNCFKGTEAFIAVYIDDILVFSNSIKEHEEHLKIMLNICKRNGLVLSPTKMKIACTEVDFLGAKITGGKVSLQAHIIKKIAQVDDKDLQSLKGLRSWLGVLNYARNYIPKCGTLLGPLYSKTSEHGDRRW

>Badnavirus22; Commelina yellow mottle virus X52938;

LKEMKEMKYIGENPMEFWKNNKIKCKLNIINPDIKIMGRPIKHVTPGDEEAMTRQINLLLQMKVIRPSESKHRSTAFIVRSGTEIDPITGKEKKGKERMVFNYKLLNENTESDQYSLPGINTIISKVGRSKIYSKFDLKSGFWQVAMEEESVPWTAFLAGNKLYEWLVMPFGLKNAPAIFQRKMDNVFKGTEKFIAVYIDDILVFSETAEQHSQHLYTMLQLCKENGLILSPTKMKIGTPEIDFLGASLGCTKIKLQPHIISKICDFSDEKLATPEGMRSWLGILSYARNYIQDIGKLVQPLRQKMAPTGDKRM

>Badnavirus23; Cycad leaf necrosis virus EU853709;

MERLKKIGIIGEDPLKFWERNKVKCKLDIINPNLTIEDKPLKHVTPAMKEQMRKHIDKLLELKVIRPSKSRHRTTAMIVQSGTEMVLQKDKNGKEKMVEKRGKERLVFNYKRLNDNTEKDQYSLPGINTIIQRIGRSKIYSKFDLKSGFHQVAMDPASIEWTAFWAIDGLYEWLVMPFGLKNAPATFQRKMDMCFAGTEHFIAVYIDDILVFSETEEDHVKHLHQMLHICEKEGLVLSPTKMKIGATSIDFLGATIGRSKIRLQPHIIKKITEAKEEELQTTKGLRAWLGILNYARSYIPNLGKTLGPLYSKVSPTGEKRM

>Badnavirus24; Dioscorea bacilliform AL virus 2 isolate DBALV2-PNG07 DA MH404164;

IKALKEQGFIGEEPMKHWARNKVTCKLDIINPDLTIEDRPLKHVTPQMQEAFKKHVQALLKLKVIRPSQSRHRTTAIIVYSGTTVDPKTGKEVKGKERMVFNYKRLNDNTNKDQYSLPGINTIIQRVGQSKIYSKFDLKSGFHQVAMDPDSIEWTAFWVPEGLFEWLVMPFGLKNAPAVFQRKMDNCFKGTEKFIAVYIDDILVFSQNEQEHEQHLKKMLQICKDNGLVLSPSKMKIGSPRIDFLGAVIGDRRIQLQQHIIKKVAEFSVEELKTTKGLRSWLGILNYARAYIPHLGRLLGPLYSKVSPNGEKRM

>Badnavirus25; Dioscorea bacilliform AL virus isolate DBALV-[2ALa] KX008571;

IIQLKNAGYIGENPMKFWAKNKVQCKLEIINPNLTIQDKPLKHVTPAMEATFKKHVDALLELKVIRPSMSQHRTTAFIVYSGTTVDPATGQEKKGKERMVFNYRRLNDNTEKDQYSLPGINTILKKVGNSKIYSKFDLKSGFHQVAMHPDSVQWTAFCVPGGLYEWLVMPFGLKNAPSVFQRKMDNCFRGTEDFIAVYIDDILVFSRTPEEHAEHLLKMLSICKEHGLVLSPTKMKIGTPTVEFLGATIGNSKIKLQEHIIKKIADFKEEELKTTKGLRSWLGILNYARSYIPNLGKTLGPLYSKISPTGERRM

>Badnavirus26; Dioscorea bacilliform RT virus 2 isolate DBRTV2 NC_038987;

LSRMKQQGYIGDKPMRHWSKNKVMCKLDIINPNLIIQDKPLKHVTPAMTETFKKHIDALLELKVIRPSTSKHRTTAFIVHSGTTVDPETGLEKKGKERLVFNYKRLNDNTEKDQYSLPGINTILKKIGHSKVYSKFDLKSGFHQVAMHPESIEWTAFLTTAGLFEWLVMPFGLKNAPAVFQRKMDNCFRGMEDFLAVYIDDILVFSNSEEEHMRHLEQMLSVCEANGLVLSPTKMKIGTSNIEFLGARIGNHKIQLQEHIIKKIADFPESELMTTKGMRAWLGILNYARSYIPNLGKTLGPLYSKVSPNGEKRM

>Badnavirus27; Dioscorea bacilliform TR virus strain DBV9 NC_038995;

ILELKSAGYIGEEPMKFWSHNKVTCKLEIINPELTIQDKPLKHVTPSMEEAFRKHVQALLKLKVIRPSTSRHRTTAFIVHSGTTVDPKTGVETKGKERMVFNYRRLNDNTEKDQYSLPGINTILKRIGQSVIYSKFDLKSGFHQVAMDPDSVQWTAFWVPDGLYEWLVMPFGLKNAPAVFQRKMDNCFRGTEDFIAVYIDDILVFSRSQEEHAQHLKQMLEICRRNGLVLSPTKMKIGTQVVEFLGAVIGNRKIQLQSHIIKKIADFSDEEIKTTKGLRSWLGILNYARNYIPNLGKILGPLYSKTSPSGEKRM

>Badnavirus28; Dioscorea bacilliform virus clone B39-6 DQ822074;

FQELKAAGYIGDDPLKFWSKNQVVCELNIINPDLTIQNKPLKHVTPAMEETFRKHIDALLKLKVIRASKSRHRTTAFIVYSGTTVDPVTGKENKGKERMVFNYKRLNDNTEKDQYSLPGINTILKRVGQSKIYSKFDLKSGFHQVAMAPQSVEWTAFLAPGGLYEWLVMPFGLKNAPAVFQRKMDNVFRGTEDFIAVYIDDILVFSETEEEHLKHLRILLQICQQHGLVLSPTKMKIGTKTIEFLGAVIGNRKIKLQEHIIKKIADFSDDELKTTKGLRSWLGILNYARAYMPNMGRILGPLYSKVSPNGEKRL

>Badnavirus29; Dioscorea bacilliform virus isolate FJ14 KY827394;

LRALKDQGFIGEEPMKHWQKNQVVCTLDIINPDLVIEDRPLKHVTPQMQESFKKHVQALLKMGVIRPSKSRHRTTAIIVYSGTTVDPKTGKESKGKERMVFNYKRLNDNTNKDQYSLPGINTIIQRVGHSKIFSKFDLKSGFHQVAMDPKSVEWTAFWVPEGLYEWLVMPFGLKNAPAIFQRKMDNCFKGTEKFIAVYIDDILVFSETEKEHKEHLQTMLSICKKNGLILSPTKMKIGCPEIEFLGAIIGNRRIQLQPHIIKKVTDFSEDELKTTKGLRSWLGILNYARAYIPNLGKTLGPLYAKTSPHGEKRM

>Badnavirus30; Dioscorea badnavirus A isolate Won MH898490;

IQELKEQGFVGEEPMKHWSKNKVTCTLDIINPDLAIQDKPLKHVTPQMQESFKKHVQALLKLGVIRPSQSRHRTTAIIVYSGTTVDPKTGQEIKGKERMVFNYKRLNDNTNKDQYSLPGINTIIQRVGNSKIYSKFDLKSGFHQVAMDPKSIEWTAFWVPEGLYEWLVMPFGLKNAPAVFQRKMDNCFRGTEDFIAVYIDDILVFSQDEQAHEGHLRKMLKICKANGLVLSPSKMKIGSSKIDFLGATIGESKIKLQQHIIKKIADFPEQELRTTKGMRSWLGILNYARAYIPHMGRILGPLYSKVSPNGEKRM

>Badnavirus31; Enset leaf streak virus isolate Holetta MF991909;

FRRLKELGYIGEEPLKHWQKNQVRCKLEIKNPDLAIEDRPLKHVTPKMKEQMKKHIDKLLELKVIRPSKSKHRTTAMIVESGTEVDPKTGQETRGKERLVFNYKRLNDNTEKDQYSLPGINTIIQRIGRSKIYSKFDLKSGFHQVAMEEESIPWTAFWAIDGLYEWLVMPFGLKNAPAIFQRKMDNCFRGMEEFIAVYIDDILIFSENPKQHVQHLGKFLEVCEKNGLVLSPTKMKIGAQTVDFLGATIGQSKIRLQPHIIKKIAEFDDNKLKETKGLRSWLGILNYARNYIPNLGKTLGPLYAKVSPTGEKRM

>Badnavirus32; Grapevine badnavirus 1 isolate VLJ-178.Gb1 NC_055481;

INQLKEAGYIGENPLQHWEKNRVVCQLDIKNPDFIIEDKPLKHLTPSMKESFRRHTEALLKLGVIRPSKSRHRTTAMIVQSGTAVDPVTGKETRGKERMVFNYKRLNDLTNKDQYSLPGISTIMKKVGNSRIYSKFDLKSGFHQVAMHPDSIEWTAFWVPDGLYEWLVMPFGLKNAPAVFQRKMDHCFKGTEDFIAVYIDDILVFSENEQDHARHLKIMLQICKENGLVLSPTKMKIAVQEIEFLGAVIGNRKIRLQPHIISKIASFKDEELKERKGLRSWLGLLNYARTYIPNLGRLLSPLYAKTSPTGDKRM

>Badnavirus33; Kalanchoe top-spotting virus AY180137;

IEEMKRLGFIGDEPLKHWRQNKVTCKLEIKNPDLIIEDKPLKHVTPKMKEVMARHVTALLQSKVIRPSTSKHRTTAIIVESGTEVDPITGKEKRGKERLVFNYKRLNDNTEKDQYSLPGINTIIKRIGTSKVYSKFDLKSGFHQVAMAEESIPWTAFWAIDGLYEWLVMPFGLKNAPAIFQRKMDECFRGMEEFIAVYVDDILIFSNSVQDHKRHLQRFFEVCTKEGLVLSPTKMKIGVREVDFLGATIGNSKIKLQPHIITKIIDMRDEEIKETRGLRKWLGILNYARSYIPRLGQTLGPLYSKVSPNGEKRM

>Badnavirus34; Pineapple bacilliform comosus virus GU121676;

YQQLKAEGFIGENPLLHWQRNQVICELQIKNPELTIEDRPLKHVTPALKEAMQKHVDKLLELKVIRPSTSRHRTTAMIVYSGTEVDPVTKKEKRGKERLVFNYKRLNDNTEKDQYSLPGISTILQKIGHSKIYSKFDPKSGFHQVAMHPDSVPWTAFWAINGLYEWLVMPFGLKNAPAVFQRKMDHCFRGTEDFIAVYIDDILVFSETPEQHKKHLEIFLQIARKNGLVLSPTKMKVGVQQVDFLGATIGNSRIRLQPHIIQKVVQFDNKDLQTTKGLRSFLGILNYARSYIPQMGKLLGPLYSKVSPTGEKRM

>Badnavirus35; Piper yellow mottle virus isolate Para MW116777;

IEDMKNQGYIGEDPMKYWQKNQVTCKLEIKNPDLIIEDRPLKHVTPAMHASFLKHIDALLKIKVIRPSKSRHRTCAFIVNSGTSIDPVTGKEVKGKERLVFNYKRLNDNTNKDQYSLPGINTIIXKVGNSKVFSKFDLKSGFHQVAMDPESIEWTAFSTPNGLYEWLVMPFGLKNAPAVFQRKMDNCFKGMEDFIAVYIDDILVFSENMRDHAQHLVAMLEVCKKNGLILSPTKMKIGLGTIDFLGATIGNSKVKLQEHIVKKILDFNTDGLEDKKNLRSWLGILNYARAYIPNLGRILGPLYAKVSPTGERKM

>Badnavirus36; Sugarcane bacilliform Guadeloupe A virus clone 1 NC_038382;

INRMKRLGYIGEEPLKHWEKNQVECRIEVKNPDMIIEDEPLKHVTPAMKETMAKHVKKLLELKVIRPSQSKHRTTAIIVQSGTEIDPVTGKEKRGKERLVFNYKRLNDNTEKDQYSLPGINTIIRRIGNAKIYSKFDLKSGFHQVAMDPESIPWTAFWAIDGLYEWLVMPFGLKNAPAVFQRKMDSCFRGTEEFIAIYIDDILVFSETVQQHKEHLKKFMEICERNGLVLSPTKMKIGTRQVDFLGATIGNSKIKLQPHIIQKIIDIKDEELKEVKGLRKWLGILNYARSYIPKLGKILGPLYSKTSPNGERRM

>Badnavirus37, Sugarcane bacilliform Guadeloupe D virus isolate BataviaD FJ439817;

FRRLKELGYIGEEPLKHWRKNQVKCSLEIKNPDMIIEDRPLKHVTPKMKEQMKKHVDKLLELKVIRPSTSKHRTTAMIVESGTEIDPKTGQEKRGKERLVFNYKRLNDNTEKDQYSLPGINTIIQRIGRSKIYSKFDLKSGFHQVAMEEASIPWTAFWAIDGLYEWLVMPFGLKNAPAVFQRKMDNCFRGTEEFIAVYIDDILIFSESPQQHVQHLKKFMEICEKNGLVLSPTKMKIGVSQVDFLGATIGQSKIRLQPHIIKKIADFEDEKLKETKGLRSWLGILNYARNYIPNLGKTLGPLYSKVSPTGEKRM

>Badnavirus38; Taro bacilliform CH virus isolate Ug10 MG017327;

IQELKDMGYIGDDPMKYWSSNKITCQLEIKNPDLTIEDRPLKHISPQLEASYKRHTEALLKLGTIRPSKSKHRTTAIIVNSGTTIDPVTGKEVRGKERMVFNYKRLNDNTHKDQYSLPGINTIIQKVGNSKIYSKFDLKSGFHQVAMHPDSIEWTAFWVPQGLYEWLAMPFGLKNAPAVFQRKMDNCFKGTEKFIAVYIDDILVFSENEKEHARHLEIMLEICKKNGLILSSSKMKIAVPTIDFLGATIGNRKIKLQEHIIKKIADYPEKELTNTKGLRSWLGILNYARSYIPYLGKQLGPLYSKVSPTGEKKM

>Badnavirus39; Pelargonium vein banding virus GQ428155;

IGRLRNLGFIGENPVKHWARNQVKCRLEIINPDLTIQDKPLKHVTPQMEAQFKRHTDALLQLGVIRPSKSRHRTMAIMVQSGTTVDPATGKETRGKERMVYNYRSLNDNTHKDQYSLPGINTILKKIGTSKVYSKFDLKSGFHQVAMDEESIPWTAFCVPGGLYEWLVMPFGLKNAPSVFQRKMDDCFKGTEAFIAVYIDDILVFSKNEEEHQKHLQKFLEIVEKEGLVLSPTKMKIAVPEVEFLGAIIGNSTIKLQPHIIRKIADIPEEQLKEKKGLRGWLGILNYARTYIPNLSTLLGPLYQKTSPHGDKRL

>Badnavirus40; Epiphyllum badnavirus 1 isolate CA MH396440;

LKRLQKQGIIGTNPLQHWQRNGIKCRLEIINPDITIQDKPLKHVTPLMKDQFKRHTEELLKLKVIRPSTSRHRTMAIMVNSGTTVDPATGEEKKGKERMVFNYKTLNDNTFKDQYSLPGINTIIQKVCNASVFSKFDLKSGFHQIMMDEESIPWTAFLTPDGLYEWLVMPFGLKNAPAVFQRKMDNCFRDLSGFVAVYIDDILVFSNTEDDHAQHLKSMLQVCEREGLILSPTKMKIAAQEIDFLGITLGKQQLKLQPHIITKIAEFNEDLLKEKKGLRSWLGVLNYARLFIPRLGTLLGPLYQKTSPHGDKRM

>Badnavirus41; Fig badnavirus 1 MK348055;

LEELKEAGFIGENPLQHWKKNGILCQLDIKNPDFIIEDRPLKNLTPQMKESFKKHIKVLLDLGVIRASKSRHRTTAMLVNSGTTVDPKTGKEIKGKERMVFNYKRLNDITHKDQYSLPGINTILKKVGNSKIFSKFDLKSGFHQVAMHPDSIEWTAFWVPDGLYEWLVMPFGLKNAPSVFQRKMDECFKGTEDFIAVYIDDILVFSENEKDHAKHLKAMLEICKRNGLVLSPSKMKIAVQEVEFLGAQIGNQRIRLQPHVIKKIVEFNEAELKEKKGMRSWLGILNYARAYIPNLGRLLSPLYAKTSPTGDKRM

>Badnavirus42; Grapevine Roditis leaf discoloration-associated virus MT783680;

IEELKEAGYIGENPLQHWKKNGVLCQLDIKNTDFIIEDRPLKSVTPQMKESFKKHVKALLDLKVIRPSKSRHRTTAMLVNSGTSVDPKTGKETKGKERMVFNYKRLNDITHKDQYSLPGINTILKRIGNSKIFSKFDLKSGFHQVAMHPDSIEWTAFWVPDGLYEWLVMPFGLKNAPSIFQRKMDGCFNGTEEFIAVYIDDILVFSDDEKSHAKHLSIMLEICKKNGLVLSPTKMKIAVQEVEFLGAQIGNQKIKLQPHVIKKIVEFNEAELREKKGMRSWLGILNYARAYIPNLGRLLSPLYAKTSPTGDKRM

>Badnavirus43; Jujube associated badnavirus MN274946;

LDRLKEQDFIGEDPLKHWEANGIKCHIDIINPDITIQDPPLKHVTPALKETFQKHIDALLKLGVIRESNSRHRTMAMIVKSGITIDPITGKEQKGKERMVFNYRTLNDNTYKDQYSLPGINTILKKVGNAKIYSKFDLKSGFHQIAMDKESIPWTAFIVPQGLFEWLVMPFGLKNAPALFQRKMDHCFKGTEDFIAVYIDDVLIFSASEQEHHKHLQIMLDRCKKHGLILSPTKMVIAVPEVYFLGAVLGKQKLKLQPHIIQKIANFKDKDLQEKKGLRSWLGILNYARSYIPKLGAKLGPLYEKTSPHGDKRF

>Badnavirus44; Jujube mosaic-associated virus KX852476;

LDRLKEQGYIGDNPLQHWEKNQLKCHIDIINPDITIQDPPLKHVTPALKETFQKHIDALLKLGVIRESSSRHRTMAMIVKSGTTVDPITGQEQKGKERMVFNYRTLNDNTYKDQYSLPGINTILKKVGNSKIYSKFDLKSGFHQIAMEKESIPWTAFIVPQGLYEWLVMPFGLKNAPALFQRKMDNCFKGTEDFIAVYIDDVLVFSNNEQDHHRHLHVMLNICKQHGLILSPSKMMIAVPEVYFLGAVLGKQKLKLQPHIIQKIANFKDADLQEKKGLRSWLGILNYARSYIPNLGSKLGPLYEKTSPHGDKRF

>Badnavirus45; Pitaya badnavirus 1 MK991812;

LKRLQKQGIIGTNPLQHWQRNGIKCRLEIINPDITIQDKPLKHVTPLMKDQFKRHTEELLKLKVIRPSTSRHRTMAIMVNSGTTVDPATGEEKKGKERMVFNYKTLNDNTFKDQYSLPGINTIIQKVCNASVFSKFDLKSGFHQIMMDEESIPWTAFLTPDGLYEWLVMPFGLKNAPAVFQRKMDNCFRDLSGFVAVYIDDILVFSNTEDDHAQHLKSMLQVCEREGLILSPTKMKIAAQEIDFLGITLGKQQLKLQPHIITKIAEFNEDLLKEKKGLRSWLGVLNYARLFIPRLGTLLGPLYQKTSPHGDKRM

>Badnavirus46; Yacon necrotic mottle virus KM229702;

LKELKQQGXIGEEPLKHWRKNGETCKLDIINPDITVQDKPLKHVTPALEASFKKXIEALLKLKVIRPSKSRHRTMAMIVNSGTTVDPATGKETKGXXRMVFNYRTLNDNTYKDXYSLPGINTLLKRIGNAKIFSKFDLKSGFHQVAMEEESIPWTAFLIPGGLYEWLVMPFGLKNAPAIFQRKMDKCFKDTEEFIAVYIDDILVYSNSEADHERHLKIMLGKCQENGLVLSPTKMKIAVPEVEFLGAIIGRNKIKLQPHIIKKICDFDEEKLKTKAGLRSFLGILNYARNYIPRLSILLGPLYEKTNPHGDKRM

>Badnavirus47; Dracaena mottle virus DQ473478;

IVQRLLQQNISDDPLKFWAKNKVTCQLEIINPDLTIQDKPLKHVTPLMEQQFKRHVEALLQLKVIRPSKSRHRTMAMIGNSGTSVDPTTGKEVKGKERMVFNYRSLNDNTHKDQYSLPGINTIIQKIGRATVYSKFDLKSGFHQVAMSPESIEWTAFIVLGGLYEWLVMPFGLKNAPAVFQRKMDHCFAGTEKFIAVYIDDILIFSANDEEHVEHLKVFCAIVEKHGLILSSNKMQLGKREIDFLGATLGNRKIKLQAHIIKKIAAFPEAQLAEKKGLRSWLGILNYARSYIPRLGILLGPLYQKTSPHGDKRM

>Badnavirus48; Lucky bamboo bacilliform virus EF494181;

IVQRLLQQNISDDPLKFWAKNKVTCQLEIINPDLTIQDKPLKHVTPLMEQQFKRHVEALLQLKVIRPSKSRHRTMAMIGNSGTSVDPTTGKEVKGKERMVFNYRSLNDNTHKDQYSLPGINTIIQKIGRATVYSKFDLKSGFHQVAMSPESIEWTAFIVPGGLYEWLVMPFGKNAPAVFQRKMDHCFAGTEKFIAVYIDYILIFSANDEEHVEHLKVFCAIVEKHGLILSSNKMQLGKGEIDFLGATLGNRKIKLQAHIIKKIAAFPEAQLTEKKGLRSWLGILNYARSYIPRLGILLGPLYQKTSPHGDKRM

>Badnavirus49; Canna yellow mottle-associated virus KX066020;

FKELKENGYMGEEPLKHWKKNQIKCKLEIKNPDLIIEDRPLKHVTPKMKEDMAKHVNQLLKLGVIRPSNSKHRTTAMLVESGTEVDPKTGEEKRGKQRLVFNYKRLNDNTEKDQYSLPGINTIIQRIGRSRVYSKFDLKSGFHQVAMEEESIPWTAFWAIDGLYEWLVMPFGLKNAPACFQRKMDNCFRGKEHFIAVYIDDILIFSENKEQHVQHLKEFLRIVKKEGLVLSPTKMKIGVPKVDFLGATIGESRIKLQPHIIKKVVNFKNEDLKETKGLRSFLGILNYARNYIPNLGKTLGPLYSKTSPNGERRM

>Badnavirus50; Gooseberry vein banding associated virus MZ220959;

LAELKEQGFIGNDPMLHWAKNQVKCKLDIINPDITIQGKPPSTATPEIKDRYQRHIDALLSIGVIRPSKSRHRTAAFITHSGTSVDPKTGEEIRGKERMVFDYRALNNNTHKDQYTLPGINSIVAAVGNAKIYSKFDLKAGFHQVLMEESSIPWTAFITPVGFYEWLVMPFGIANAPAVFQRKMDNCFHKLREFVAVYIDDILVFSNSLQEHESHLRQMLEVCRKNGLVLSPTKMKVAVTTVEFLGAIIGNGKIKLQPHIVKKISEVDDESLRTLKGLRSWLGIINYARNYIPNCGTLLGPLYSKTSENGDRRM

>Badnavirus51; Grapevine vein clearing virus KJ725346;

LAELKEQGYIGEEPLKHWSKNKVRCKLDIINPDITIEAKPPGHLTLEDKVKYQKHIDALLDLGVIRPSKSRHRSAAFIVASGTSVDPKTGKETRGKERMVIDYRMLNDNCHKDQYSLPGITSIIKSLGQAKIFSKFDLKSGFHQVMMEEESIPWTAFISPAGLYEWLVMPFGIQNAPAIFQRKMDECFKGTEDFIAVYIDDILVFSNSIKEHEKHLQRMLSICKEHGLVLSPTKMKIAVPGIDFLGAHIRNSRVSLQPHIIKKIADKKDDELMTLKGLRSWLGVINYVRQYIPKCGTLLGPLYAKTSEHGDRRW

>Badnavirus52; Hibiscus bacilliform virus KF875586;

MKELKDQGYIGENPMKHWARNKVLCYLDIKNPDMVIEDKPIKHVTPQMEESFRKHIKGLLELKVIRPSTSKHRTTAFIVNSGTSVDPVTGKETKGKERMVFNYKRLNDLTEKDQYSLPGINTIMKRVGHAKIYSKFDLKSGFHQVAMHPESIKWTAFWVPDGLYEWLVMPFGLKNAPAVFQRKMDNVFKGTEAFIAVYIDDILVFSQSEEEHIKHIRVMLEKCRENGLVLSPTKMKIAQRKVEFLGAILEAGRIQLQPHIIKKVVNFKDEDLQETKGLRSFLGLLNYARAYIPQMGRLLSPLYAKVSPKGEKKL

>Badnavirus53; Sweet potato badnavirus B MT587568;

VARLIQEGYIGENPLRHWSKNKVECTLRIKNPDLVIQDPPLKHVTPAAREFFQNQVSSLIKAQLIRPSRSRHRTTAFMVESGTSVDPKTGKEVRGKQRMVLNYKRLNDNTEKDQYSLPGINTIISRVAGKKVFSKFDLKSGFHQIRMSKESIPWTAFWTPDGLYEFLVMPFGLVNAPADFQRKMDNAFRGTEAFIAVYIDDILIFSETEEDHEQHLLKFAQIVEKNGLILSPTKMKIGVKSVDFLGVKIHQNKVQLQEHILKKIGDFREEDLLTKKGLRSWLGILNYARQHIPNLGKMLGPLYGKTSPTGEIRF

>Badnavirus54; Ivy ringspot-associated virus NC_055604;

IKELSSMGYIGNDPLLHWGKNQVKCRLEIKNPDLKIDDKPLKHVTPQMSESFKKHVDELLKLKVIRPSNSPHRTTAFIVNSGTTVDPKTGKETKGKERMVFNYQRLNDNTEKDQYPLPGINTIIQKIGRSKIYSKFDLKSGFHQIAMEPESIPYTAFTIPGLGLFEWLVMPFGLKNAPSIFQRKMDNCFKGTEAFIAVYIDDILVFSETPEEHVKHLKVLFRIVKENGLVLSPTKMKIGVKQIEFLGAVITNGCLSLQENILKKIAAFGPEQYQTKKDLRSWLGLVNYARIYIPNLGRILGPLYSKTSPQGEARM

>Badnavirus55; Mulberry badnavirus 1 LN651258;

IEELKEQGTVGENPLQHWERNRVHCYLDIKNPDLTVQDKPLDQITPVQKEMYKKHIDALLQIGVIRRSNSRHRTNAFIVHSGTTVDPRTGEETKGKERMVFNYKRLNDLTHKDQYSLPGIQGIIARVGRAKIFSKFDLKSGFHQVAMHPESIPWTAFWVPQGLYEWLVMPFGLKNAPAIFQRKMDNCFMGTEEFIVVYIDDILVFSQNEQDHERHIRAMLKICKENGLILSPSKMKIGQTKVEFLGAIIDKGKIRLQPNVIKKVCDFKMEHLETKTGLRSWLGLLNYARPYMPDLGKMLGPLYAKVSPNGERRF

>Badnavirus56; Pagoda yellow mosaic associated virus KJ013302;

LKDLMAQGYIGEDPVRHWVKNQVICRLDIINPDITIQSQPLKHVTVEMERSFQTHVDGLLKLKVIRPSKSRHRTLAILVKSGTSIDPLTGKEVKGKERMVYDYRQLNNNTHKDQYSLPGINTIIQKVGRAKVYSKFDLKSGFHQVAMDEASIPWTAFLVPGGLYEWLVMPFGLRNAPAIFQRKMDEVFADLKEFVSVYIDDILVFSETYEEHAAHLKRMLQRCKKFGLVLSPTKMKIATREIDFLGATIKDGRIKLQDHIIKKISTVDEKSLETTKGLRSWLGIINYARGYIPNCGTLLGPLYSKVGLHGDKRW

>Badnavirus57; Paper mulberry vein-banding virus MW052244;

LNRMEELEYIGKNPQKHWSKNSVVCRIDIINPDISIEDKPLKHVTPMMKESFKKHVDALLQIGVIRPSKSRHRTMAMIVYSGSTLDPKTGQTIHGKERMVFDYRKVNQNTHKDQYSLPGINTLIKKVGRARIYSKFDLKAGFHQVAMAEESIPWTAFVVPQGLFEWLVMPFGLKNAPAIFQRKMDKCFEHCSNIVAYIDDILVFSETKEEHRKHLQEFLKIARDQGLVLSKEKMMIGVSEIEFLGVVLGKSQVKLQPHIIKKLTQVEEKQLLEKKGLRSWLGILNYARPFIPNIGKILAPLYNKTSNTSPIRL

>Badnavirus58; Polyscias mosaic virus NC_055562;

IKRLTSMGYIGMKPLQHWEKNGVLCHLELKNPNLKIEDRPLKHVTPQQQDSFRKHIEELLQLGVIRPSTSPHRTTAFLVNSGTSIDPKTGKEVKGKERMVFNYQRLNDNTEKDQYSLPGITTIHRRIAHSKIYSKFDLKSGFHQVAMHPDSIPYTAFTVPGGGLYEWLVMPFGIKNAPGIFQRKMDFCFAGTEDFIAVYIDDILVFSKNEKDHLRYLEIMFSLVEKHGLVLSPTKMKIGVHSIGFLGAQIEDGTLKLQEHILKKILKFGPAQFATKKDLRSWLGILNYSRNYIANLGKLLGPLYAKTSPQGEIRM

>Badnavirus59; Rubus yellow net virus KF241951;

LQELKEQGYIGEEPMKHWAKNGIKCKLDIKNPDIVISSKPPDAVSKETKAQYQRHIDALLKIKVIQPSKSRHRTAAFITNSGTTVDPITKKEIRGKERMVFDYRSLNDNTHKDQYTLPGINTIISAIGNAKIFSKFDLKSGFHQVLMDEESIPWTAFVTPVGFYEWKVMPFGLANAPAVFQRKMDQCFAGTSEFIAVYIDDILVFSKTLKEHEKHLSIMLGICRDNGLVLSPSKMRLAATEIDFLGASIGDGKIKLQPHIIKKIAEVDDESLKTLKGLRSWLGVLNYARNYIPKCGTLLGPLYSKTSEHGDRRW

>Badnavirus60; Spiraea yellow leafspot virus MW080370;

LEELKQQGFIGDDPMKHWAKNQIRCKLEIINPDITIQGKPPSTVTPEEKARYQRHIDALLQIGVIRPSKSRHRTAAFITYSGTSVDPVTKKETRGKERLVFDYRALNANTHKDQYTLPGINSIVSAIGNAKIFSKFDLKAGFHQVLMDEQSIPWTAFITPVGFYEWLVMPFGIANAPAIFQRKMDNCFNRCKEFIAVYIDDILVFSNTLQEHEKHLQKMLEICRANGLVLSPTKMKVAVTEVDFLGATIGAGSIKLQSHVIKKIAEVDDESLKTLKGLRSWLGVLNYARNYIPKCGTLLGPLYSKTGEHGDRRM

>Badnavirus61; Sweet potato pakakuy virus FJ560943;

VAALIKEGFIGNNPLLHWTKNRVYCKLQIKNTDLIIQDPPLKHVTPAAREFFKSQISDLLKAKLIRPSKSKHRTTAFMVESGTIVDPKTGKEIRGKQRMVYNYKRLNDNTEKDQYSLPGINTIVSRISGKKIFSKFDLKAGFHQIRMEEKSKPWTAFWTPEGLYEFEVMPFGLMNAPADFQRKMDNAFRGTDAFIAVYIDDILVFSENEEEHEDHLLNLAQIVRREGLILSPTKMKIGVKEVDFLGIKIQGNKIQLQEHILKKIGDFKEKDLLTKKGLRSWLGILNYARQYIPNLGKLLGPLYGKTSPTGEIRF

>Badnavirus62; Wisteria badnavirus 1 KX168422;

LADLKAQGFIGEDPVKHWKKNQVICKLEIINPDITIQAQPLKHVTAEMEKSFKTQVDGLLKLRVIRPSKSRHRTLALLVKSGTSIDPLTGKEVKGKERMVYDYRQLNNNTHKDQYSLPGINTIIQKVGRAKVYSKFDLKSGFHQVAMDEDSIPWTAFLVPGGLYEWLVMPFGLRNAPAIFQRKMDEVFGDLKDFIAVYIDDILVFSETHEQHAQHIKRMLLRCKKHGLVLSPSKMKIAQKEIEFLGATLTGGQIKLQDHIVKKIAAVDDKSLQTTKGLRSWLGIINYTRAYIPNCGTLLGPLYSKVGLHGDKRW

>Badnavirus63; Cacao swollen shoot Ghana J virus NC_040807;

LDKLKEQGYIGENPLKHWANNKIFCKLEIKNPDLIIEDKPIKHLTPSMEQKFQKHIKSLLDIGVIRPSKSKHRTTAFIVESGTSYDPVTKQTIHGKERLVFNYKRLNDNTEKDQYSLPGIQTILKKVCNMKIFSKFDLKSGFHQVAMAEESIPWTAFWVPQGLFEWLVMPFGLKNAPAVFQRKMDQCFKGTEDFIAVYIDDILVFSKTMKEHMKHLHQLLHICQKNGLVLSPNKICLAQEEMEFLGTVISHGRMKLQQHVVKKILEKSNLELETTKGLRSFLGLLNYARIYIPNLGRMLSPLYAKTSPTGERKM

>Badnavirus64; Green Sichuan pepper vein clearing-associated virus MK371354;

LKDLKDQGYIGEDPVRHWAKNGVKCKLEIINPDITIEDKPPGDLSVEEKARYQRHIQVLLDLGVIRPSKSKHRTAAFIVKSGTTVDPITGKETKGKERMVYNYKMLNDNTHKDQYTLPGINSIIKSLGGAKIFSKFDLKSGFHQVAMDEESIPWTAFISPAGLYEWLVMPFGLKNAPAVFQRKMDECFKGTEEFIAVYIDDILVFSSTIKEHEKHLQVMLEICQKHGLVLSPTKMKIACREVEFLGAIIREGKIKLQPHIIKKIADVEEKSLLTLKGLRSWLGVLNYARAYIPKCGTLLGPLYSKTSEHGDRRW

>Badnavirus65; Taro bacilliform virus AF357836;

MNELKAAGYIGEDPLKHWSKNKVTCKLDLKNTEITIQDKPLRHITPALEQSYGRHVNALLMLKVIQPSKSRHRTMAFLVNSGTTVTADGKEIKGKERMVFNYKALNDNTYKDQYSLPNIQLILKKVINSTIYSKFDLKSGFHQVAMDPDSVEWTAFLVPQGLYEWLAMPFGLKNAPAVFQRKMDAVFKGCEKFLAVYIDDILVFSNNEEDHAKHLVIMLQRCKEHGLVLSPTKMNIAVREVNFLGATIGSRKVKLQENIIKKILDFDTEKLQSKKGLRSFLGILNYARNHIPNLGKIAGPLYSKTSIYGDIRF

>Badnavirus66; Cacao mild mosaic virus KX276640;

LLQLSDMGYIGEEPLKHWKKNQVECKLEIINPQLKIEDKPLKHITPQMQESFNKQVESLLKLGVIRPSKSQHRTTAFLVKSGTTVDPKTGKETKGKERMVFNYQRLNDNTEKDQYPLPGINTILQRIGKTKIYSKFDLKSGFHQVAMAEESIPWTAFAIPGKGLFEWMVMPFGLKNAPSVFQRKMDLCFTGLEKFVAVYIDDVLIFSNSEQEHLEHLDRFFERVQQHGLVLSPTKMKIGVRKVDFLGTVISNGLLHMQEHILKKIAAFGPEQYKSKKDLRSWLGLINYARNHIPNVGRMLGPIYAKTSPQGEPKM

>Caulimovirus01; Angelica bushy stunt virus NC_043523;

IDNLLEKVCSDNPLDPSRTKHWMTASIKPIDPRKVCKVKPMKYSPQDREEFAKQIKELLEMKIIVPSKSAHMSPAFLVENEAEKRRGKKRMVVNYKRMNDLTVGDGHNLPNKDELLTLIRGKKVFSSLDCKSGFWQVLLDEDSQLLTAFTCPQGHYQWRVLPFGFKQAPSIFQRHMQNALNSYEKFCCVYVDDILIFSDNEEDHQKHLAAVLKKCEQLGIILSKKKAQLFKTKINFLGLEIDQGTHRPQNHILEHIAKFPDKIEDKKQLQRFLGILTYASDYIPGLASKRAPLQVKLKKDISWNW

>Caulimovirus02; Atractylodes mild mottle virus KR080327;

IEKLLDEVCSENPIDPHKSKKWMTASIKLKDPNTLVQVKPMQYCPEDRKEFAVQIKELLDLKIIIPSKSPHRSPAFLVENEAERRRGKKRMVVNYKKLNEVTLGDSHNLPNKDELLTLIRGKTIYSSFDCKSGFWQVLLDQESQLLTAFTCPTGHYQWKVVPFGLKQAPSIFQRHMNNAFREFEEFCCVYVDDILVFSKNETEHRKHVIAILEQCKNLGIILSKKKAHLFKTKINFLGLEIDQGSHKPQNHILEHIHKFPDRLEDKKQLQRFLGILTYASDYIPKLAQIRKPFQAKLKKDVTWSW

>Caulimovirus03; Carnation etched ring virus AJ853858;

IEEMLERVSSENPIDPEKSKQWMTATIELIDPKTIVEVKPMSYSPSDREEFDKQIKEPLDLKVIKPSKSPHMSPAFLVENEAERRRGKKRMVVNYKAMNKATKGDAHNLPNKDELLALVRGKKIYSSFDCKSGFWQVLLDKESQLLTAFTCPQGHYQWNVVPFGLKQAPSIFQRHMQTAFNQHSKYCCVYVDDILVFSNTEEEHYIHVLNILRRCEKLGIILSKKKAQLFKEKINFLGLEIDQGTHCPQNHILEHIHKFPDRIEDKKQLQRSLGILTYASDYIPKLASIRKPLQSKLKEDSTWTW

>Caulimovirus04; Cauliflower mosaic virus M90543;

IEELLEKVCSENPLDPNKTKQWMKASIKLSDPNKAIKVKPMKYSPMDREEFDKQIKELLDLKVIKPSKSPHMAPAFLVNNEAEKRRGKKRMVVNYKAMNKATVGDAYNLPNKDELLTLIRGKKIFSSFDCKSGFWQVLLDQESRPLTAFTCPQGHYEWNVVPFGLKQAPSIFQRHMDEAFRVFRKFCCVYVDDILVFSNNEEDHLLHVAMILQKCNQHGIILSKKKAQLFKKKINFLGLEIDEGTHKPQGHILEHINKFPDTLEDKKQLQRFLGILTYASDYIPKLAQIRKPLQAKLKENVPWKW

>Caulimovirus05; Dahlia common mosaic virus LC625373;

IQNLLEKVCSENPIDPAKSKAWMKASIKLADPKSVVRVKPMVYSPEDRKEFEIQIKELLDLKVIEPSKSQHMSPAFLVEKEAEKRRGKKRMVVNYKKLNEVTIGDSHNLPNMQELITLLRGKTIFSSFDCKSGFWQVFLDQESQKLTAFTCPQGHFQWRVVPFGLKQAPSIFQRHMQNALRGLEEFCLVYVDDIIVFSDKEEEHYTHVLKVLKRIESLGIILSKKKANLFKEKINFLGLEIDRGTHTPQNHILEHLHNFPDRLEDKKQLQRFLGVLTYADSYIPKLAEKRKPLQVKLKKDQVWIW

>Caulimovirus06; Dahlia mosaic virus JX272320;

IQELLKRVCSENPIDPQKSKGWMTASIKLADPKSVVKVKPMVYSPQDRKEFEIQIKELLNLKVIIPSKSQHMSPAFLVEKEAEKRRGKKRMVVNYKKLNEVTIGDSHNLPNMQELITLLRGKSIFSSFDCKSGFWQVFLDQESQKLTAFTCPQGHFQWRVVPFGLKQAPSIFQRHMQNALRGLEDYCLVYVDDIIVFSNSEKEHYDHVLSVLRRVESLGIILSEKKANLFKEKINFLGLEIDRGTHTPQNHILEHLHGFPDRLEDKKQLQRFLGVLTYADSYIPKLAEKRKPLQVKLKKDQVWSW

>Caulimovirus07; Eupatorium vein clearing virus EU569831;

IEELLELACSENPLDPEKSKGLLTASIKLIDPNKIIRVKPIPYPPNIRQEFDIQIKELLAMNLIVPSKSPHMSPAFMVNKGAEQRRGKMRMVVNYKALNDATIGDAHNIPNRDSLMALISGKRIFSSFDCKSGFWQVLLDKPSQELTAFTCPQGHYQWLVMPFGLKQAPAIFQRHMQIALNEHSAYSCVYIDDILVFSENEKDHEIHVSKVLNRCINLGIILSKKKSQLFKETIDFLGISIDKGTHSPKPHILENIHNFPERFKDVNQCRKFLGIITYAMRYIPELSRKRMFLQDKLKKNVPWTW

>Caulimovirus08; Figwort mosaic virus X06166;

IEQLLDKVCSENPIDPIKSKQWMKASIKLIDPLKVIRVKPMSYSPQDREGFAKQIKELLDLGLIIPSKSQHMSPAFLVENEAERRRGKKRMVVNYKAINQATIGDSHNLPNMQELLTLLRGKSIFSSFDCKSGFWQVVLDEESQKLTAFTCPQGHFQWKVVPFGLKQAPSIFQRHMQTALNGADKFCMVYVDDIIVFSNSELDHYNHVYAVLKIVEKYGIILSKKKANLFKEKINFLGLEIDKGTHCPQNHILENIHKFPDRLEDKKHLQRFLGVLTYAETYIPKLAEIRKPLQVKLKKDVTWNW

>Caulimovirus09; Horseradish latent virus JX429923;

IEELLEKVCSENPLDPEKSKGWMQASIKLSDPTKVIKVKPMKYSPMDREEFEKQIQELLDLKVIRPSKSPHMAPAFLVNNEAEKRRGKKRMVVNYKAMNDATVGDAYNLPNKDELLTLIRGKKIFSSFDCKSGFWQVRLDEESKSLTAFTCPQGHYEWNVVPFGMKQAPSIFQRHMDEAFKVFRKFCCIYVDDILVFSDNEQNHQLHVAMILQKCYQHGIILSKKKAQLFKERINFLGLEIDQGTHRPQSHILEHIQKFPDIIESKLQLQRFLGVLTYASDYIPKLAQIRKPLQAKLKENVQWRW

>Caulimovirus10; Isatis caulimovirus A MH898528;

IEELLDRVCSENPLDPQKSKQWMKASIKLSDPTKVVKVKPMKYSPMDREDFEKQIQELLDLKVIRPSKSPHQAPAFLVNNEAEKRRGKKRMVVNYKAMNNATIGDAYNLPNKDELLTLVRGKKIFSSFDCKSGFWQVLLDEDSKPLTAFTCPQGFYEWNVVPFGMKQAPSIFQRHMDDAFKVFRKFCCVYVDDILVFSDNEEDHLLHVAMILQKCFQHGIILSKKKAHLFKEKINFLGLEVDEGTHKPQAHILEHIHKFPDILEDKKQLQRFLGILTYASDYIPKLAQIRKPLQAKLKENVPWKW

>Caulimovirus11; Lamium leaf distortion associated virus EU554423;

IEKLLDQVCSENPLDPLKTKKWMKASIKLIDPKTVVKVKPMRYNPQDVEEFAKQIKELLELKIIIPSKSPHQSPAFLVENEAERRRGKKRMVVNYKAINTATIGDAHNLPNKDELLTLIRGKSIFSSFDCKSGFWQVLLDEDSQLLTAFTCPQGHYQWIVVPFGLKQAPSIFQRHMNNAFRDFASYCCVYVDDILVFSNNIKDHYAHVAQVLRKCAELGIILSKKKAQLFKCRINFLGLDIDEGTHRPQNHILEHIHKFPNKIEDKKQLQRFLGILTYASDYIPQLASMRAPLQEKLKEDVPWNW

>Caulimovirus12; Metaplexis yellow mottle-associated virus MW656214;

LELLLSDVCSEHPQDPAISKGKFEARIELIDSSKVIKVKPMQYTPENRIEFGKQITELLNLGVITKSKSPHFSPAFLVMNHSEKKRGKARMVINYKALNAATKGDGYLLPNKDQILQRIGGKKWFSSFDCKSGFWQVRLHPESQELTAFTCPQGHYHWKVMPFGLKQAPSIFQRHMDESFLDLTKFCLVYVDDILIFSDNESSHHDHVKQVLNRCKELGIILSEKKAQLCKTKIDFLGLTIDAGTLVLQRHIGEHLQEFPDHIQDRKSLERFLGTLNYISGYFPKIAQLRQPLQAKLKKEVQWTW

>Caulimovirus13; Mirabilis mosaic virus AF454635;

IEQLLEKVCSENPIDPEKSKGWMKAEIKLIKPETVVRVKPMVYSPEDRKEFEIQIKELLDLKVIIPSKSQHMSPAFLVEKEAEKRRGKKRMVVNYKKLNEVTIGDSHNLPNMQELITLLRGKNIFSSFDCKSGFWQVLLDDESQKLTAFTCPQGHYQWRVVPFGLKQAPSIFQRHMQDALRGLEEFSLVYVDDIIVFSDNKNDHQDHVMKVLRRIESLGIILSKKKANLFKEKINFLGLEIDRGTHTPQNHILDHIHTFPDRIEDKKQLQRFLGVLTYADSYIPKLAEKRKPLQVKLKKDQVWIW

>Caulimovirus14; Silene caulimovirus A MH898523;

IEELLENVCSENPIDPVKSKQWMTASIKLKDPTTIVKVKAMNYSPSDVKEFEKQIKELLDLKVIKKSYSPHQSPAFLVENEAERRRGKKRMVVNYKAMNDATVGDAHNLPNKDGLLSLVRGKKIFSSFDCKSGFWQVLLDPESQVLTAFTCPQGHYQWNVLPFGLKQAPSIFQRHMQTALNEFSSFCCVYVDDILVFSNTEDEHYLHVLRVLKKCEQLGIILSKKKAQLFKEKINFLGLEIDQGTHCPQHHILEHIKKFPDRIEDKKQLQRFLGILTYASDYIPKLAKIRQPLQAKLKEDQPWVW

>Caulimovirus15; Soybean mild mottle pararetrovirus JQ926983;

IHNLLDRVCSENPIDPEKSKRWMTASIKLKDPNTIVKVKPMQYNPDDRMEFSKQIKELLDLKIIIPSKSPHQSPAFLVENEAERRRGKKRMVVNYKKINEATIGDSHNLPNKDELLTLIRGKTIYSSFDCKSGFWQVFLDQESQLLTAFTCPSGHYQWRVVPFGLKQAPSIFQRHMQNAFRSFEKFCCVYVDDILVFSDNEQDHHRHVMALLQRCNQLGIILSKKKAQLFQEKINFLGLEIDQGSHKPQNHILEHIHKFPDKIEDKKQLQRFLGILTYASDYIPKLAEIRKPLQSKLKKDVPWTW

>Caulimovirus16; Strawberry vein banding virus MT731326;

VEKLLDSICSEHPLDKRINKGKFEAQITLLDPNKVIKCKPMQYSPQDREEFKTQIEELLKLGIIRPSKSPHSSPAFMVRNHAEIKRGKARMVINYKKLNDNTKGDGYLLPNKEQLLQRIGGKTYYSSFDCKSGFWQVRLAPETIQLTAFSCPQGHYEWLVMPFGLKQAPAIFQRHMDESLSNMYPSFCAVYVDDIIVFSKTEDEHLGHVRIVLNRCKALGIVLSKKKAQLCKTTINFLGLVIERGNLKVQSHIGLHLTAFPDQLADRNALQRFLGLLNYISAYFPKIANLRSPLQVKLKKEITWSW

>Vaccinivirus01; Blueberry fruit drop associated virus KT148886;

LTKQLEPNISDNPIHKWDVTQTYADIQLKDPYALIRVKPMPYSAEDEAEFERQLEEQLKLELIQPSKSPHSSPAFCVRNHAEIKRQKARIVINYKELNRVTKDDGYFLPNLETLVYQVAEARVFSKFDCKSGYWQIKLTPESIPLTAFSTPKGQYEWKVLPFGLKNAPNIFQRRMDKIFKDCYQFCGVYVDDILVFSKDKEEHLLHLDIIINKIVQNGIIIGRTKYELVRESIDFLGVTICKGKIKLQPHILTHLMEFPDKLKDRLQVQQFLGCVNYAGKFIQRLAELCSVLHTKLKKDHKWKF

>Soymovirus01; Malva associated soymovirus 1 OL451868;

IDEKLLEVCSEDPLDLKKNTNNELVEIKLKDPTAEVNVPNRIPYTIKDVEEFTKECETLLEKGIIRPSKSPHSAPAFYVENHNEIKRGKRRMVINYKKMNDATIGDPYSLPRKDFILEKVKGCRWYTSLDAKSGYWQLRLHENTKPLTAFSCPPQKHYEWNVMPFGLKQAPAIYQDFMDRNLEGLEQFCLAYIDDILIFTKKDKIDHLEKLEIVLNRIKERGLIISQKKSKIACEELEYLGLILGKDGKIDLAAHVKEKLDAFPNELEDRKQIQRFLGVLNYVADQGFVKNLASLRKELQKKLKKGIHFQW

>Soymovirus02; Blueberry red ringspot virus MN380637;

VNKILQERFSLDLLGEKNKNKELIEIKLKDPNAEIFVPNNIPYTQRDIEEFKEDMEDLINKGLIRPSKSPHSAPAFYVENHSEIKRAKRRIVINYKAMNEATIGTPKTLPRSDYIMNRLKGKIWFSTLDVKSAYWQLRLTEESKPLTAFSYPPQKHYEWNVLPMGLKQAPGIFQEFMNRSLHNLEHICLVYVDDIIIFSEKDKNDHLSKVLQVLKRCADEGIILSQPKAKIAHKEIDFFGLHISEGEIILQPHILEKLVLFPDEIENRKQLQRFLGNLNYISEKGFIKDFAKYRKELQKKVSEKVPWKW

>Soymovirus03; Cestrum yellow leaf curling virus NC_004324;

VSSTLEEVCDENPLDVKNTNTELVKIELINPEKEVNVPNNIPYSLRDINEFSQECADLVRKGIIEESKSPHSAPAFYVENHNEIKRKKRRMVINYKALNKATIGNAHKLPRIDSILTKVKGSNWFSTLDAKSGYWQLRLHPQSKPLTAFSCPPQKHYQWNVLPFGLKQAPGIYQNFMDKNLEGLENFCLAYIDDILVFTNSSREEHLSKLLVVLERCKEKGLILSKKKAIIARQTIDFLGLTLQENGEIKLQPNVLEKLELFPDAIEDRKQLQRFLGCLNYIADKGFLKEIAKETKNLYPKVSITNPWHW

>Soymovirus04; Peanut chlorotic streak virus NC_001634;

VTQLLEAICSQNPLDPQKNRNQIIVHIDLIDPTKEVNVPNRIPYTQKDIDEFREETSKQIELGILRQSKSPHSAPAFYVENHNEIKRGKRRLVINYKMNKATKGDAYNLDRLYLTDRESNWFSTLDAKSGFLQLRLDEETKPLTAFSCPPQMHLEYNVMPMGLKQAPSQFQRFMDNNLRGLEDISLAYIDDIIVFTKGTKDYHLKQVARVLIQLGNHGVILSKEKAKIAFEEIEFLGLKILKNGFIEPQKHLLEKIAEFPDQLQDRKQIQKFLGCLNYIGEKGFFKELAKERKVLQKMLSEKLPWKW

>Soymovirus05; Soybean chlorotic mottle virus NC_001739;

IEEQLEEVCSEHPLDETKNKNGLLIEIRLKDPLQEINVTNRIPYTIRDVQEFKEECEDLLKKGLIRESQSPHSAPAFYVENHNEIKRGKRRMVINYKKMNEATIGDSYKLPRKDFILEKIKGSLWFSSLDAKSGYYQLRLHENTKPLTAFSCPPQKHYEWNVLSFGLKQAPSIYQRFMDQSLKGLEHICLAYIDDILIFTKGSKEQHVNDVRIVLQRIKEKGIIISKKKSKLIQQEIEYLGLKIQGNGEIDLSPHTQEKILQFPDELEDRKQIQRFLGCINYIANEGFFKNLALERKHLQKKISVKNPWKW

>Soymovirus06; Water chestnut soymovirus 1 KU365408;

VWNQIDEMSSDNPLDPVKNTNSELVEIKLKDPKQEVNVVNRIPYTEQDVLEFVEETSMMLEKGIIEIANSPHSAPAFYVNNHAEQKRGKRRMVINYKAMNNATVGEAYKLPRQDYLFERIKDADWFSSLDAKSGYWQLRLAENTKPLTAFSCPPQKHFQFKVMPFGLKQAPSIYQRFMDKTLKGLESNCLAYIDDVIIFTRGNKIEHLKVVSGILKQIKLAGLILSKKKCQIGKEEVQFLGMKILKKGKIKPQEHLLEKLQDFSDQLEDRKQIQKFLGCLNYLCDKGFIKNIVEKKKILQDLLSTKKLWKW

>Cavemovirus01; Sweet potato collusive virus HQ694978;

LNNILGEDIYGDNPLKHWEKHKTYAKIELKNPDDHIYKPPINYQESDYKEFKMHIDEMVKEGFIEECKNLENKKYSSPAFIVNKHSEIKRGKSRMVIDYKDLNKKAKVIKHPIPNKDILINRGIKANYFSKFDCKSGFYHIKLEEDSKKYTAFTVPQGYYVWIVLPFGYHNSPSIYQQFMDGIFRPYYDFILVYIDDILIFSKTYEEHKIHLEIFRNIIIKHGIVLSKKKAEIGKQKIEFLGVKIEQGGIELQPHIIDKILEKHIKIKSKKELQSILGLVNQIRNFLPNLSKILLPIQKKLKIKNEEVW

>Cavemovirus02; Cassava vein mosaic virus U59751;

INQILGTDIYGENPLEKWEKHKTLAKIELKNETDNIYKPPMLYQETDLPEFKMHIEEMIKEGFIEEKTNFEDKKYSSPAFIVNKHSEQKRGKTRMVIDYKDLNKKAKVVKYPIPNKDTLIHRSIQARYYSKFDCKSGFYHIKLEEDSKKYTAFTVPQGYYQWKVLPFGYHNSPSIFQQFMDRIFRPYYDFIIVYIDDILVFSKTIEEHKIHIAKFRDITLANGLIISKKKTELCKEKIDFLGVQIEQGGIELQPHIINKILEKHTKIKNKTELQSILGLLNQIRHFIPHLAQILLPIQKKLKIKDEEIW

>Cavemovirus03; Chicory mosaic cavemovirus MW811396;

LLQILGEEIFNENPLQGWEKHKTYAKIELKNPNDEIYKPPMIYKEEDYKEFELHITELIKGDYIEQKDKYENKKYSSPAFIVNNHSEQKRGKTRMVIDYKDLNKKAKIIKHPIPNKDILLHRGIKAKIFSKFDCKSGFYHIKLEEESKKYTAFTVPQGYYQWKVLPFGYHNAPSIFQKFMDIIFRRYYEFIIVYIDDILIFSNNINEHKYHLKIFKEIVKENGISLSKKKAELGKEKIEFLGMQIEQGGLKIQQHIIDKILTKHIKIKNKKELQSVLGLLNQIRNFIPRLAEILQPLQKKLKIKKEEYW

>Cavemovirus04; Epiphyllum virus 4 NC_055588;

INKVLGSDVFGEHPLQGWEKHKTFAKIELKNDDEEIYKPPLHYQPSDLPEFKMHIDEMLRDDYIEEKTNFENKKYSSPAFIVNKHSEQKRGKTRLVIDYKDLNKKAKIIKHPIPNKDILIIRGIKANYFSKFDCKSGFYHIKLDKESKKYTAFTVPQGYYQWKVLPFGYHNSPSIFQQFMDKIFRPYYEFILVYIDDILIFSHTEEDHLKHIKIFKECILQNGITLSNKKSELGKTTIHFLGVIITHGGIELQPHIIDKILEKHIKIKNKTELQSILGLLNQIRNFVPNLAKLLKPLQKRLKIKPEEQW

>Solendovirus01; Tobacco vein-clearing virus AF190123;

IQKKLEKLYNDNPLQGWEKHKTKVKIELIEENSIITQKPLKYNFNDLTEFKIHIKDLLDNKYIQESNSKHTSPAFIVNKHSEQKRGKSRMVIDYRNLNAKTKTYNYPIPNKILKIRQIQGYNYFSKFDCKSGFYHLKLEDESKKLTAFTVPQGFYEWNVLPFGYKNAPGRYQHFMDNYFNQLENCIVYIDDILLYSRTQDEHIKLLEKFAHIIENSGISLSKTKAEIMKNQIEFLGIQIDKNGIKMQTHIVQKIINLDENIDTKKKLQSFLGIVNQVREYIPKLAENLKPLQKKLKKDVEYSF

>Solendovirus02; Bacopa monnieri virus 3 BK014481;

LAHRLDDFYTDNPLTNWKKHKTICNIELINKDSIIQQKPFFNTEKDINEFKMHIDELIKMEFIQNSNSKHSSSAFIVNKHSEIKRGKSRMVIDYRILNAKTKTYNYPIPNKILKIKQIQGYKWFSKFDCKSGFHHIKLSEDSKELTAFSVPNGFYEWNVLPFGYKNAPGIFQSFMDKYFNQLENCVVYIDDILLFTKTEDEHIQLLKNFIEIVDKSGIVLSKKKTELFKNQIEFLGHDIDIAGIKMQSHIAIKLLEFTENIETRKQLQSFLGLINQLREFIPKLSKHLVPLQKKLKKDIQWSW

>Solendovirus03; Sweet potato vein clearing virus HQ694979;

VRNQIKTLFSENPLEFWDKHRTEVKIELINPDSIVYQKPLRWNFEDIEEFKLHIDELLKGGFIRPSNSKHSSPAFIVNKHSEQKRGKSRMVIDYRNLNAKTKTYNYPLPNKILRVRQVQGYNWFSKFDCKSGFYHLKLTEESKHLSAFNVPQGFYEFNVLMFGYKNAPGRYQCYMDSYFSKLENCIVYIDDILLYSKTKDEHETLLKKFYHIVKEAGVSLSEKKAIIGVNQIEFLGIEIDKSGVKMQNHIVTKIVQCEEVLDTKKKLQSFLGLINQVREYVPNIAKELLFLQKKLKKDVEYHF

>Dioscovirus01; Dioscorea nummularia-associated virus NC_040712;

LHKLEEIQIIGNDPLKYWEKNQIRCKLDIINPDLTIQDKPIIPSPEMAKEYEKHITELLALKVIRPSQSRHRTAAFIVNKHSEQVRGKSRMVYNYKRLNDNTYKDQYTLPSIDYLLLKIKDKIVYSKFDLKSGFHQIMMDPQSIEWTAFVCPQGHFEWIVMPFGLKNAPSVFQRKMDNIFKKYSEFVCVYIDDILIFSESIQQHVQHLLQFFQVCKEEGLILSKTKLKIGVANIEFLGLEIGEGKVQLQPHILKNILEFPEDQLETLKGLQKFLGILNYARNYIPNLSKYTRIFYNKCSSKGERKF

>Rosadnavirus01; Rose yellow vein virus JX028536;

VEEILLDTFSDNPLALYNPRHPKCQIELLPQDQLDRLKLKCPVRCKPIGANPIDMEEFHNQINELLRLKLIRKTNSPWSFQAFMVRNHAEIVRGKARMVINYKPLNLRIRKNAYRIPNKDSLFLAIRESQFYSKFDCKSGFYQVPMEQDSIQLTAFSTPIGSYEWLVMPFGLATAPSIFQAKMDNVLEDHHDYCLVYIDDIIVFSRTLEEHKIHVITIAKTLKKNGIVISKKKMELGLTKINFLGCEIENGRIILQNHVLENLSKFPSEIKDKKELQSFLGIINYAASHYSIEVTKLRVPLQKKLKKNYIWSW

>Tungrovirus01; Rice tungro bacilliform virus AF076470;

IKELEALGFIGDDITKNRTTWVCDFKIINPDINITCATIPYTPADKEIFEKQIKELLDNKLIKKASPTCRHRTAAFIVRNHAEEVAQKPRIVYNYKRLNDNMHTDPFNIPHKISMINLIQKANIFSKFDLKAGFHHMKLKEDFKDWTTFTCSEGLFTWNVCPFGIANAPCAFQRFMQESFGDLKFALLYIDDILIASSNEQEHIKHLKIFFNRVKEVGCVLSKRKSKMFLKEVEYLGVEIKEGKISLQPHIVEKIKRFDKSKLSTLKGLQAYLGLLNYARSYIKDLSKLVGPLYKKTGKSGQRSF

>Tungrovirus02; Agapanthus tungrovirus_MT501687;

IKDLETLGFLGDDITKCQSNWVCKFKVLNPDIKITCKGIDETPADKEEFRKQIAELLNQGLIYQAKPDCRHRSAAFIVRNHSEEKRGKARIVYNYKRLNDNMETDPFNLPHKNSLMNLIQGAKVFSKFDLKSGFHQMKLEEWFQPWTTFTCSEGLYTWKVCPFGIANAPCAFQRFMQDVFGHLKFCLVYIDDILVMSKSRDQHKKHLLEFFDTVKNNGIVLSKKKTELFKEEIDYLGLKINKGKIELQPHISQKILTFDKKTLESKKGLQSYLGLLNYARPFFKDLSKLIKPLYSKTGKNGQSYF

>Tungrovirus03; Piper DNA virus 1 JX406741;

MRELEKLGLIGEDLAKNNCQHEFSLNIKNPDLIIRTANLEYTPEDIEEFKKQIKELLDLKLIRRSTSPHRSAAFIVRNHAEQKRGKARIVYNYKRLNDNTHDDAYNLPHKDSILNLIQNKKIFSKFDLKSGYNQIKMKEEDRPWTAFTCPEGLFEWNVLSFGLKNAPAIFQRFMDSLFKKYEFCIVYIDDILVASDTVQEHIKHLELVFKTIKEAGIVISKKKTEIAKTYINFLGLKIGKGQIELQPHIVTKALEYPDKIENKNKLQSFLGLLNYARKFIPNLSKLVGPLYSKLRKNGQIYF

>Petuvirus01; Petunia vein clearing virus MN399814;

LCAESHVDFLSKCSHPLWLNQDFFIKLPFKKNENINPTKASHSGMNPEHLQLAIKECDELQQFDLIEPSDSQWACEAFYVNKRSEQVRGKLRLVINYQPLNHFLQDDKFPIPNKLTLFSHLSKAKLFSKFDLKSGFWQLGIHPNERPKTGFCIPDRHFQWKVMPFGLKTAPSLFQKAMIKIFQPILFSALVYIDDILLFSETLEDHIKLLNQFISLVKKFGVMLSAKKMILAQNKIQFLGMDFADGTFSPAGHISLELQKFPDTNLSVKQIQQFLGIVNYIRDFIPEVTEHISPLSDMLKKKPPAWG

>Petuvirus02; Citrus blight-associated pararetrovirus MN814438;

FCPESHSEFTHPNPLWKNKSFFIKLPFKLNEDINPTKAALPRMSPSDLLLAQKECSQLLVQGLIEPTSSQWACQAFYVEKHYEIVREKKRLVIDYQPLNMFLQDDKFPFPRRQSMFTFLKNAQIFSKFDLKSGFWQLGIEPSECYKTTFCIPNAHFQWTVLPFGLKTSSSIFQKSMVQIFQPILHHALIYIDDILLFSGSYNEHRQLLTQFYDIIQSHGIMLSAKKSTIATDNIEFLGMIIKDRHYQLGKHIAQELLHFPDQHLSKKQVQQLLGIINYIRDFIPHVDHYTHHLSALLKKKPPEWN

>Ruflodivirus01; Rudbeckia flower distortion virus FJ493469;

VNDLLTQACSENPLDENKNHNGLLAEIKLINPATTVNVKSMAYSPDDAVEINKQIQELLEIKVIRPSRSPHSSPCFLVQNHNEIKRGKKRLVINYKALNAATISDGYLLPNKETILTAIRGRKYFSTLDCKSGFWQIRLNENSKPLTAFSCPMGQYEWNVVPFGLKQAPGLFQRFMDNSFKEYSAFCAVYVDDILVFSKTLDEHYDHLETVLRKCIETGIILSKKKAEVAKTKINYLGFTISNGEIELQSHILENIKLFPSRIPDKKSLQRFLGILTYADQYIRKLAEWRKPLQRKLKKDTVWEW

>Florendovirus01; AtrichBV

LKNFIISEICNELPNAFWHRKQHIVGLPYEPNFNETKIPTKARPIQMNTELLQTCHHEIQDLLQKKLIRPSSSPWSCSAFYVNKNAEQERGVPRLVINYKPLNKVLQWIRYPIPNKKDLLDRLLHAVIFSKFDLKSGFWQIQIKEEDKYKTAFNVPFGQYEWNVMPFGLKNAPSEFQKIMNDIFNPFYKFMIVYIDDVLVFSESLEQHFKHLHTFIHTVKNAGLVVSAKKLNLFQTKIRFLGHNINQGTIIPIDRSILFADKFPDKILDKTELQRFLGCLNYISDFYQNLALDAKPLYDRLKKNPPPWT

>Florendovirus02; AtrichBV_sc1

IQHHIETSICSEHPNAFWERKKHNISLPYEQGFDEKQIPTKARPIQMNTILLDMCRKEITDLQNKKLIRPSSSPWSCAAFYVNKNAEKERGVPRLVINYKPLNKVLKWIRYPIPNKKDLLDRVAQACIFSKFDMKSGYWQIQIQEQDRYKTAFNVPFGQYEWNVMPFGLKNAPSEFQKIMNDVFNSYHQFMIVYIDDVLIFSESIDQHIKHINTFIHAVQNAGLVVSAKKITLFKTNIRFLGHMIHHGTIIPIDRSIQFADKFPDQILDKKELQRFLGCLNYVSDFYPNLAIDSKPLYDRLKKNPPPWT

>Florendovirus03; AtrichBV_sc2

LQQHIETSICSEHPNAFWERKKHSISLPYEQGFDEKQIPTKARPIQMNTILLDMCRKEIDDLQQKKLIRPSSSPWSCAAFYVNKNAEKERGVPRLVINYKPLNKVLKWIRYPIPNKKDLLDRVAQACIFSKFDMKSGYWQIQIQEQDKYKTAFNVPFGQYEWNVMPFGLKNAPSEFQKIMNDVFNAYHHFMIVYIDDVLIFSESIDQHIKHINTFIHAVRNAGLVVSAKKITLFKTNIRFLGHMIHHGTIIPIDRSIQFADKFPDQILDKKELQRFLGCLNYVSDFYPNLALDSKPLYDRLKKNPPPWT

>Florendovirus04; AalpV_sc1

IENDLLNQVCSDLPNAYWERKQHVVELPYIKEFNEKIIPTKTRPIQMNKKLLETCKQEINDLLQKKLIRPSKSPWSCSAFYVNNQAEKERGVPRLVINYKPLNEVLQWIRYPIPNKRDLLKRLYNAKIYSKFDMKSGFWQIQIAEQDKYKTAFTVPFGHYEWNVMPFGLKNAPSEFQHIMNDIFNNYSNFTIVYIDDVLVYSNSIDQHIAHLKTFINIVKKQGLVISAKKMHLFQTKIRFLGHNIYQGTITPIVRSIAFADKFPDELREKTQLQRFLGCLNYVSDFLPNLRKTIQPLFQRLQKIQNPGL

>Florendovirus05; CrubV

LETELLAQVCSELPNAFWERKKHIVEIPYEKDFNEKHIPTKARPIQMNQELLEHCKKEIQELLDKNLIRKSKSPWSCSAFYVNNQAEKERGVPRLVINYKPLNNVIQWIRYPIPNKRDLLKRLYEACIFSKFDMKSGFWQIQIAEKDKYKTAFTVPFGHYEWNVMPFGLKNAPSEFQNIMNEIFNPYTTFSIVYIDDVLIFSKSIDQHFKHLYTFLNIVKKHGLVVSAKKMQIFQTKIRFLGHNIYQGTITPISRSIEFVSKFPDEIKEKTQLQRFLGCLNYISDFLPNLRKTIQPLFQRLQKNPIPWS

>Florendovirus06; CclemV_scCc1

FKTLIEKEICADLPSAFWNRKQHMVDLPYETSFNERQIPTKARPIQMNMELEQHCKNEIKDLESKGLIVKSRSPWSCAAFYVNKNSEIERGTPRLVINYKPLNNALKWIRYPIPNKKDLLQKLCSALIFSKFDMKSGFWQIQIHPNDRYKTAFTVPFGQYEWTVMPFGLKNAPSEFQRIMNDIYNPYSDFCIVYIDDVLIFSRTIDQHFKHLKTFYFATKKAGLAISSSKISLFQTKVRFLGHHISKGTITPIERSLLFADKFPDKILDKTQLQRFLGSLNYVLDFCPNINRMSKPLHDRLKKNHVAWT

>Florendovirus07; CclemV_scCc2

FKDLIEKEICADLPSAFWNRKQHLVDLPYENSFDERQIPTKARPIQMNMDLEQHCRTEIKDLESKGLIVKSRSPWSCAAFYVNKNSEIERGVPRLVINYKPLNKALKWIRYPIPNKKDLLQKLHSAFIFSKFDMKSGFWQIQIHPKDRYKTAFTVPFGQYEWTVMPFGLKNAPSEFQRIMNDIYNPYSDFCIVYIDDVLIFSQTIDQHFKHLKTFYLATRKAGLAISKSKVSLFHTKIRFLGHHISKGTITPIERSLAFADKFPDKILDKTQLQRFLGSLNYVLDFCPNINRMSKPLHDRLKKNPVAWT

>Florendovirus08; CclemV_scCc3

FKNLIEKKICADLPSAFWNRKQHLVDLPYETSFNERQIPTKARPIQMNMELEQHCKNEIKDLESKGLIVKSRSPWSCAAFYVNKNSEIERGTPRLVINYKPLNKALKWIRYPIPNKKDLLQKLCSALIFSKFDMKSGFWQIQIHPKDRYKTAFTVPFGQYEWTVMPFGLKNAPSEFQRIMNDIYNPYSDFCIVYIDDVLIFSNSIDQHFKHLKTFYLATRKAGLAISSSKVSLFQTKVRFLGHHISKGTITPIERSLLFADKFPDKILDKTQLQRFLGSLNYVLDFCPNINRMSKPLHDRLKKNPVPWT

>Florendovirus09; CclemV_scCc4

FKDLIEKEICADLPSAFWNRKQHLVDLPYENSFDEKQIPTKARPIQMNMELEQHCKNEIKDLESKGLIVKSRSPWSCAAFYVNKNSEIERGTPRLVINYKPLNKALKWIRYPIPNKKDLLQKLHSAFIFSKFDMKSGFWQIQIHPKDRYKTAFTVPFGQYEWTVMPFGLKNAPSEFQRIMNDIYNPFSEFCIVYIDDVLIFFQTIDQHFKHLKTFYLATRKAGLAISSSKVSLFQTRIRFLGHYISKGTITPIERSLAFADKFPDKILDKTQLQRFLGSLNYVLDFCPNINRISKPLHDRLKKNPVAWT

>Florendovirus10; CclemV_scCc5

FKSLIEREICADLPSAFWDRKQHLVDLPYEKSFDEKQIPTKARPIQMNMELEQHCKDEINDLINKKLIVKSRSPWSCAAFYVNKNSEIERGVPRLVINYKPLNKALKWIRYPIPNKKDLLQKLCSAFIFSKFDMKSGFWQIQIHPKDRYKTAFTVPFGQYEWTVMPFGLKNAPSEFQRIMNDIYNPYSEFCIVYIDDVLIFSQSIDQHFKHLKTFHLVTKKAGLALSSTKISLFQTKVRFLGHHISKGTITPIERSLLFADKFPDKILDKTQLQRFLGSLNYVLDFCPNINRMSKPLHDRLKKNPVAWT

>Florendovirus11; CclemV_scCs1

FKNLIEKEICADLPSAFWNRKQHLVDLPYENSFDEKQIPTKARPIQMNMELEQHCKNEIKDLETKGLIVKSRSPWSCAAFYVNKNSEIERGTPRLVINYKPLNKALKWIRYPIPNKKDLLQKLYSAFIFSKFDMKSGFWQIQIHPKDRYKTAFTVPFGQYEWTVMPFGLKNAPSEFQRIMNDIYNPYSEFCIVYIDDVLIFSQTIDQHFKHLKTFYFATRKAGLAISSSKVSLFQTKIRFLGHYISKGTITPIERSLVFADKFPDKILDKTQLQRFLGSLNYVLDFCPNINRMSKPLHDRLKKNHVAWT

>Florendovirus12; CclemV_scPt1

FKTLIEKEICADLPSAFWNRKQHMVDLPYENSFDEKQIPTKARPIQMNMDLERHCKEEINDLVKKGLIVKSRSPWSCAAFYVNKNSEIERGTPRLVINYKPLNKALKWIRYPIPNKKDLLQKLHSAFIFSKFDMKSGFWQIQIHPKERYKTAFTVPFGQYEWTVMPFGLKNAPSEFQRIMNDIYNPYSDFCIVYIDDVLIFSNSIDQHFKHLKTFYFATRKAGLAISNSKVSLFQTKIRFLGHHISKGTITPIERSLAFADKFPDKILDKTQLQRFLGSLNYVLDFCPNISRLSKPLHDRLKKKPAAWT

>Florendovirus13; CsatBV

FQQRLEKEVCSNLPNAFWDRKKHMVTLPYKEGFKESQIPTKARPIQMNKDLVKVCTNEIKDLLKKGLISPSKSPWSCSTFYVNNQAEKERGVPRLVINYKPLNKVLKWIRYPIPNRQDLLKRITLAKVFSKFDMKSGFWQIQIHPTQHYKTTFNVPFGKFQWNVMPFGLKNAPSEFQKIMNDIFNPYQDFTIVYIDDILVFSNTVDQHFKHLRVFLNVIKTNGLVVSQPKIKLFQVKIRFLGYEINQGIIKPIQRSLDFADKFPDVIQDKTQLQRFLGCVNYIGDFIRDLRSICLPLYDRLKRNPKPWT

>Florendovirus14; EgranV_sc1

LQKHIEKTICSTLPNAFWERKKHIVDLPYEHDFSENKIPTKARPTQMNQEVLKFCQAEIQDLLNKKLIRKSQSPWSCSAFYVNKNAELERGVPRLVINYKPLNTALRWIRYPIPNKKDLLNRLCRSKIFSKFDMKSGFWQIQISEKDKYKTAFTVPFGQYEWNVMPFGLKNAPSEFQRIMNEIFNPYSEYIIVYIDDVLVFSSNIEQHFKHLSTFIKIIIQNGLVVSPTKIALFKTKIRFLGHYIHLSTITPIERSISFTDKFPDEIKDKTQLQRFLGSLNYILDFFPNINILCKPLHQRLRKDPPPWT

>Florendovirus15; FvesV_sc1

FEEKIIKEICSDIPTAFWHRKKHIVSLPYIKEFNEKNIPTKARPIQMSHEIMDFCKNEINDLLNKDIIRPSKSPWSCPAFYVQKNAELERGTPRLVINYKPLNTVLEWIRYPIPNKRDLINRLNKSVIFSKFDMKSGFWQIQISEADKYKTAFVTPFGHYEWNVMPFGLKNAPSEFQNIMNDIFNPYSHFSIVYIDDVLIFSQSIDQHWKHLHQFFSIIKNNGLVISASKIKLFQTNIRFLGFNIHQLQIKPIDRAIQFADKFPDVILDKNQLQRFLGSLNYVADFYKNLRKHCKPLFDRLQKNPSPWT

>Florendovirus16; GmaxV_sc1

LLNHIESTVCSELPHAFWDRKKHIVDLPYEKDFREKQIPTKARPIQMNEELLQYCQKEIKDLLDKGLIRKSKSPWSCAAFYVNKQSEIERGTPRLVINYKPLNQALQWIRYPIPNKKDLLNRLNSAKIFSKFDMKSGFWQIQIQESDRYKTAFTVPFGQYEWNVMPFGLKNAPSEFQKIMNDIFNPYSKFVIVYIDDVLIFSQNIDQHFKHLQTFIHIIKQNGLAVSKSKINLFQTRIRFLGHNIYQGTIIPIERSIEFASKFPNQILDKTQLQRFLGCLNYVGEFVPYLNNIVKPLHDRLKKNPPPWS

>Florendovirus17; GmaxV_sc2

LLSHIESTVCSELPHAFWDRKKHIVDLPYEKDFREKQIPTKARPIQMNEELLQYCQKEIKDLLDKGLIRKSKSPWSCAAFYVNKQSEIERGTPRLVINYKPLNQALQWIRYPIPNKKDLLNRLNSAKVFSKFDMKSGFWQIQIQESDRYKTAFTVPFGQYEWNVMPFGLKNAPSEFQKIMNDIFNPYSKFVIVYIDDVLIFSQDIDQHFKHLQTFIHIIKQNGLAVSKSKINLFQTRIRFLGHNIYQGTIIPIERSIEFASKFPNQILDKTQLQRFLGCLNYVGEFVPYLNNIVKPLHDRLKKNPPPWS

>Florendovirus18; GmaxV_sc3

LLKHIQSTICSDLPHTFWNQKKHIVDLPYEKDFREKQIPTKVRPIQMNEELLQYCQKEIKDLLDKGLIRKSKSPWSCVAFYVNKQAKLERGTPRLVINYKPLNQALQWIRYPIPNKKDLLNRLNSAKIFSKFDMKSGYWQIQIKETDRYKTAFTVPFGQYEWNVMPFGLKNAPSEFQKIMNDIFNPYSKFSIVYIDDVLIFSQTIEQHFKHLHTFINIIKQNGLAVSQTKINLFQTNIRFLGHNIHQGTIIPIDKSIEFANKFPNQILDKTQLQRFLGCLNYVGEFVPYLNNIVKQLHDRLRKNPPPWS

>Florendovirus19; GraimV

IKKEIESTICSDIPNAFWNRKKHEVTLPYENDFDERQIPTKARPIQMNKEMEEFCRKEIQDLLNKNLIRKSSSPWSCSAFYVIKNAELERGTPRLVINYKPLNQALKWIRYPIPNKKDLLQKLCNANIFSKFDMKSGFWQIQIKEEERYKTTFTVPFGQYEWNVMPFGLKNAPSEFQRIMNDIFYQYSQFTIVYIDDVLVFSENLEKHFKHISIFIKVIRNNGLVVSKSKISLFQTKVRFLGHYITQGTITPIERSIEFASKFPDQILDKVQLQRFLGSLNYVIDFYPGLSKLCKPLYDRLKKNPQPWT

>Florendovirus20; JcurV_sc1

LKESMEKELCADHPHAFWDRKQHIVDLPYEKDFDEKTIKTKARPIQMNPELIEHCKNEIQDLLNKKLISKSRSPWSCAAFYVNKNAEIERGVPRLVINYKPLNTALKWIRYPIPNKKDLLKNLYKANIFSKFDMKSGYWQIQIDPKDRYKTAFTVPFGQYEWNVMPFGLKNAPSEFQRIMNDIFNPYSKFCIVYIDDVLIFSTTLHEHFTHLKTFCMVIRQNGLALSKTKMDLFKTKIRFLGHYIEQGKIQPIERTLVFGEKFPDEITNKLQLQRFLGCLNYVIDFYPNLNCMLKLLHDRLRKNASPWT

>Florendovirus21; LjapAV

ILENIQSSICSDLPNAFWERKSHMVELPYEKDFSDKQIPTKARPIQMNEELLHFCQKEINDLLEKKLIRRSKSPWSCAAFYVNKQAEIERGTPRLVINYKPLNQALCWIRYPIPNKKDLLARLHDAKVFSKFDMKSGFWQIQLQEKDRYKTAFTVPFGQYEWNVMPFGLKNAPSEFQRIMNEIFNPYSKFTIVYIDDVLIFSQTLDQHFKHLNTFISVIKRNGLAVSKTKVSLFQTKIRFLGHNIHQGTIIPINRAIEFTDKFPDQIIDKTQLQRFLGCLNYVADFCPQLSTIIKPLHDRLKKDPPPWS

>Florendovirus22; LjapBV

LLKEIQSSICSDLPSAFWERKRHMVDLPYEKDFNDRKIPTKARPIQMNEKLLQFCQKEINDLLSKKLIRKRKSPWSCSTFYVNKQAEIERGTPRLVINYKPLNQALGWNRYHIPNKKDLLARLHDAKIFSKFDTKSEFWQIQIKEEDRYKTAFTVPFGQYEWNVMPFRLKNAPSKFQKIMNDIFNPYSKFTIVYIDDVPVFSQSIDQHFKHINIFISIIKKNGLAVSRSKISLFQTRIRFLGHNIHQGTIIPINRAIEFTDKFPDQIIDKTQLQRFLGCLNYVADFCPQISNLIKPLHDRLKKDPPPWS

>Florendovirus23; LjapCV_sc1

FQDRIEKDLCDLNPMAFHHRKIHTITLPYIDGFNEKDIPTKARPIQMNEQYLKYCREEIGEYLNKGLIRHSKSPWSCTGFYVMNASELERGAPRLVINYKPLNKVLKWIRYPLPNKTDLIKRLHKATIFSKFDMKSGYYQISVKEEDRYKTAFVVPFGHYEWNVMPQGLKNAPSEYQNIMNDIFYPYMDFTIVYLDDVLVFSKGIDQHVQHLEKFIEIIKKNGLVVSAKKMKIFETKTRFLGYEIHQGQITPIQRSLEFAKNFPNELKEKNQLQRFLGCVNYVADFIPNIRIICAPLFKRLRKNSPPWS

>Florendovirus24; LjapCV_sc2

FQDQIEKDLCDLNPMAFHHRKIHTIALPYIDGFNEKDIPTKARPIQMNEQYLKYCREEIGEYLNKGLIRHSKSPWSCTGFYVMNASELERGAPRLVINYKPLNKVLKWIRYPLPNKTDLIKRLHKATIFSKFDMKSGYYQISVREEDRYKTAFVVPFGHYEWNVMPQGLKNAPSEYQNIMNDIFYPYMDFTIVYLDDVLVFSKGIDQHVQHLEKFIEIIKKNGLVVSAKKMKIFETKTRFLGYEIHQGQITPIQRSLEFAKKFPDELKEKTQLQRFLGCVNYVADFIPNIRIICAPLFKRLRKNSPPWS

>Florendovirus25; MdomV_scMd1

FEEQLVKEVCSDIPNAFWHRKQHIVKLPYIKEFNESKIPTKARPIQMSQEVMDFCKAEIQDLLNKGIIRKSKSPWSCPAFYVQKSAELERGSPRLVINYKPLNEVLEWIRYPIPNKRDLIKRLSKATIFSKFDMKSGFWQIQIHESDKYKTAFVTPFGHYEWNVMPFGLKNAPSEFQNIMNEIFNQYSHFTIVYIDDVLVFSKSIDEHWKHLHLFARIIRSNGLVVSATKIKLFQLKVRFLGYNIHRSTIQPIDRVIQFADKFPDQITEKTQLQRFLGSLNYVSEFYPHLRQQCKPLFDRLKENPPAWS

>Florendovirus26; MdomV_scPp1

FSDKLTKEICSDLPNAFWHRKQHIVKLPYIKDFIESKIPTKARPIQMNRQVLEFCKTEINQLLDKGIIRKSKSPWSCPAFYIQKNAELERGVPRLVINYKPLNDVLEWIRYPIPNKKDLIKRLSQATVFSKFDMKSVFWQIQIHESDKYKTAFVTPFRHYEWNVMPFGLKNAPSEFQNIMNEIFNQYSHFSIVYIDDVLIFSKSINEHWKHLHAFARIIRSNGLVVSASKIKLFQTKVRFLGYHIYRSTIQPIDRVIQFADKFPDQIIDKTQLQRFLGSLNYVSEFYPHLRQQCKPLFDQLKENPPSWS

>Florendovirus27; MescV

IQEEMLNSICAESPDAFWTRKKHVVNLPYEPEFTEKAIPTKARPIAMGPRHLEICKKEIAELEAKGLIRKSSSPWSCPAFYVENAAELERGVPRLVINYKPLNKALRWVRYPLPNKRDLLNRLYEATIFSKFDMKSGYWQIQIAEEDKYKTAFTVPFGHYEWNVMPFGLKNAPSEFQKIMNEIFNAYSAFSIVYIDDVLIFSKTIDQHFKHLKMFEKIVKLNGLVVSAKKIKIFQTEIRFLGHNIAKGTIIPINRAIEFASKFPDEIKEKTQLQRFLGSLNYVADFYKNLAQDAKPLFQRLKKNPPEWT

>Florendovirus28; MgutV_sc1

IQEHIVQTICSDIPNAFWERKKHIVHLPYEPDFKESQIPTKARPIAMGPEYLEMCKKEIEDLLKKGLIRKSYSPWSCPAFYVNKNAELERGVPRLVINYKPLNKALRWIRYPIPNKRDLLNRLYTAKIMSKFDMKSGFWQIQIAEEDRYKTAFTVPFGHYEWNVMPFGLKNAPSEFQNIMNDIFNPFTHFCIVYIDDVLIFSESIDQHIKHLKAFINATTKAGLVVSAKKIKLFQTETRFLGHNIKEGTIIPIERSITFAEKFPDEIKDKNQLQRFLGSLNYIADFYTNLAFDTKPLFERLKKNPPEWT

>Florendovirus29; NbenV_sc1

LQQKFEREVCSNLPTAFWNIKRHSVALPYIDDFDERSIPTKARPIQMNQEMLETCKTEINHLLENGIIRPSNSPWSCSAFYVNKSAEKERGAPRLVINYKPLNSVLKWIRHPIPNKRDLLKRTYKANLYSKFDMKSGFWQIQIKNEDRYKTAFNVPFGHYEWNVMPFGLKNAPSEFQNIMNNIFNPYSYMSIVYIDDVLIFSEDIDSHFKHLNTFFNVVKHNGLVVSAKKIKLFQTTIRFLGHDLYQGSYKPICRAIEFSDKFPNEIKDKTQLQRFLGSLNYVADFIPNIRQVCEPLYKRLRKNPSPWS

>Florendovirus30; NbenV_sc2

LQRKFENEVCSNLPSAFWNVKKHSVALPYIDGFDERNIPTKARPIQMNQEMLEVCKNEINHLLDNGIIRPSNSPWSCSAFYVNNSAEKERGAPRLVINYKPLNSVLKWIRHPIPNKRDLLKRTYKANLYSKFDMKSGFWQIQIKDEDRYKTAFNVPFGHYEWNVMPFGLKNAPSEFQNIMNSIFNPYSYMSIVYIDDVLIFSEDIDSHFKHLNTFFNVVKNNGLVVSAKKIKLFQTSIRFLGHDLYQGTHRPICRAIEFSSKFPDEIKDKTQLQRFLGSLNYVADFIPKIRQVCEPLYHRLRKNPAPWG

>Florendovirus31; OsatBV_compAsc1

LEKRFIKEICSDFPSAFWHRKKHVVGLPYISNFNEDKIPTKARPIQMNSRLLEICKQEIKNLLDKGLIRKSSSPWSCAAFYVENAAEKEQGVPRLVINYKPLNKVLQWIRYPIPYKHDLIRRIQGSKIYSKFDMKSGFWQIQIKEEDRYKTAFTTPFGHYEWNVMPFGLKNAPSEFQKIMNEIFLPFTSFIIVYIDDVLIFSQDVDQHWKHLNIFYKIIVQNGLVVSAKKMKLFQTNVQFLGYKIQYDQVQPVARVIEFAEKFPDEIKDKTQLQRFLGCLNYVSDFYKDLAKDRKILTERLKKKPPAWT

>Florendovirus32; PpersV_sc1

FEQRLKEEICSELPTAFWYRKQHVVRLPYIKTFSEKNIPTKARPIQMSQEMMEFCKKEIEELLQKKIIRKSKSPWSCPAFYVQKNAELERGVPRLVINYKPLNAVLEWIRYPIPNKRDLINRLEKAVVYSKFDMKSGFWQIQIDESDRYKTAFVTPFGHYEWNVMPFGLKNAPSEFQNIMNDIFNPYSQFSIVYIDDVLIFSESIEQHWKHLNKFLQVVKQNGLVVSAKKIKLFQTNIRFLGFNICQSQISPIDRVIQFADKFPDQILDKSQLQRFLGSLNYISDFYQNLRKQCKPLFDRLQSNPPPWS

>Florendovirus33; PtrichV_sc1

LQNKIEQQICSDLPNAFWKRKQHIVDLPYEDTFSEKLIPTKARPIQMNADLEQHCRLEIHDLESKGLIQKSRSPWSCAAFYVNKNSEIERGTPRLVINYKPLNSALKWIRYPIPNKKDLLQKLHSAFIFSKFDMKSGFWQIQIDPKDRYKTAFTVPFGQYEWNVMPFGLKNAPSEFQRIMNDIFNAHSKFCIVYIDDVLIFSHSIDQHFKHLHTFFHTAKQNGLVVSKTKISLFQTRVRFLGHYICQGTVTPIERSLTFTNKFPDKITDKTQLQRFLGSLNYVLDYYPNISRLAKPLHDRLKTNPIPWS

>Florendovirus34; RcomV_sc1

LQKKIENEVCSDLPNAFWRRKQHMVDLPYEKDFSDKQIPTKARPIQMNETLESHCRIEIKDLEQKGLITKSRSPWSCAAFYVNKNSEIERGTPRLVINYKPLNKALKWIRYPIPNKKDLLQKLHSAFIFSKFDMKSGFWQIQIHPKDRYKTAFTVPFGQYEWNVMPFGLKNAPSEFQKIMNDIFNPYSKFCIVYIDDVLIFSNSLEQHFKHLETFFYVVKKNGLVVSKSKISLFQTKIRFLGHYISRGTITPIERSLAFTSKFPDKILEKTQLQRFLGSLNYVMDFYPNLNCLAKPLHDRLKKNPPAWT

>Florendovirus35; RcomV_sc2

FQMKIENELCSDLPNAFWNRKQHMVDLPYENDFSDKQIPTKARPIQMNESLESHCRLEIKDLESKGLISKSRSPWSCAAFYVNKNSEIERGTPRLVINYKPLNKALKWIRYPIPNKKDLLQKLHSAFIFSKFDMKSGFWQIQIDPKDRYKTAFTVPFGQYEWNVMPFGLKNAPSEFQKIMNDIFNPFSKFCIVYIDDVLIFSNSIEQHFKHLETFFYVVKKNGLVVSKSKISLFQTKIRFLGHYISRGTITPIERSLAFTSKFPDKILEKTQLQRFLGSLNYVMDFYPNLNCLAKPLHDRLRKNPPPWS

>Florendovirus36; StubV_scSt1

LQKKFEEEICSDFPNAFWERKKHIVDLPYIEGFNEQAITTKARPIQMNHEMMEFCKKEIDTLLKNKIIRISKSPWSCSAFYVNKNAEKERGAPRLVINYKPLNSVLKWIRYPIPNKRDLLKRTFKANVYSKFDMKSGFWQIQISEKDKYKTAFNVPFGQYEWNVMPFGLKNAPSEFQNIMNSIFNDYSYMSIVYIDDVLIFSENIDSHFKHLNTFFKIIKNNGLVVSAKKMVLFQTTIRFLGHDLYQGTYKPICRAIEFSSKFPNEIPDKTQLQRFLGSLNYVADFIPKVRQVCEPLYKRLRKNPVPWS

>Florendovirus37; StubV_scSt2

LQKTFEEEICSDFPNAFWERKKHIVDLPYIEGFSEQAITTKARPIQMNHEMMEFCKKEIDTLLKNKIIRISKSPWSCFAFYVNKNAEKERGAPRLVINYKPLNSVLKWIRYPIPNKRDLLKRTFKANVYSKFDMKSGFWQIQISEKDKYKTAFNVPFGQYEWNVMPFGLKNAPSEFQNIMNSIFNNYSYMSIVYIDDVLIFSENIDSHFKHLNTFFKVVKHNGLVVSAKKMVLFQTTIRFLGHDLYQGTYKPICRAIEFSSKFPNEISDKTQLQRFLGSLNYVADFIPKVRHVCEPLYKRLRKNPVPWS

>Florendovirus38; StubV_scSt4

LQKKFEEEICSDFPNAFWERKKHIVDLPYIEGFNEQAITTKARPIQMNHEMMEFCKKEIDTLLKNKIIRISKSPWSCSAFYVNKNAEKERGAPRLVINYKPLNSVLKWIRYPIPNKRDLLKRTFKANIYSKFDMKSGFWQIQISEKDKYKTAFNVPFGQYEWNVMPFGLKNAPSEFQNIMNSIFNDYSYMSIVYIDDVLIFSENIDSHFKHLNTFFKIIKNNGLVVSAKKMVLFQTTIRFLGHDLYQGTYKPICRAIEFSSKFPNEIPDKTQLQRFLGSLNYVADFIPKVRQTCEPLYKRLRKNPIPWS

>Florendovirus39; StubV_scSl1

LREKFEKEICSDFPNAFWNRKKHIVSLPYIEGFNERAITTKARPIQMNHEMMEYCKEEINTLLKNGIIRVSKSPWSCSAFYVNKNSEKERGAPRLVINYKPLNSVLKWIRYPIPNKRDLLKRIFNAKIFSKFDMKSGFWQIQISEKDKYKTAFNVPFGQFEWNVMPFGLKNAPSEFQNIMNSIFNDYSYMSIVYIDDVLIFSENIDSHFKHLNTFFNIVKNNGLVVSAKKMILFQTKIRFLGHDLFQGTFKPICRALEFSSKFPNEIIEKTQLQRFLGSLNYVADFIPKIKHICEPLYKRLKKVPVPWS

>Florendovirus40; SbicV_compAsc1

LEDKFIKDICSDIPSAFWHRKKHVVTLPYISGFSEDKIPTKARPIQMNSRLLEICKSEINELLKKGLIRKSSAPWSCAAFYVENAAEKERGVPRLVINYKPLNKVLQWIRYPIPYKHDLIRRIQGSQIYSKFDMKSGFWQIQIKEEDRYKTAFTTPFGHYEWNVMPFGLKNAPSEFQKIMNEIFLPYTSFIIVYIDDVLIFSQNIDQHWKHLNIFHKIIIQNGLVVSARKMKLFQTNIQFLGYKIQHDQVLPVTRVIEFADKFPDEIKEKKQLQRFLGCLNYVSDFYERLAKDRKILTERLKKNPPAWT

>Florendovirus41; TcacV_sc1

IKKEIEDTICSDIPNAFWDRKKHQVELPYTKDFNEKQIPTKARAIQMNKEMEEFCKKEIKDLLDKKLIRKSNSPWSCSAFYVIKNAEIERGTPRLVINYKPLNKALEWIRYPIPTKKDLLQKLCNAKIFSKFDMKSGFWQIQIKETERYKTAFTVPFRQYEWNVMPFGLKNAPSEFQRIMNEIFNPYSQFTIVYIDDVLIFSQNLQKHFKHLRIFINIIKKNGLVVSKSKTSLFQTKIRFLGHYVTQGTIIPIERSIKFANNLPDKIIEKTKLQRFLGNLNYVIDFYPELNKICKPLHDKLKKNPSPWT

>Florendovirus42; VvinAV_sc1

FKDKIVGEVCSNIPNAFWHRKQHEVELPYEPDFSEKNIPTKARPIQMNKDLLSYCEKEIQDLLDKKLIRKSKSPWSCSAFYVQKQAELERGTPRLVINYKPLNDALRWIRYPIPNKKDLLQRLVKAKVFSKFDMKSGFWQIQIAEKDRYKTTFVVPFGHYEWNVMPFGLKNAPSEFQNIMNEIFNQFSDFIIVYIDDVLVYSTSIEEHWKHLNKFIENVKSNKLSLSTTKINLFHTRIRFLGHHIHQGTITPIQRSIEFTDKFPDEIKHKKQLQRFLGSINYVSNFIQDLSQLCAPLRQKLKKNLVPWN

>Florendovirus43; VvinBV_compAsc1

LKNKIIKEVCSNIPNAFWHRKQHEVELPYEPDFSEKNIPTKARPIQMNKDLLSYCEKEIQDLMDKKLIRKSKSPWSCSAFYVQKQAELERGTPRLVINYKPLNDVLRWIRYPIPNKKDLLQRLGKSKVFSKFDMKSGFWQIQIAEKDRYKTAFVVPFGHYEWNVMPFGLKNAPSEFQNIMNEIFNQFSDFIIVYIDDVLIYSDSVEQHWKHLNRFIETVKSNGLSLSATKINLFQTKVRFLGHHIHQGTFTPIQRSIEFADKFPDEIKDKKQLQRFLGSLNYVSDFIQDLSQLCAPLRQRLKKNPVPWN

>Florendovirus44; VvinBV_compAsc2

LKNKIIKEVCSNIPNAFWHRKQHEVELPYEPDFSEKNIPTKARPIQMNKDLLSYCEKEIQDLMDKKLIRKSKSPWSCSAFYVQKQAELERGTPRLVINYKPLNDVLRWIRYPIPNKKDLLQRLGKSKVFSKFDMKSGFWQIQIAEKDRYKTAFVVPFGHYEWNVMPFGLKNAPSEFQNIMNEIFNQFSDFIIVYIDDVLIYSDSVEQHWKHLNRFIETVKNNGLSLSATKINLFQTKVRFLGHHIHQGTFTPIQRSIEFADKFPDEIKDKKQLQRFLGSLNYVSDFIQDLSQLCAPLRQRLKKNPVPWN

>Florendovirus45; VvinCV_sc1

IKSQIETDLCSSIPNAFWNRKKHKVSLPYVEGFDESQIPTKARPIQMNAQLLEYCKEEIKDLMNKNLIRKSQSPWSCAAFYVKKPSEIERGAPRLVINYKPLNKVLKWIRYPIPNKRDLIGRLYNASIFSKFDMKSGFWQIQLHEKDRYKTTFTVPFGHYEWNVMPFGLKNAPSEFQNIMNDIFNPYTNFSLVYIDDVLIFSNSLEQHFKHLETFQKIVRDNGLVISAPKIKLFQTKIRFLGFEIYQGTIKPIQRSIEFGSKFPDEIKDKTQLQRFLGSLNYVSDFYPNLRTTIKPLFARLRKTPKPWT

>Florendovirus46; VvinDV_compAsc1

IQDLFQNEICSDLPNAFWSRKKHEISLPYIQNFDESKIPTKARPIQMNEKLLEYCKQEIDSLLKKKLIRPSKSPWSCAAFYVQNAAEIERGAPRLVINYKPLNKVLQWIRYPIPNKQDLLKRLHSSVIYSKFDMKSGFWQVQIREEDRYKTAFTVPFGHYEWNVMPFGLKNAPSEFQNIMNDIFNPHFQFIIVYIDDVLVFSDSLEKHFIHLKKFFNVIKANGMACSAPKMKLFQTKIRFLGHEIFQGKTKPIQRSIEFADKFPDEIKDKKQLQRFLGCLNYVSDYFKDLRIICEPLYKRLRKNAPAWT

>Florendovirus47; VvinDV_compAsc2

IQDLFQKEICSDLPNAFWSRKKHEISLPYIQNFDESKIPTKARPIQMNEKLLEYCKQEIDSLIKKKLIRPSKSPWSCAAFYVQNVVELERGAPRLVINYKPLNKVLQWIRYPIPNKQDLLKRLHSAVIYSKFDMKSGFWQVQIKEEDRYKTAFTVPFGHYEWNVMPFGLKNAPSEFQNIMNDIFNPHFQFIIVYIDDVLVFSDSLEKHFVHLKKFFNVIKANGMTCSTPKMKLFQTKIRFLGHEIFQGKTKPIQRSIEFADKFPDEIKDKKQLQRFLGCLNYVSDYFKDLRIICEPLYKRLRKNAPAWT

>Xendovirus; -Gossypium raimondii; Issa Diop et al., 2018

LFKILENNFSEEPLNLWQNSPRYCEIKLENLNKIIRVKPMIYTKQDIDEFDIQIKELLKNYLIEKTNSPHSSPAFMVGNRAEIKREKARMVINYKRLNKILFFYGYFIPRKDVLINQAKQTKYFSKFDCKSRFWQIMLTEESKSLTAFSAPNGHYQWRVMPFGLCNAPQIFQKWMDSIFNKFKKNCVVYIDDILIFSDTLEKHRKHLNIISQEFIKHGIILSPKKIELEKTEIFLGLKLSADGLKLQDHIIVKIKEFPENIEDKKQLQQFLGIINYGRNLFSNLSEKIGKLYEKLKK

>Yendovirus; -Capiscum annuum; Issa Diop et al., 2018

IQILDKLVIIREKPLQHWDSNQITCKLEIINPECTIKTTSIEAKNEDLKDFEIQIKELLEGIIRRATSRHRSTTFIVKNHSELVREKARMVINYKRLNDNTVMDGCKLPDKIELINKIQGRKVFSKFDCKSGYQIKMHEDSIETAFTCPEGHFEWLVMPFGLKTACPIFQRKMNSIFRDYKKFVLVYVDDILVFSNNMRKNLGHLQVFKLFVDNGIIISRKKMELFKNSINFLGVVIGDSRIKLQPHIAKKVLEMPDKFEKTKDLQNFLGLLNYARAFIKDLGKVAGPLYSKTGSTFQKSF

>Zendovirus; -Fragaria vesca; Issa Diop et al., 2018

IERLLKPVISEDPQLYWERDPIYCQLKMHDPSAMLRPSLINYRKKDRKEMENQIQELLEKKLIRHSNSPHHAPAFLVRNHAEQLRGKARMVLITEMIKRPLKTVIRLMEFSSTGLKEQKSSLSLMSQAFGRLKCILIPYTFGTPQGHYEWLVMPFGLKQASSIFQRKMDNIFKPYSDFCIVYIDDILVFSKTMNKHLKHLEQVCKLIVQKGIILGQNKIHLIKGEIDFLGIHVKDGEIRLQDHIVKKISQFPDIILDAKSLRFLGVVNFARDFIPQVSGLTAFLSPKTSSKKKWSF

>Gymnendovirus1Pinus; Pinus taeda; Issa Diop et al., 2018

ISDKLHQDCTTSRPDAFWTREKYFVALPYKEGYVPKAQKASANHMSPTEQALCKQEIQQLLEQNLIEPCKSPWACPAFYVNKHSEQQRGKKRLVINYKILNEALMPIRYPLPNKELLLAKIANANVFSKFDLKSGFWQIGILPEDGYKTAFTVPHGQYQWTMMSFGLKNAPSEFQKRMEDIFGGVEYVIVYIDDLLVFSKDVNTHKMHLENFYEMVYKHGLVLSDSEEKFQIGKVKIDYLGLHIEQGHIELQPHVLLHLLKFPDILLDEKMLQRLLGCLNYIRQFYEKQSEDTRILQKRLRKKIGWS

>Gymnendovirus1Picea; Picea glauca; Issa Diop et al., 2018

IKEKIESDCTSDYPNAFWTREKYFVSLPYKEDYIPKPQKASANHMSPTECEYCQKEITELLERKLIEISRSPWACPAFYVNKHSEQKRGKPRMVINYRALNDALLPIRFPLPSKELLFSKIGKCNVFSKFDLKSGFWQIGIIPKDRYKTAFVVPNGQYQWKVMPFGLKNAPSEFQKRMDDIFKHLNFVIVYIDDLLVCSVDCKAHVQHLKVVYDLLYKHGLVLSRSKLCWAQTKIEYLGLILSKGEVELQDHILKKLSEFPDEILDQKQLQRFLGCLNYIRQFYENQAKDVRILQKRLSKVIPWN

>Gymnendovirus2Pinustaeda; Pinus taeda; Issa Diop et al., 2018

IYQRLLKGCSENPQQFWVIESPMQKIVTLHDNGVKGKMIPCTPVDEQEIRNQIKELLQMQLIEPSESHYSCSAFLVRNHSEVVRGKPRMVINYKPLNAITQGFNYPLPRSKMIMQKIQHSKVFSKFDMKSGYYQIQIQPEDRHKTAFICPAGFYQWKVVPFGLKNAPAFFQRRMDYIFAKYDFIVTYIDDILVHSPDIHTHLEHLETFLKEVQQHGIVLSEKKMSLFQDNIDFLGINVANGAIQMQPHVLTKLTQFPDELKDTKTIQRFLGVLNDLHKYIPHLSEKTTPIRKHEKQGWSQEA

>Gymnendovirus2Picea; Picea glauca; Issa Diop et al., 2018

ILEKLLKYCSSDPQAFWDVASPSMEIETLHDNGVTGKLIPYTPADEQEIKNQIEELMAMKLIEPSNSHYCCSAFIVRNHAEVVRGKARLVVNYKPLNAITKDFHYPLPRQEVLMQKLQNSKMFSKFDMKSGYYQVQIRPEDRHKTTFTCPAGLFQWKVVPFGLKNAPSYFQRRMDSIFNKYDFVIVYIDDILVHSQNEEDHVQHIAVFTSECEKHGIVLSDKKIELFRTTMEFLGICIIDGKIQMQPHIVQKIVGFPDQLENKNQIQNFWGILNYVHKYIPRLAEKTVAIRQHLSGGWSPTA

>Gymnendovirus2Pinusnigra; Pinus nigra virus 1 MH551472

IYQRLLKSCSDNPQQFWETESPMQEIVTLHDNGVKGKMIPCTPADEQEMRNQIQELLKMQLIEPSESHYACSAFLVRNHSEIVRGKPRMVINYKPLNAITQNFNYPLPRPEVIMQKIQHSKVFSKFDMKSGYYQIQIQPEDRHKTAFICPAGFYQWKVVPFGLKNAPAFFQRRMDYIFAKYDFIVTYIDDILIHSPDVQNHLKHLEIFLEEVKKHGIVLSERKMSLFQDNIDFLGINVANGSIQMQPHVLTKLTQFPDKLKDKKEIQRFIGVLNYLHKYIPNLSEKTAPIRRHNNGGWSDEA

>Gymnendovirus3; Pinus taeda; Issa Diop et al., 2018

LTSKFKECCLDDLNAFWHIKKQEVNLPLKKDFNGKLRRSKVVAMNEDQMKLCRAEIKDLLRKGLIRKSKSPIACFAFYVNKHAEIARGKPRLVVNYKPLNDILDYDAYPLPKRSVILAQISKSKIFSKFDLKSGFWQVGIKEEDKWKTAFSVPEGHYEWNIMPFGLINAPSAFQRIMDETFEGMEEFLKKYIDDLLIHSDNITDHIKHLKIFLYRVKEMGIVLSESKMKLLRPMIDFLGYNIQYGSYTVIQRSLNFFNHFPDEIKDKTQLQRFLESLNYISKFIKRCAQERKLLNKRLQKNLIPWN

>Gymnendovirus4; Pinus taeda; Issa Diop et al., 2018

LKEKLAKKCCSEEPNALWHKKQHTIKLPYKEGYKGKPSKSKAIPMSQEYRQLCQQEIQQLLNRGLIRESTSPNCYGFYVNKRPEQIRSIPRLVINYKPLNLVLADDTYPIPHKGDLIRRIAGAKIFSKFDKPGFWQVAVDEEDKFKTAFSIPAGHYENVMPFGLKNAPSKFQKVMDDIFKPYFDWLLVYIDDVLIFSKNLDDHFKHVNIFMKLVQKNGLVLSKKKMELFQTSIKFLGHQISNGQISLQQHAIEFADKFPDVLRDKTQLQRFLGCLNYVSSFYQDCATDRKVLNKRTGKNPPPWS

>Gymnendovirus5; Picea glauca; Issa Diop et al., 2018

LKEEFIKTVCSENPTAFWKQKKHEVYLPYKEDYQGRPCKSRAIPMNAEYQKLCAEEIESLLKKDLIRESTSPWNCYGFYVNKHSEQIRGVPRLVVNYKPLNKVLADDTYPIPNKSNLVTRIAGAKIFSKFDLKSGFWQVAIHEKDKFKTAFNVPAGHYEWNVMPFGLKNAPAKFQRVMDDTLKPYFDWLIVYIDDILVFSSSLDQHFKHLKILLQVIKQAGLVLSKKKIELFQTQVKFLGHTIKNGQITLQTHAVEFADKFPDKILDKTQLQRFLGSLNYISHFYKKCAQDRKLLNDRLKKEPTPWT

>Gymnendovirus6; Gingko biloba; Issa Diop et al., 2018

ITKQLEECCSMNPLAFWDKDQPEAEIELTDQNAKVYEKPIPCPEKYRKEMESQLEELLKLDLIEESKSPFCCPAFMVYKHSEIKRGKGRMVINYKRLNSVTKSFNYPLPHKETLFERIAGCQYFSKFDCKSGFYQIMVKAEDRWKTAFSALNGQFQWKVMPFGLKNAPAYFQRRMDKIFRHLDWIFVYIDDMLIFSRTLEDHVQHLKEFYEICKNHGIVLSSTKMVICKKEMEFLGFNIQGGKIKLQGHVLEKIDQFPDKMEDKKLITKIFRMFKLYWEFLFFGCQRPARITEFVKKGCSILD

>Fernendovirus1Bot; BEGM-2004510-Botrypus virginianus; Issa Diop et al., 2018

KSPLYSLLTLTTFWNFWKKQNIITLSSLPMETEIRASHSGMKLADAALCRQEITELLEKKLIEPSSSKWTGQAFYVNNRFKNQGEKIGNKFQATQASHTKRASYPIKGSAQKNPSRTDLFKVDFKLGFWQFMIHPDHKYKTTFIVPQGQYQWTVMPFGLRTAPSDFQAQIDHIFLPLQDCVIAYIDDILIFSKTIPVHINDLERFFNLIKIEGLVLSRSKCEFFKYEVKFLGITICNGTIKLHMFSKNCRTFPKIFLTKRPFNNFWDVIGYLTIPIWRKKPQFSLLCEKIISSGDLSR

>Fernendovirus1Lyc; ENQF-2084799-Lycopodium annotinum; Issa Diop et al., 2018

FTELFKTQSTMNPLEFWDKEQYMVTLPEVPLVNPTKATYMYMSPEDRILCEAEIAELLKMKLIEPSTSNWACQAFYVNKHSEQRRGKKRMVINYKPLNKYLVSVKYPLPLKDNLIDRIQGATVFSKFDLKSGFWQFKIHPKDRHKTAFTVPQGMYQWIVMPFGLKNAPSEFQKRIDLILKPVAKFTIGYIDDILVFSKSFALHDDHLTQVHNLFWNHGTALSEAKTVLFQPEIQFLGVTICQGKIKLQPHVLAKIAAMPATFSTLQEVQKFLGCLNWIHWNIPYLAELTRPLCKLLRKQQQPHQ

>Fernendovirus2Lin; NOKI-2097008-Lindsaea linearis; Issa Diop et al., 2018

VSTKMLTYTSDHPLQFWNHTQHLITLPMVPDANPTKAGHIGMIPEDLHLCQQEIRELLTKALIRPSNSPWASPAFYVNKHSEKRGCKRLVINYKPLTKYLICPKFPLPLKDLLLPQKIAGKTIFSKFDLKSGFWQFKIHHNHCYKTSFIISFGQYEWLVLPFGLNLAPSLCQEKVSQIFITLDQCCIVYMDDILIYSKTIAAHLLHLEAFYVLCKSGLVLSITKEICKSQIQFLGVILCNNTIKCEHVPKKIMEFPPQLTDVSQLCSLFGCCNSIWQYPFTILELSPFHAYLKKVSSLQW

>Fernendovirus2Dip; 1319 Dipteris conjugata; Issa Diop et al., 2018

MEQQLITNCSSTSPQAFKHIFHHQIGLPIKEGSKPYKESPYGFTHEDRLLCTAEITDLLQQGLIRESKSAWAAAAFYVNKYSEQKRGKKRLVINYKPLNKCLINIAYPLPNKEYLMQKISGATVFSKLDLKAGFWQIGLFPEDQHKTGFVVPQGFYEWTVLPFGLKTAPVEFQKFMDSIFSSIHEFCLVYIDDILIYSKDQHQHLQHLQRVHDLLQQHGLVLSPTKVQVGKASIDFLGVIIQHGAIQLQDHVLKALAAFPTEIHDKQQLQRFLGCLNYISPFYQDLAKDRYLLQLCLRKDGPKWG

>HIV-1-Retroviridae

PVNIIGRNMLTQIGCTLNFPISPIDTVPVKLKPGMDGPKVKQWPLTEEKIKALTEICKEMEEEGKISKIGPENPYNTPVFAIKKKDSTKWRKLVDFRELNKRTQDFWEVQLGIPHPAGLKKKKSVTVLDVGDAYFSVPLDESFRKYTAFTIPSINNETPGIRYQYNVLPQGWKGSPAIFQSSMTKILEPFRIKNPEIVIYQYMDDLYVGSDLEIGQHRTKIEELRAHLLSWGFTTPDKKHQKEPPFLWMGYELHPDRWTVQPIDLPEKDSWTVNDIQKLVGKLNWASQIYAGIKVKQLCKLLRGAKALTDIV

>c0007-RT_gypsy_Athila

VPKKGGITVAKNNNNDLIPTRTVTDWHVCIDYRKPNTATRKDHFPLSFIDQMLERLAGHAYYCFLYGYSGYNQIPIAPEDQEKTTFTCPFGTFSCRMPFGLCNTPATFQRCMMSIFSDMVERFIEVFMDNFSIFGSSFDYCLSNLALVLQRCKETNLVLNWEKCHFMVREGIVLGHKISTKGIEVDKAKIDTIANLPPPTSM

>c0091-RT_gypsy_Athila

VPKKSGITVVKNEDNELVPQRIQTGWRVCIDYRKLNTTTRKDHFPLPFIDQMLERLAGHSHYCFLDGYSGYNQIAIAPEDQEKTTFTCPFGTFAYRRMPFGLCNAPATFQRCMMSIFSDMVEEIIEVFMDDFSVFGDSFDICLHNLSLVLKRCQECNLVLNWEKCHFMVQQGIVLGHIISCRGIE

>c0031-RT_gypsy_CRM

TPKKDGSWRMCVDSRAVNKITVKYRFPIPRLEDMLDDLAGSQWFSKIDLRSGYHQIRIREGDEWKTAFKTPDGLYEWLVMPFGMSNAPSTFMRVMTHVLRPYIGKFLVVYFDDILIYSRSREEHLQHLRTIFSTLQKEKLYANLKKCSFLQPEVLFLGFNI

>c0078-RT_gypsy_CRM

VPKKYGSWRMCIDFRALNKITIKNRYPLLRIDDLMDQLQSARWFTKLDLKSGYHQVRIKEEDTWKTAFKTKQGLFEWLVMPFGLCNAPATFMRLMNEVLRSYIDDFVIVYLDDILVFSPTWEEHLIHVEKVLGTLRQYQLRLNLKKCEFGKSSLVYLGFIV

>c0085-RT_gypsy_Galadriel

QKKREGTLRLCIDYRALNKVTIKNKYPIPLIADLFDQLGGAKYFTKLDLRSGYYQVRIAPGDESKTACVTRYGSYEFLVMPFGLTNAPATFCTLMNKVFHPFLDKFVVVYIDDIVVYSNSLEEHLEHLQKVFQVLRENQLYVKREKCSFVQEEVEFLGHKI

>c1737-RT_gypsy_Galadriel

QKKREGTLRLCIDYRALNKVTVKNKYPIPLIANLFDQLGXARYFTKLYVRSGYYQVRIVLGDELKTACVTRYGSYEF

>c0049-RT_gypsy_Ogre

VLKRKTGAVRICVDYRNLNEASPKDEYPMPMADMLVDGAAHNQMLSFMDGNAGYNQIMMAEQDIHKTAFMCPGHIGAFEYTVMPFGLRNAGATYQRAMNSIFHDMIGHSLEVYIDDVVIKSPEEGNHVASLRKAFLRMRQHKLKMNPKKCVFGVQAGNFLGFLV

>c0059-RT_gypsy_Ogre

WLANIIPVKKKTGVIRICTDYRDLNQACIKDEFPLSNMDILIDSTSRXGLLSFMDGFSGYNQIKMSPKDAEKTAFRTPYGNFYYTIMPFGLKNARATYQRAITTVFHDMMRKWIEDYVDDLVVKSKARESHQEVLRKVLERCRLYQLKMNPKMCPFGVSSGKFLGFQV

>c0098-RT_gypsy_Reina

VSKKEGTWRFCVDYRALNQVTVKDKFPIPVIDEMLDELNGAAWFSKLDLRSGYHQIRMWDADIPKTAFRTHEGHYEFLVMPFGLSNAPSTFQALMNDIFRPYLRKFVLVFFDDILVYSRTLNEHVHHLTTVFEVLRVAQLKVKASKCTFAQSTVDYLGHTI

>c0236-RT_gypsy_Reina

LLVNKKEGTWRFCIDYRALNQITIKDKFPIPVIDELLDELYGASYFSKLDLRPGYHQIRMKDEDIPKTAFRTHEGHYEFLVMPFGLTNAPSTFQALMNSIFRTYLRKFVLVFFDDILVYSNSFTDHLAHLQQIFELLRANQLQVKMSKCSFGQRSVDYLGHTI

>c0003-RT_gypsy_Retand

VAKKDKGLWRVCVDYTDLNKACPKDNFPLPRIDQLVDSTSGNQLLSFMDAYSGYNQIMMHEDDKAKTSFIIERGTYCYKVMPFGLKNAGATYQRLVNKIFKEQIGKTMEVYVDDMLVKAPERADHIKNLAEAFSLLRKYNMKLNPSKCTFGVSSGRFLGYLV

>c0005-RT_gypsy_Retand

VRKPRKGWRMCVDYTNLNRACPKDSFPLPRIDQLVDATAGHALLSFMDAYSGYNQIFMHPEDQAHTSFITDRGLYCYKVMPFGLKNAGATYQRLVNQLFAPLIGNTMEVYVDDMLVKSRTADQHIPNLSAMFTILKQYKMRLNPTKCAFGVASGKFLGFMI

>c0039-RT_gypsy_Tekay

VKKKDGTMRLCIDYRQLNKVTVRNKYPLPRIDDLFDQLRGAKVFSKIDLRSGYHQLRIKEEDVPKTAFRTRYGHYEFLVMPFGLTNAPAAFMDLMNRVFRRYLDRFVIVFIDDILVYSKSQKAHMKHLELVLKTLRRKKLFAKFSKCQFWLDRVNFLGHVI

>c0044-RT_gypsy_Tekay

VKKKDGTMRLCIDYRQLNKITVRNRYPLPRIDDLFDQLKGAKVFSKIDLRSGYHQLRVREEDVPKTAFRTRYGHYEFLVMPFGLTNAPAAFMDLMNRVFRRYLDRFVIVFIDDILVYSKSQKAHMKHLNIVLRTLRRRQLYAKFSKCQFWLDRVSFLGHVI
