# Supplementary material for: Genome-wide identification of Reverse Transcriptase domains of recently inserted endogenous plant pararetrovirus (Caulimoviridae)
Source: Front Plant Sci. 2022 Dec 14;13:1011565. doi: 10.3389/fpls.2022.1011565 (PMC9794742; doi:10.3389/fpls.2022.1011565)
Supplement: Supplementary Data Sheet 6 — Wendovirus sequences and polypeptides encoded. [file DataSheet_6.docx]

>CitIch-033; *Citrus ichangensis*; Scaffold_25 (912321-919607dir); https://www.citrusgenomedb.org/

TGGAAACAACATTAATAGAATTAAAGGAAATTCTTTTAAATCTTAAAGAAGAAATGAGAGAAATTAAACAAGAAATAGGAAAAATAAAAATAGAACTATCTACAATACAGATTAGTCGTAAGAAAAATAAGAATATTGAGAGACTAGAAATAATAAAATCAGGAATAATTGGAACAAGTTTTGAAAAACCGATAATAAAACCTCAACTAGGAAGTTATGATGATTGGTTTATAAAAACCCAATACCCTAAAATATTAGAGGAATCAGAAAAATTAAAAACAGAATTAAAGAAAAAAGAAAAGGGAAAAGAAAAGTTAGAAATAAAAGAAACCACACCTAGTGACACAGAATAATGGAAAACACATTAGAAGAATTAACAGAAAGATTTAGTGATATTAATCTTAGTGATTTGGAAGAATTTGAAAAAGAATATTCTATAAAAATGAATGTTGCTGGAGGACCAGAATTAAAACCTGATATAGGAGAACCAAGCCATCAATATGGGAGAAGCAAAAAGAAAAATAAACCATTTTTTGAATATCACCCACCCAATAAAAATACACCTTGGAAAAAAGAATACCAGCCTAATAATGACCACTTAATACCAATAAGTAATGCAGGTACATTTTTAGACTTAGATTGCAAAATAGATCCCCGTAAGGCTTTAGTAGAATGGGGAACACAAATGAAATTATTTTTTATATTTAATGGATCTAAAGATGATTGGAATGTAGATACAGAAGAAGAAAAAATAAACATATTTGTTGACATATTAATAGCAAGTTTTACAGGAAATGTATTTAATTGGTGGAAAGGATTAAGTGATGATACACAAAATTTAATAAAAGATTCAACCAAATTAGCATTAGGAAGAAATAAAGGATTAGGTATTGAAAGAATAATCGAATATATTGCTAGTGAATTCTTAGGAGAAGATTGGTTACAAAATACAGAAAATGAAGCAAAATATGATAAATTAGATGCTAGAATGAAATTAATAAATTTGACAATTTGTAATATGTGTTTCGTAAAAGAATATACATGTGAATTTAGCAAATACTATTATACACAATTCATGAGTCATGAAGACCAAAATATTTATAAGAATTTATACTACTCTAAATTACCATACCCTTGGAACGGATATTTTATTAATGAATATGAAAAATATACTCACACTAGTGATAATAGACTCCCGGATACATTAGGAGGTAGAATAAGATTCTTAAATACAAGATTAAGTGAAATATGTGTCCAGAGAAGTTTAATAAAAAAGAGTAAAAATATAACAAAAATATGTTGTGAAAAAACAGAAATGCCTACTCAATGGGGATGTTATGTACCCCATAAAAAGCGTAGAAAATCTTATCATAAAGAGAAGAGATATAAAAAATATAATAATTTTAAGAAAAAATATAAAAAATCAGGAAGAAAATATTATAAAAGAAAATATAAAAACCAAAGAAAAATATTAGACAAATCCAAATGTAAATGTTGGAATTGTGGAGAGATGGGACACATAAGTACAGATTGTACAAAAAAGAAAAAGGTTAAACTCTTAAAAGAAGATTTTGAGAATATAGAAAGTGACTTAGAAGAAATAAATAATTTATCTGATTATATAGGAGAAGAGATCTATATGGCAGAAGAATCAGAAAATGAATCAGAATAAAGAAGAGTTATTAGATAATGAAAAAATAGAAGAAATAGAAGAAATAGAGAAAGAAGAAAATAAAAGTGGATACAGCCTGGTCGCTACCTTTAACAAAGAAAAATATAATAAACTTTTAAAATTAAACATGGAAAAACCTGAATATGGAATAATCCATAGATTCAGAGATATGTTTAAAAGAAAACGAATTATTTTACATGAATATTATGAACAAGAGAAAGCTGTAACTATCACACAAGCTGAAGGAAATATTAGATTTAGTCTAATAAATAAAAGATCAATAGACTTAGCATTAAGAAAAATAAAGAATGAGTCAACTAAAGAAAAAATTCAATATGTATACCTTGCTGAAATACAAATCTTAATAAAGTCTCTTTTTAAAGAGGGAATAGATAGCCCCATAGTCTTGTCATTACACGATCAAAGATTTACAGATCCCAGTATAGGACATCTTGGAACAATAGAAGGAAATTTATGTTACACTAAATTATTATTTACCTGTCACCCTAGATATTGTGTACACATAAAAGATAAAAACATAGATGAGACATTAAGTTTACATTTTAAACTATTAAAGAAGAATTTAATGAAAGAAGGTAATAGAATAATGACAATACACTATTCAGCTTTATATAGTTTTTGTAATTCAAATTATGGAGAAATATATGGAAATAAACCTTACATTGAGATAAATAAAGATTGTGAAGATATAGCAACATTTATAGAACCGGTAATCCAAGATTACAAAATCCCGATTCGTTATGAATTAAATATAGATGATAGAAAACAATTTTTAGATAACAGTAATGAAAATTCCATCATTCCTATAAGTTTTGCAGGAAGAACTTTGATTAGAGAAAGTTCATCCAAAAGATTAAGCACTGATAAACAATTAGTTAAATCTATCATAACTAATAATGTAATAAAAATACTTGGACAATATTTTAATGGAATAGACTATACAGCAAAAATACCAATACTAATAGATACTGGTACAAGTCATAACTATATGAATCACACAAAAAATAAAGGACTTTTGGTAAAAGAAAAAGAAATTCCTTATGAATATACTGATTTTAATGGCAATAAACATATCTGTAAACAAGAAGTTAAAGTCCCTATAATAATAGAAGGACTTAGAATAATAGTTCCTTGTTACATAGATTCATTTATGACCACAGATCCAGAAAAACACATAGTACTGGGAAATAGTTTTTTAAATAATTTGGAATACTACAGGATAGAAAAAAATAAAATAACTTTACAAATACAAGGTCAAAAAATAGTATGTGAAACATGTTAAAGCAAGCTTATATAAAATACCCTATGTCTGTATATATACAAATAAATATAGTAGAAAAAGATATAGATACTTGTGCTTTAATTGACACGGGTAGTGAAATAACTATTATGAAATCCTTTTTATCCAACAAGTGGGAATTAAATGGAAAATTAAAAATTATTGGAATCACAGGAGATAAACAACAAGTAAATCAATCTATACTAAACTTTGAAGTATTATTAGGTAGCAAAATAATACGTATAAACCAAATCTTTCAATATAATAATATGGATTGTGATCTCCTTTTAGGCAACGATTTCATACAACAGTTTCAATATTACCAGCAAACTCCATATATGATCACTTTAAAAACACCTTGTAATCATATTCTTCGTATTCCACGTGAATTTAAACCTTACAGAGTTCAGCCAGCCCAGCGTGGTGATAAGCATATTTATGAAAAATATTATTTAATACAAAAAGGACAGAGTTATAATATAAAATTAGAAGCCATAAAATCACAACTTGAACAAGTTTATAAAGAAAACCCGTTAGCATTATGGAAACCAAACCATCCTAGAGCAAAAATAGAGTTAATTGAAAATAGGATAATAAGATTAAAACCCATGATGTATACAGAAGAAGATAGAGAAGAGTTTAAAATCCAAATAAAAGAATTATTAAATTTAAAACTTATTAGAAGTAGTAATAGTCCACATAATAGTCCAGCATTTATGGTTAGAAAAAGGGCAGAACAAGTTAGAGGAAAAGCAAGAATGGTAATAAACTATAAAGAACTAAATAAGTATACAAAATTTGATGGATATTTTCTCCCAAATAAAGAAGTACTAATAAACCTTGTAAAAAATAAGACCTACTATTCAAAATTTGATTGTAAATCAGGATTCTGGCAAATAAAAATGGAAGAAAATAGTATACCTTATACAGCTTTTAGCACCCCACAAGGACATTATGAATGGCTAGTAATGCCTTTCGGATTAAAAAATGCCCCACAAATTTTCCAAAGTCGAATGGATAAAATATTTAGAGATTACAACTATATAATAGTCTATGTTGATGATATATTAATAGCTTCAGAAACATTAGACGAGCATAGGCATCATTTAAAAGAATTTGTTAATATTTGCATCAAAGAAGGCATAGTACTGTCCAGTAAAAAAGCGATAATAGAACATAAGAAAATAGAATTCTTAGGTATGATTCTCGATAAACATGGAATCAAACTCCAAAGTCATATAGGAAGAAAAATAACTGAGTTTCCAGATAAATTGGAAAATAAACAACAAATACAAAAATTTTTAGGATGTTTAAATTATGCAGAAGGATTTATAAAAGATCTGTCAAAAAAGAGGAATATACTTCAGAAATTATTAAGGAAAAATAATACTAGAGGATGGAATGAGGAACATACAGAAACTGTAAAAAATTTGAAAGAAGAATGTAAAAATCTTCCAACCTTAAGATTACCAAATGAAAATGATAAATTAATAATACAAACGGATGCATCTGATTTATATTGGGGAGCAATACTCAAAACGGACATAAATGAAATTTGCCGATATACTAGTGGAACTTTTAATCAGGCTCAGGTTAATTACCCTGTACATGAAAAAGAACTCCTTGCAATATACAAAGGAATTAAAAAGTTCTCATTATTTTTACTTCAAAAACAATTTATCATAGAAACTGATAATTCTCAGGTAAGTTCTCTTGTTAATAGAATTTTACCTAATGAACCCCAATACAGAAGATTGCATAGATGGCAAGCTTATTTGTCATATTACAATTTTAAAATTGTTCATATAAAAGGGACTAACAATTTTCTTGCAGATTTTCTAAGCAGGAACATAGAATTTGGAAAATGAATCCTGAAGTAAATAGAATCCTCGGCAAAATAGAAGCCGAAATACAAAGGCTCAAAACTGAAGCATCTGACATGATTGATGCAATAAACTATTTAAAAGAGGCTTCTGAGTCTCATGAGAAAGGAGTAAATTTGTTAGAGTCATACATAGACGACTTAACAAAGGCACTCTCTATAAACACAAATCCAATAAACACGGGAAGCAGTAGTGGACCCTTCAGAGAAGATAAACCAATCTCTGACCCAGCTCCAAATAAAACCATATCCATAGAAGTTGAGTCACCAAAATCAGATCCTCCAAAAAAGTTTATCCAAATAAATACTCCTTATACAGAAGAGGAGGAGAAACCCCTAAATCCAAAAGAAGAAGAATATAAACAATTCTCAACCTGGATATATTCAATTTGTTCCCATAATAATACAATTTTTAAAGCACCAGGGAAAAATATGTACCCCAGAATGATTGCTACAAAAGGAGCAGATCCACTCCTTGTAGGTAAAGTAGCAAAATATGGGTATTTAGATTTAGTATATCCAGACATAGACCTTCGGGAAATAGCCAGTCTTGATGAACTTTTAATAAAAGAAGTCTCAAGATTTGCCCAAAAAAGACCCATATACCTAAAATTCTATACCATAAGCCCAGAATACTACCAAAATGTCGAATACCAAGCCTTTTATATGATCTCTGTAGGTCACCTCACAAAAAATTTCAAGGTTGAAGTGGGAACAGAATATTCAAATGTCCCTCAGATAAATGAACCTTGGATCAGAACACGAAGAGCCAGAGGAATAAAGGCCATATGGAGCATAGCTAAAGATCTATATAAAAGGAATTTCAGAACCATTATCAAATTCCAGAATTACATCCTTATAACTTCAGAAGATGGCCATACACCTTCAAATCTGTTACTAGACTTCATCAACGAAATAAGCACTATGCGTTTCCCCAGTAGCAGTAAAACCATGGAGCAAGCTTATCAACTCATGGAAGTGGCAGACAACACAAAAAGGATGCCCGACAAGATAAGGCAGACAGCATAAAGAAGATGCCCGACAAGATAAGGTGGAAACAGCATAAGTGACAAAATTAGAACTGTACATGGAATAAAAACGTAAGCAGTGATGTAAGAAGCCCAATTATGTAAGGGCAAAAATGTAATAAAATAGAAAAAGAATGGAAAAAGCCCAATCATACAAGGGCAATAATGTAATCATCTTGGCGATATAAATAAAAGAGAAGAGAAAAAAAAGGCTCTCAGAGCTTATCATTCACCATATTTCAATCTTTGAAAAGATTCTCTGAAAATATGAACGGTTAAACCACTTCCCTGCTCTAAATCCAAAATCAATACATCACTGACCAAAAAACAAACAACCCCATAGAAAGGAAAGAGAAACCTAAATTTGTTCAACATTTCTAAGCCAGACGATCAAGATCCATACAGTAAAGCCGATCGAGTCCGCTGATCCCGTTGAGGCAAGGAGGCCGTTGAGGGAATAAGGACAACCAGGCTGCAGGTCACTGGAAAAGGATTGATAGGCAAGGAAACAAGAATAAGCTAGGTAATAATTTCAAACCTTATCTGTTGGTGGAAGTTTGTTGGTGATCAATGATGAGGAGATTTGTGGAGGAAGAGCTAATTTAAACATGAATAATAATGTTTTGATCGGAAATTCTAAAGAATATGAGATAAACTCAACAACTAGAATAACATATAATGATTATATGTTAATAATAGATTTTAATTTAGGATCTTTTAAACAAAGTTCATATCTTTTCAGTAAAGAGGAGCTTGTTAGGTCAGAAGATCTACAAGCAGTAAATACACTTAACTTACCAAAAGAAATAAAAAGAAAAATTATAAACCATATCGAAATAGATAACTTAAAGCATAATATAATCATGTTAACAATAGAACAAGGAATTGATATAGCCTTAGCATATATAGAACATGAAAGTCGAGAAATTGGACCATCATACATAAATTATATAGAGAATTCGGATAACGAAGAACTTGAAATATAAAATCAAAATAAAACACATATACTGGTTATAAGGTATATCGGCAAGCAACCCATAACGTAAATTGGATAGACGATCCCGATTGGGCGTTAGTATCCTCTGCACTTTGGGAAAACAAGCCACGTACCTATAACTAGAATACTTTTTAAATATAGAGACTTACAGGGAAATCAGATATTGTATTAGTATAATAAACGATAATATATATTATATAAAAATAAAAATATCCTATGATTCTAATACTACATATAATCCAGAAATACCGGATTTGGATCTGACTAATAATCCTACATATATTTTAGAAACTAATAATAGTTATTATGATTTTTATTTCCCTACTTTTCCTAATAACATATCTAGAATAATTGGAGGAGAACAAATAATAGCCCTATATTTGAGGAATAATAGTAATACAGGATATG

zf-CCHC_6 Zinc knuckle (coat protein) e-value 2.2e-06

MP Movement protein e-value 4.3e-31

Gag-asp-protease Aspartyl protease e-value 0.062

Gag-asp-protease Aspartyl protease e-value 0.065

RVT_1 Reverse transcriptase e-value 4.9e-28

RT-RNAseH_2 RNAseH e-value 7.4e-24

>ORF1

MENTLEELTERFSDINLSDLEEFEKEYSIKMNVAGGPELKPDIGEPSHQYGRSKKKNKPFFEYHPPNKNTPWKKEYQPNNDHLIPISNAGTFLDLDCKIDPRKALVEWGTQMKLFFIFNGSKDDWNVDTEEEKINIFVDILIASFTGNVFNWWKGLSDDTQNLIKDSTKLALGRNKGLGIERIIEYIASEFLGEDWLQNTENEAKYDKLDARMKLINLTICNMCFVKEYTCEFSKYYYTQFMSHEDQNIYKNLYYSKLPYPWNGYFINEYEKYTHTSDNRLPDTLGGRIRFLNTRLSEICVQRSLIKKSKNITKICCEKTEMPTQWGCYVPHKKRRKSYHKEKRYKKYNNFKKKYKKSGRKYYKRKYKNQRKILDKSKCKCWNCGEMGHISTDCTKKKKVKLLKEDFENIESDLEEINNLSDYIGEEIYMAEESENESE

>ORF2

MNQNKEELLDNEKIEEIEEIEKEENKSGYSLVATFNKEKYNKLLKLNMEKPEYGIIHRFRDMFKRKRIILHEYYEQEKAVTITQAEGNIRFSLINKRSIDLALRKIKNESTKEKIQYVYLAEIQILIKSLFKEGIDSPIVLSLHDQRFTDPSIGHLGTIEGNLCYTKLLFTCHPRYCVHIKDKNIDETLSLHFKLLKKNLMKEGNRIMTIHYSALYSFCNSNYGEIYGNKPYIEINKDCEDIATFIEPVIQDYKIPIRYELNIDDRKQFLDNSNENSIIPISFAGRTLIRESSSKRLSTDKQLVKSIITNNVIKILGQYFNGIDYTAKIPILIDTGTSHNYMNHTKNKGLLVKEKEIPYEYTDFNGNKHICKQEVKVPIIIEGLRIIVPCYIDSFMTTDPEKHIVLGNSFLNNLEYYRIEKNKITLQIQGQKIVCETC

>ORF3

MLKQAYIKYPMSVYIQINIVEKDIDTCALIDTGSEITIMKSFLSNKWELNGKLKIIGITGDKQQVNQSILNFEVLLGSKIIRINQIFQYNNMDCDLLLGNDFIQQFQYYQQTPYMITLKTPCNHILRIPREFKPYRVQPAQRGDKHIYEKYYLIQKGQSYNIKLEAIKSQLEQVYKENPLALWKPNHPRAKIELIENRIIRLKPMMYTEEDREEFKIQIKELLNLKLIRSSNSPHNSPAFMVRKRAEQVRGKARMVINYKELNKYTKFDGYFLPNKEVLINLVKNKTYYSKFDCKSGFWQIKMEENSIPYTAFSTPQGHYEWLVMPFGLKNAPQIFQSRMDKIFRDYNYIIVYVDDILIASETLDEHRHHLKEFVNICIKEGIVLSSKKAIIEHKKIEFLGMILDKHGIKLQSHIGRKITEFPDKLENKQQIQKFLGCLNYAEGFIKDLSKKRNILQKLLRKNNTRGWNEEHTETVKNLKEECKNLPTLRLPNENDKLIIQTDASDLYWGAILKTDINEICRYTSGTFNQAQVNYPVHEKELLAIYKGIKKFSLFLLQKQFIIETDNSQVSSLVNRILPNEPQYRRLHRWQAYLSYYNFKIVHIKGTNNFLADFLSRNIEFGK

>ORF4

MNPEVNRILGKIEAEIQRLKTEASDMIDAINYLKEASESHEKGVNLLESYIDDLTKALSINTNPINTGSSSGPFREDKPISDPAPNKTISIEVESPKSDPPKKFIQINTPYTEEEEKPLNPKEEEYKQFSTWIYSICSHNNTIFKAPGKNMYPRMIATKGADPLLVGKVAKYGYLDLVYPDIDLREIASLDELLIKEVSRFAQKRPIYLKFYTISPEYYQNVEYQAFYMISVGHLTKNFKVEVGTEYSNVPQINEPWIRTRRARGIKAIWSIAKDLYKRNFRTIIKFQNYILITSEDGHTPSNLLLDFINEISTMRFPSSSKTMEQAYQLMEVADNTKRMPDKIRQTA

>CitMax-350; *Citrus maxima*; chromosome8 (12918254-12917352rev); https://www.citrusgenomedb.org/;

GGCCAAGCCTATGTTACATGCAACGTAACATACATTCAAATAAACAAAATATTGATAGACCCCCTTATAGTTTATGGAAACCACGATAATAGAGTTAAAAGAAATACTTTTAAATCTAAAAGAAGAAATAAAAGAAATAAGACAAGATATAGGAAAATTAAAAATAGAGATGTCACTGTTACAAAAATGTAAAAAGAAAAATAAGAAAATAGAAAGACTTGAGATGATAAAATCAAATATTATTGGGACAAACTTAAAGAAATCTACACAAAAACCCCAAAAAGGCAGTTATGACTACTGGTTTATGGAGACACAATATCCAAAAATATTAGAGGAAGCTGAAGAACTAAAGGCAGAAATAAGAAAGAAAGAAAAAGGAAAAGAGAAATTAGAAATAAAAGAACAAACTGAGTCTAATTCTGAATAATGGAAGACACAATAGAAAGTTTAACTGAAAAATTTAGTGACATAAATCTTAGTGATTTAGAAGAATTTGAAAAAGAATACCATATAAAAATGAATACAGCAGGAGGACCAGAAATGAAACCAAATGTTGCAGAACCAAGCAATATACCTAAGAAAAAGAATAAACCTTTTTATGAATACACACCTAAAACAAAATCTACTCCATGGAAAAAAGAATACAAACCTAATCAAGATACATTAGTACCTATAAGCAATAGTGGATCATTTTTAAATATAGACTGTAAAGAGAACCCTCGAAAAACATTAGCAGAATGGGGAATGCAGATGAAGCTATTCTTCACATATAATGGAGCTAATGATGACTGGAATGTAACCACTAATGAAGAAAAGATAGACATATTCACAGATATCCTAATAACTAGTTTTACAGGGAATGTATTTAATTGGTGGAGAGGATTAAGTGATGATACACAAATGTTAATAAAAGACTCAACCAAAATAGCTTTTAGGAGGAGCACTGCTCTAGGAATAGAAAGAATACTGGAATACATTGCTAGAGAATTTTTAGGAGAAGATTGGTTAGAAAATTCAGAGAGAGAAGCAAATAATGAAAAACAAAGAGCACGGTTAAATCTATTAAATTTAACAATATGCAATATGTGTTTTATCAAAGAATATACTTGCGAATTTAGTAAATATTATTATGAACAATATGTAAGTTTAGAAGATCAAGAAGTTTATAAAAACTTATATTACTCCAAGTTGCCATACCCTTGGAATAGTTACTTTATAAATGAATATGAAAAAATTAGTACTTCAAATAAACTCCCAGATAACCTAGGATCTAGAATAAGATTTATAAATACTCGACTTAGTGATATATGTATCCAGAGGAATTTAATCAAAAAGAGTAGAAACATAACAGAAATATGTTGTGAAAAAACAGAGATGCCAACTCAATGGGGATGTTCTATCCCATATTATAATAGAAAAAGAAAATATAAAAAAGAAAAAAGATATAAAAAATACAACAAATTTAAAAAGAAATACAAGAAACCTGGAAGAAAATACTATAAAAGAAAATATAAAAACCAAAGAAAGTTAGTAGATAAATCAAAATGTAAATGCTGGAATTGTGGTGAAATAGGACATATCAGTACAGATTGTAAAAAGAAAAAAGTTAGAATTTTACAAGAAGATTTTGAACAAATAAGAGATGAATTAGAAGAAATAAAAAACATATCAGAATACGAAGGAATGATAGTATATATGGAAAATACAGAGGAAAATGAATCAGAATAAAGAAAAAGACATATTGGAAAAAGAAGAAATAGACGACATTGAAGAAATAGAAAGAGAAGAAAACAAAAGTGGGTATAGTTTAGTAGCCACTTTTAATAAAGAAAAATACAATAAACTTCTAAGATTAAATGTGGAAAAACCGGAGTATAGTATCTTACAAAAACTAAGAGATTTATTTAAAAGAAAAAGGATAATTTTACATGAATACTATGAAGAAGAAAAAGCTGTAACTATAACACAAGCTGAAGGAAACATACGATTTAGCTTAATAAATAAAAGAACAATAGATCTAGCCTTAAGAAAAATAAAAAATGAATCAACAAAAGAAAAAATTCAATATGTATATTTAGCTGAAATCCAAATCTTAATAAAATCCCTCTTTAAAGAAGGAGTAGATAGTCCAATAATTTTATCAATTCATGATCAAAGATTCCATGATCCTAGTATAGGGCATCTAGGAACAGTAGAAGGAAACTTATGTTATACCAAATTACTATTTACATGTCATCCTAGATACTGTGTACACATAAGGGATAAAAATATAGATGAAACTTTAAGTCTACATTTTAAACTCCTAAAAAAGAACTTAATGAAGGAAGGAAATAGATTAATGACTATACACTATTCAGCATTATATAGTTTTTGTAACTCAAATTATGGAGAAATCTATGGGAATAAACCATATATAGAAATAAATAAAGAATGTGAGGAAATAGCAACTTTGATAGAACCTATAATACAGGATTATAAAATTCCTATAACATACGAATTAGATGTAGATGATAAAAAACAATTTTTAAATAATAATAATGAGAATTCTATAATCCCTATCAGTTTTGCCGGAAGAACTCTTTTGAGAGGAGAATCTTCTAGAAGATTAAGTACTAATAGACAAATAATCAATATAAATAAACAAGTCTTAAAACCTACTCTTACTAGTAGTGTAGTAAAAATCCTTGGACAATACTTCAATGGAATAGAATATACAATAAGGTTACCTATCCTAGTAGATACAGGAACAAGTCATAATTATATGAATCATACAAAAAATAAAGGACTCTTAATAAAAGAAAAAGAAATTCCATATGAATATACTGACTTTAATGGTAATAAGCACATATGTAGACAAGAAGTAAAAGTTCCATTAATAATTGAAGGAATACGAATAATAGTATCTTGCTACATAGATTCTTTTATGACTACAGATCCAGAAAAACATATAGTCTTAGGAAATAGTTTCTTAAATAGTCTAGAATATTATAAAATAGAAAAAGACCAAATTACCTTACAAATAGAAGGACAAAAGATAATTTGTGAAACATGTTAAGACAAGCATATATAAAATATCCTATGTCTGTTTATATACAAATAAATATAGTAGAAAAAGATATAAACACTTGTGCCTTAATAGACACAGGAAGTGAAATTACAATCATGAAAACCTTTTTATCAAACCAATGGAAATCAAATAGCAAGATAAAACTCGTAGGTATCACAGGAGACAAACAACATATTACTCAATCTTTAGAAAGCTTTGAAACTATTTTAGGAAGTAAAATTGTTCGTATTAATCATATCTTCCAATATGACCAAATGGACTGTGATATACTTTTAGGAAATGATTTCTTACAACAATTTCAATATTACCAACAGACGACCTATATGGTTACTCTTAAAACCCCATGCAATCATATTTTACGAATTCCTCGAGAATTTCGCCCTTATAGAGTTCAGCCAGCCTCGCGTGGTGATAAGACAATTTATGAAAAACATTATTTAATTTCAAAAGGACAGAGTTATAACATAAATATAAAATTAGAAACAATAAAATCCCAACTAAAGCAAGTCTATGAAGAAAATCCATTAGCCTTATGGAAACCAGATCACCCCAGAGCAAAAATAGAATTAACAGAAAATAGAATCATAAGACTTAAACCTATGATGTACACAGAAGAAGACCGAGAAGAATTTAGAATCCAGATAAAAGAATTATTAGAATTAAATTTAATACGCAATAGTAATAGTCCACACAACAGCCCAGCTTTCATGGTTAGAAAAAGAGCAGAACAAGTTAGAGGGAAAGCTAGAATGGTAATAAACTATAAAGAACTAAATAAATATACCAAATTTGATGGATATTTTTTGCCAAATAAAGAAGTTCTAATTAATTTAGTAAAAAATAAAACTTATTATTCAAAGTTTGATTGTAAATCAGGTTTCTGGCAAATAAAAATGGAAGAAAATAGTATTCCTTATACAGCATTTAGTACTCCACAAGGACATTATGAATGGTTAGTAATGCCCTTTGGATTAAAAAATGCTCCACAAATATTCCAAAAAAGAATGGATAAAATATTTAGAGATTATAAATACATAATAGTATATGTTGATGACATATTATTAGCCTCTGAAACCTTAGAAGATCACAGAAAACATTTAAAAGAATTTGTAAATTTATGTATTAAAGAAGGGATAGTATTATCAGAAAGAAAAGCAAAAATAGAACATAAGAAAATTGAATTTTTAGGAATGATTATAGATAAACAAGGAATCAGACTCCAAAGCCATATAGGAAGAAAAATTATTGATTTTCCAGATAAATTAGAAAATAAACAACAAATACAAAAATTCTTAGGCTGCTTAAATTATGCAGAAGGATTTATAGAAAATCTAGCTAAAAAGAAAAATATACTTCAAAAGTTACTTAGGAAAAATAATACTAGAGGATGGAGCATAGAACATACAGAAACTGTAAAAAACTTAAAAAATGAATGTAAAGATCTTCCTTCTTTGAGATTACCAGATAAAACTGATAAGTTAATAATGCAGACTGATGCATCTGACCTATATTGGGGAGCAATACTTAAGACTGATATAAATGAGATATGTAGATACACCAGTGGTACTTTCAACCAAGCACAAGTCAATTATCCTGTCCATGAGAAAGAGCTACTTGCAATATATAAAGGAATAAAAAAGTTTTCTTTGTTCTTATTACAAAAACATTTTATTATAGAAACTGACAATTCCCAGGTAAGCTCATTAATAAATAGAATTTTACCAAATGAACCCCAGTACAGAAGGTTACATAGATGGCAAGCTTATTTATCATATTATAATTTTAAAATTATACATATAAAAGGATCTAACAACTTTCTTGCAGATTTTTTAAGCAGGAACATAGAATTTGGGAATGGATCCTGAAATAGATTTGATCCTTGCCAAAATAGATGATGAACTACATAGACTGGCAACAGAAGCTTCAGATATAATCGATGCTGTAAATTATTTGAAAGAGGCTTCAGAAGCCCATGAAAAAGGAGTACATTTGTTAGTGTCGCAAATAAATGACCTGACAAAAGCACTCTCAATAAAACAAGGCCCAAAGAATATTGCGCCAATAAACACCAGCCCAATTGGTGAAGGAAGCAGTAGTGGACCCTTCAGAGAAAATAAACCAATCTCTGACCCAATCAAAAACCCACCAAATATAATATCCATCGAAGTTGAGCCACAAAAATCAAGTCCTTCAAAAAAGTTCATCCAAATAAACAATCTCCAAATAAAAGAAGAAGAGAAGGAACCTTTGAATCCAAAAGAAGAAGAGTATAAACAATTTTCTACATGGATTTATTCTATCTGTTCCCACAATAATAATATCCATAAAGCACCAGGTAAAAATATGTATCCCAGAATGATAGCCACCAAAGGAGCAGACCCACTTCTTGTAGGAAAAATAGCCCGTTATGGTTATCTAGATTTAATATATCCTGATATAGACCTCCGAGAAATAGCCAGTTTAGATGAATTACTAGTCAAAGAAATATCTAAATTCGCCCAGAAAAGACCCATATACCTCAAATTCTACACCATAAGTCCAGAATATCATCAAAATGTAGAATACCAAGCATTTTATATGATTTCAGTGGGTCATATAACCAAAAATTTCCAATTAGAAATAGGAACCGAATATACTAATGTTCCTCAGATTACAAAAAATTGGATCCGAACCCGAAGAGCTCGAGGCATAAAAGCAATCTGGAGCATAGCAAAAGACCTCTACAAAAGGAATTTCAGAACCATCATCAGATTTCAAAATTATATCTTAGTAACAGCAGAAGACAGCCATACATCCTCAAATATCTTGTTAGAGCTTATTAATGAAATAAGCACTATGCGATTTCCCAGCAGCAGTGAAACTATGCAGCATGCTTATCAGCTTATGGAAGCAAGCAATCAGACAAAATAAAAAGATGTGATCATATAGTGGCAGACAGCATAAAGAAGATGCCAGACTGATGTGATCATATAGTGGCAGACAGCATAAAGAAGATGCCAGACAAAAAGAAGATAAAAAGAAGCCGACAAGATAAAAAGCAGAAAAGCTTTGTCTTAGCAATAATGTAAAGCACTTGTAATAATTAGAAAAAGCCAAAAGGGCAAGAATGTAATCTCGAATAGAAAGTTTTGAAATAAAAAGAAAAGAAGAACGAGGGATACCTCAGATTGAAAACTCAGATTGAAAAACCATACTTCACTTTTTCAAAACTCTCTGAAACATGCATAGCTAAATTCTCTTAAGCTCGAAACCATAAATCAATACATCTTTCAATCATAAACAAACAACCCCATAGAAAGGAAAGAGAAACCTAAAATTATCCCTGATTCTAAGAAAGAAGATCCAGATCCATACAGAAAGGCCATCAGAGTCCACTTGATCCCGTTTAGGCAGGAGGCCGTTGAGGGAGTGAAGGACGCGTAGGCTGCAGGTCACTGGAGACAGATCTTAAGACGAAGAAAAAAGGATAACTTAGGTAATAATTCTCTAACCTTATCTGTTGGTGGAAGTTTGGTGATGATTGTAGATGAGGAGATTAATGGTGGATGAGCATCTCTTAGAAGTGAATTTTATTCAAATTATTAATCAAAAATTGTTAAAGAAATAAGAAAATGAGTGTTTATCAAAATATGAGTGTAAGATTAGGACGTGGAAATCCTAAAGAAAAAATGATAAATCCTACAACTAGGATTACATATAATAATTACATGATGATAATAGATTATAATCTTGGATCTTTTAAACAAAGTTCATACATATTTAGTAAAGAAGAAGTTATTAGTAAAAATGAAATACAAGCTATTTATAGCCTAGAATTACCACCTGAAATAAAAAGAAGAATCTTAAACATTTTAGAAACGGATAATCTAAAACACTTAATTACACTATTAACTGTAGATGAAGCTATTGATATAGTTAAAGCTAAAATAGAAAATGAAAGCTTAAAAATAGAACCTTCTTACGTCGAATATATAAATAATTCAGATAACGAAGAATTAGAATTTTAATATCATGAATAATTATAGGGACATAAGATATTACACTAGTATAATAAATAATAACTTATATATTTTAAAAGTAGAAATACTCCTTTATCCTGTAAACATAACCCATAATTTAGAAATCTTAAATCAAATTATGCATCCTACATATATTATAGAAACCAATAATAATTATTATGATTTTTATGTTCCTAATTTTCCAGGAAATATACCTAGTATCATTAATGGAGAAGAAGTTATAGGATTATATTTAAGGAATAATAACAGTGGGAGATATGATAGATATACATTTTTAGAAGAATAATAATAGTAATATATCTTCTTATCAATTATCTTTCATCCAAAACTTTGGTTTTGTTGTTTGCTTGTTAGTGTATATTTTGTTTTGTTTTATAGATTTTGTTTTATTTGTTTTGTTTTGTTTTGGTTTTGTTTTTGTTTTGTTTTGTTTTTTGTAATAAAAATGGTATCAGAGCCATGGATCCTATAAAAGATATAACAGATAGCTTATATACTTTAAAAGTGTTTGACAAAGAAGAAATAAAAAATTTAATAATGTCTATAAAATCCGAAAAGAATAACATCCGAAATATTGAGCTTAATATAACACAAGAATTACAAGAAAGAAATATAGAAACTCTAGGAGGTGAGATTTTAATAGAAAAGAAAATGAAATTCATAGATGAATTTATAAAATTTTTAGTATACTGGTATAAAAAAGAAGCATTACCATGAACCAACATGCTTTTAATATAATACATAATTTAGCAGAAACAATAGATCATATGAATCTAGGCTGGGAAAATATTATAACAAGTTATATAAATCAAATAACCTAAAAAATAGAGTTATTAGAACAAGAAAGAATGGGAATAATAAATTTAGGACTCGAAATTTATTCAGAATTTACAGATTTATATGAATTCGATAATCATATATTATTGTTTAAAGAGATAAAGAAAATATTAGAAAGAATGAAAAATAGGTGTA

zf-CCHC_6 Zinc knuckle (coat protein) e-value 8.3e-06

MP Movement protein e-value 1.9e-30

Gag-asp-protease Aspartyl protease e-value 0.025

RVP Retroviral aspartyl protease e-value 0.028

RVT_1 Reverse transcriptase e-value 8.2e-29

RT-RNAseH_2 RNAseH e-value 6.2e-22

>ORF1

MEDTIESLTEKFSDINLSDLEEFEKEYHIKMNTAGGPEMKPNVAEPSNIPKKKNKPFYEYTPKTKSTPWKKEYKPNQDTLVPISNSGSFLNIDCKENPRKTLAEWGMQMKLFFTYNGANDDWNVTTNEEKIDIFTDILITSFTGNVFNWWRGLSDDTQMLIKDSTKIAFRRSTALGIERILEYIAREFLGEDWLENSEREANNEKQRARLNLLNLTICNMCFIKEYTCEFSKYYYEQYVSLEDQEVYKNLYYSKLPYPWNSYFINEYEKISTSNKLPDNLGSRIRFINTRLSDICIQRNLIKKSRNITEICCEKTEMPTQWGCSIPYYNRKRKYKKEKRYKKYNKFKKKYKKPGRKYYKRKYKNQRKLVDKSKCKCWNCGEIGHISTDCKKKKVRILQEDFEQIRDELEEIKNISEYEGMIVYMENTEENESE

>ORF2

MNQNKEKDILEKEEIDDIEEIEREENKSGYSLVATFNKEKYNKLLRLNVEKPEYSILQKLRDLFKRKRIILHEYYEEEKAVTITQAEGNIRFSLINKRTIDLALRKIKNESTKEKIQYVYLAEIQILIKSLFKEGVDSPIILSIHDQRFHDPSIGHLGTVEGNLCYTKLLFTCHPRYCVHIRDKNIDETLSLHFKLLKKNLMKEGNRLMTIHYSALYSFCNSNYGEIYGNKPYIEINKECEEIATLIEPIIQDYKIPITYELDVDDKKQFLNNNNENSIIPISFAGRTLLRGESSRRLSTNRQIININKQVLKPTLTSSVVKILGQYFNGIEYTIRLPILVDTGTSHNYMNHTKNKGLLIKEKEIPYEYTDFNGNKHICRQEVKVPLIIEGIRIIVSCYIDSFMTTDPEKHIVLGNSFLNSLEYYKIEKDQITLQIEGQKIICETC

>ORF3

MLRQAYIKYPMSVYIQINIVEKDINTCALIDTGSEITIMKTFLSNQWKSNSKIKLVGITGDKQHITQSLESFETILGSKIVRINHIFQYDQMDCDILLGNDFLQQFQYYQQTTYMVTLKTPCNHILRIPREFRPYRVQPASRGDKTIYEKHYLISKGQSYNINIKLETIKSQLKQVYEENPLALWKPDHPRAKIELTENRIIRLKPMMYTEEDREEFRIQIKELLELNLIRNSNSPHNSPAFMVRKRAEQVRGKARMVINYKELNKYTKFDGYFLPNKEVLINLVKNKTYYSKFDCKSGFWQIKMEENSIPYTAFSTPQGHYEWLVMPFGLKNAPQIFQKRMDKIFRDYKYIIVYVDDILLASETLEDHRKHLKEFVNLCIKEGIVLSERKAKIEHKKIEFLGMIIDKQGIRLQSHIGRKIIDFPDKLENKQQIQKFLGCLNYAEGFIENLAKKKNILQKLLRKNNTRGWSIEHTETVKNLKNECKDLPSLRLPDKTDKLIMQTDASDLYWGAILKTDINEICRYTSGTFNQAQVNYPVHEKELLAIYKGIKKFSLFLLQKHFIIETDNSQVSSLINRILPNEPQYRRLHRWQAYLSYYNFKIIHIKGSNNFLADFLSRNIEFGNGS

>ORF4

MDPEIDLILAKIDDELHRLATEASDIIDAVNYLKEASEAHEKGVHLLVSQINDLTKALSIKQGPKNIAPINTSPIGEGSSSGPFRENKPISDPIKNPPNIISIEVEPQKSSPSKKFIQINNLQIKEEEKEPLNPKEEEYKQFSTWIYSICSHNNNIHKAPGKNMYPRMIATKGADPLLVGKIARYGYLDLIYPDIDLREIASLDELLVKEISKFAQKRPIYLKFYTISPEYHQNVEYQAFYMISVGHITKNFQLEIGTEYTNVPQITKNWIRTRRARGIKAIWSIAKDLYKRNFRTIIRFQNYILVTAEDSHTSSNILLELINEISTMRFPSSSETMQHAYQLMEASNQTK

>CitMed-029; *Citrus medica*; Scaffold_433 (28756-36655dir); https://www.citrusgenomedb.org/;

CTATGTTACATGCAACGTAACATACATTCAAGATGGAAAATATCATTCAGCACCCAAAAATTTTATGGAAACCACAATAAGAGAGTTAAAAGAAATCCTTTTAAATCTCCAAGATGAAATAAAAGAAATAAAACAAGAAATAGGAAAAATAAAAATTGAATTGTCTGTTATACAAAAAAGTCAGAAAAAGAATAAAAAGGTAGAACGACTTGACATAATAAAATCAGGAATAGTTGGAACAAGTTTTAAGAAACCTACTCAAAAACCCCAGAAAGGAAGTTATGATTTCTGGTTTATGGAAACACAATACCCTAAAATATTAAAAGAATCTGAAGAACTAAAAGCAGATATAAAAAAGAAAGAGAAAGGAAAAGAAAAAATAGAAATAAAAGAACAGACTCCATCCGAATCAAACTCTGAATAATGGATAATACATTAGATAATTTAACCGAAAAATTTAGTGACATAAACCTTAGTGATTTAGAAGATTTTGAAAAAGAATACCAAATAAAAATGAATGTAGCAGGAGGACCAGAAATGAAACCTAATGTGGCAGAACCAAGTCATAGACATATGCCAAAGAAAAAACATAGTAAACCCTTTTATGAATATATACCTACTATTAAAAATACCTCTTGGAAAAAAGAATTCATACCTAACCAGGATATATTGACTCCTATAAGTAATAGTGGAGCATTTTTAAACTTAGATTGTAAAATAGATCCACGAAAAACCTTAGCTGAATGGGGAATACAAATGAAACTGTTCTTCACATATAATGGAACTAATGATGATTGGAATGTTACAACAGATGAAGAAAAAATAGACATATTCGTAGATATTCTTCTAACCAGTTTTACTGGAAATATATTTAACTGGTGGAGAGGTTTAAGTGAAGATACCCAACTGTTAATAAAAGACTCAACAAAGATAGCTTTTAGAAGAAGCAATGCTTTAGGCATAGAAAGAATAGTAGAATATATTGCAAGTGAATTTTTAGGAGAAGATTGGTTAGAAAATTCAGAGAGAGAAGCAAATAATGAAAAACAAAGAGCAAGATTAAACTTATTAAATTTGACGATTTGTAATATGTGTTTTATTAAAGAATACACTTGTGAATTTGCTAAATACTATTATGAACAGTACATAAGTTTAGAGGATCAAGAAGTTTATAAAAACTTATATTACTCTAAATTACCATACCCTTGGAATAGTTATTTTATTAATGAATATGCAAAAGATAGCACTTCAAATAGGCTCCCTGATACACTCGGAGCAAGAATAAGATTTCTAAATATAAGACTTAGTGATATATGTATACAGAGAAATCTAATAAAAAAGAGTAAAAATATAACAACCATATGTTGTGAAAAGACAGAAATGCCAACCCAATGGGGTTGTTCTGTACCTTATAAAAGAAAAAGAAAATTCTATAAAAAAGAAAAAAGATATAAAAAATATGCCAAATTTAAAAGAAAATATAGAAAACCTGGCAGAAGTTATTATAAGAGAAAATATAAAAGTCAGTACAAAAGTCAAACGAAACAACTAGACAAAACAAAATGTAAGTGTTGGAATTGTGGAGAAATAGGACACATCAGCACCGATTGTAAGAAAAAGAAAGTAAGAATCTTACAAAAAGATTTTGATACAATACAAGAAGAATTAGAAGAAATAAATAATTTAACAGATTATGAAGGAGAAGAAATATATGTTATAAAAGACTCTGATGATGAATCAGAATAAAAAAGATATAGTTGAAACAGAAGAAATAGAAGATATTGAAGAAATCGAAAGAGAAGAGAATAAAAGCGGATATTGTTTAGTAGCAACCTTTAATAAAGAAAAATATAATAAACTTTTAAAATTAAATGTAGAAAAACCAGAATACGGAATAGTACAGAAATTAAGAGATCTGTTTAAAAGAAAAAGAATAGTGTTACATGAGTATTATGAACAAGAAAAAGCAGTAACTGTTACACAAGCTGAAGGAAATATCAGATTTAGTTTAATTAATAAGAGAACCATAGATTTAGCACTAAGAAAAGTAAAAAATGAAACAACAAGAGAAAAAATACAATATGTATATCTAGCAGAAATCCAAATATTGATAAAATCTCTTTTTAAAGAAGGAATAGATAGTCCTGTAGTATTATCAATACATGATCAGCGGTTCCATGATCCAAGCATTGGACATTTAGGAACAGTAGAAGGAAATTTATGTTATACTAAATTATTATTTACATGCCATCCTAGGTACTGTGTACACATTAAGGACAAAAACATAGACGAAACACTCAGTTTACATTTTAAACTGCTTAAAAAGAATTTAATGAAAGAAGGTAATAGGTTAATGACTATACATTATTCAGCATTATACAGTTTTTGTAATTCAAATTATGGAGAAATTTATGGAAATAAACCCTATATCGAAATAAATAAAGACTGTGAAGATATTGCAACTCTTATAGAACCTATTATGCAAGATTATAAAATCCCTATCACTTATGAATTAAACATAGATGATAAAAAACAATTCTTAAATGAGAATAACGAAAATTCTATAATTCCCATAAGCTTTGCTGGAAGAACTCTTTTAAGAGGAAGTTCATCCAAAAGATTAAGTACTGATAATCAAATAACTAATACACATAATTTAGCTTTAACTAAATCCTCAAGAAATAACAATGTAGTAAGAATATTTGGACAATATTTTAATGGAATAGAATATACCCTTAGGTTACCTATTTTGATAGACACTGGTACAAGCCACAATTATATGAACCATACAAAAAATAAAGGACTTCTAATTAAAGAAAAAGATATTCCATACGAGTATACTGATTTTAATGGAAATAAACATATATGTAAACAAGAAGTAAAAGTTCCTTTAATTATTGAAGGAATTAGAATAATTGTATCCTGTTATATAGATTCTTTTATGACTACTGACCCTGAAAAACACATAGTATTAGGAAATAGTTTTCTAAACAGTCTAGAATATTATAAGATTAAAAAACATCAAATTACACTGAGAATAGAAGGTCAAAAGATAATTTGTGAAACATGTTAAGACAAGCTTATATTAAATATCCTATGTCTGTATATATACAAATTAATATAGTTGAAAAAGATCTAGATACATGTGCTTTGATAGATACAGGCAGTGAGATTACAATTATGAAAACCTTTTTATCTAGTCAATGGAAAGTCAATGGACAAATAAAACTTATTGGCATTACAGGAGATAAACAACATATTAACCAAAGCCTATTAAACTGCGAAATCCTCGTAGGAAGCAAAGTTGTCCGTATAAACCAAGTATTTCAATATAACCAAATGGATTGTGATATACTGTTAGGTAATGATTTCATACAACAATTCCAATATTACCAACAGACAACTTACATGATTACCCTTAAAACCCCATGTAACCATATCCTTCGCATTCCTCGTGAATTTAAACCTTATCGAGTTCAGCCAGCCCAGCGTGGTGATCAGGTTATTTATGAAAAACATTACTTAATTTCCAAAGGACAGAGTTATAATATACGACTTGAAACTATAAAATCACAATTGGAAAAGGTATATAAAGAAAACCCATTAGCATTATGGAGAGCAGATCATCCTAGAGCAAAAATTGAACTGATAGAAAATAGGATCATAAGACTTAAACCTATGATGTATACAGAGGAAGACAGAGAAGAATTCAAAGTCCAAATAAAAGAATTATTAAATTTACACTTAATAAGACATAGTAATAGTCCACATAACAGCCCAGCTTTTATGGTTAGAAAAAGAGCAGAACAAGTTAGAGGAAAAGCTAGAATGGTAATAAATTATAAAGAATTAAATAAATATACAAAGTTTGATGGATATTTTTTACCAAACAAAGAAGTGTTGATAAATCTAGTAAAGAATAAAACTTATTATTCAAAATTTGATTGTAAGTCAGGGTTTTGGCAAATAAAAATGGAAGAAAATAGTATCCCTTATACAGCATTTAGCACTCCACAAGGCCATTATGAATGGATAGTAATGCCTTTTGGATTAAAAAATGCCCCTCAGATATTCCAAAGTAGAATGGATAAAGTCTTTAAGGACTATAAATATATAATTGTATATGTCGATGATATATTAATAGCATCAGAAACATTAGAAGACCATAGAAAACATTTAAAAGAATTTGTAAATGTCTGTATAAAAGAAGGTATAGTATTATCAGAAAGAAAAGCAGTTATAGAACATAAGAAAATAGAATTTTTGGGAATGATTGTTGATAAACAAGGAATTAGGCTCCAGAGCCATATAGGTAAGAAGATTAGTGAATTTCCAGATAAATTAGATAATAAACAACAAATACAGAGATTTTTAGGATGTTTAAATTATGCTGAAGGTTTTATAAAAGACCTAGCAAAAAAGAGAAATACACTACAAAGATTACTTAGAAAAAATAATACTAGAGGTTGGAATATAGAACATACTGAAACCGTTAAAATCTTGAAAGAAGAATGTAAAAATCTCCCTTCACTTAGATTACCTGAAGAAACTGATAAGTTAGTAATACAAACTGACGCATCAGATTTACATTGGGGAGCTGTACTCAAAACTGACATAAATGAAATTTGTAGATACACCAGTGGTACATTTAATCAAGCACAAGTTAATTATCCCGTGCATGAAAAAGAACTTCTTGCGATTTATAAAGGAATTAAAAAATTTTCCTTATTTTTATTACAAAAACCATTTATTGTAGAAACAGATAATTCTCAAGTAAGCTCTTTAATTACTAAAATCTTACCAAATGAGCCACAATATAGAAGGTTACATAGATGGCAAGCATATTTGTCATATTATAATTTCAAGATTATACATATCAAAGGTATTAATAATTTTCTTGCAGATTTCTTAAGCAGGAACATAGAATTTGGAAATGGATCCTGAAATAGATAAGATCCTATCCGGAATAGATGCCGAACTACATAGACTGGCAACAGAAGCATCAGACATCATCGATGCAATAAATTACATAAAAGAGGCTTCAGAGGCCCATGAGAAAGGAGTACATTTGTTAGTGTCACAAATAAATGACCTGACAAAAGCACTCTCGATAAACAAAGGCCCAAATACTGTGCCAATAAACACCAGCCCTAAAGGTGAAGGAAGCAGTAGTGGACCCATCAGAGAAAATAAACCCATCTCTGATCCAGTCATAAACCCACAAAACATAATCTCCATAGAAGTTGAACCACAAAAATCAAGCCCTTCAAAAAAGTTCATCCAAATAAACAATCTCCAGATAGAAGAGGAGAAGGAACCTTTGAATTCAAAAGAAGAAGAATATAAACACTTTTCTACCTGGATTTATTCCACTTGTTCCCATAACAATAATATCCATAAAGCACCAGGAAAAAACATGTACCCCAGAATGATAGTGACTAAAGGAGCAGACCCACTCCTTGTAGGAAAGATAGCCCGTTACGGTTATCTCGACCTAATATATCCTGACATAGACCTCCGGGAAATAGCCAGCCTGGATGAATTATTAGTTAAAGAAATATCCAAGTTTGCCCAAAAAAGACCCATATACCTCAAATTCTACACCATAAGTCCAGAATACCATCAGAATGTAGAATACCAAGCATTTTATATGATCTCAGTGGGTCATATAACCAAAAATTTTCAGTTAGAAGTAGGAACCGAATATACCAATGTTCCTCAGATTACCAAAAACTGGATCCAAACCAGAAGAGCCCGTGGGATAAAAGCAATATGGAGCATAGCAAAGGATTTATACAAGAGAAATTTCAGAACCATCATCAGATTCCAAAATTACATCCTTGTCACAGCTGAAGACAGCCATACAGAGTCAAATATTCTACTGGAGCTAATCAATGAGATAAGCACAATGCGATTTCCCAGTAGCAGTAAAACTATGCAACAAGCTTATCAGCTTATGGAATCAAGCAATCAGACAAGATAAAAAGCAGAAGTAGATAAAGCATAAGAAGGCAGATGGCCGACAAGATAAAAAGCAGAAGCAGATAAAGCAGAAAATGGCCGACAAGATAAAAAGCAGAAGCAGATAAAGCAGAAGATGGCCGACAAGATAAAATATTCAAAGACACTTGTCAAGTGGAAGAAAGTTGTAATAATGTAGTGGCGCATGTAATAATGTACAAGCCCAATAATGAAAGGGCAATAAGGTAACTTTTGTAAAACAAAAAGTTTGGAAATAAAAAGAAAAAGGAGGACTGATCATTCAGTTTGGGATCATTCAAAAGAAAAACCTTTCTTTTGCACAGGGACAAGTTCTCTGAAATCATGAACTAAAATTTTCTTGAACTCTAAATCCAAAATCAATACATCACAGATCGAAAGACAAATAAACACCATAGAAAGGATAGAGAAACCTAGACCTATTCATCTTTTCTAAGCACAACGATCCGAGTCCACACAGTAAAGCCGTTTGTGTCCGCTGATCCCGTTGAGGCAAAGAGGCCGTTGAGGGAATATGGACAAGTAGGCTGCAGGTCACTGGAAAGAGATCGGAGAGCAAAGAAGGAAGAATATTATAGGTAATAATTCTTGAACCTTATCTGTTGGTGGAGTTTGTTGATGATCTCTGATGAGGAGATTTATGGAGGAGGAGTTTGATGTAACAAAAAGAAAAATTTTATCATGAATCACAAATTTTATTTAGGAGAATCGAAAGAATACATGATAGATCCTTCAACTAAAATAACATATAATGATTATATGATGATAATAGATTTTAATTTAGGATCATATAAACAAAGTTCTTATTTGTTCAGTAAAGAAGAACTAGTAAGAACAGAAGATTTACTAGCTGTTCAGTCTTTTAATTTACCTCAGGAAATTAAACGAAAAATTATAGAAAAAATTGAAACGGATAATCTAAAACATAATATTATATTATTAACAATAGACGAATATTTTGATGTTGCTAAAGCATATCTAGAACAACCAGATCATAGAGCTGAACCTTCATATATGCATGATTCGGAAAACGAAGAACTTAACTTATAAATTGAAAAGAATATACTGATAATTTAATTTTTATTTATTTATTTACCAAGTTGTCTTGCAAAATTCTTGTTTTTGATGCTGGCATTATAATTTGTTTTTCTGTTTATTTTTTTTTATTATTATTATTTTATTTATGTAATTTCTGAAAAAACCATAACCCACAAAATGGTATCAGAGCCAAAGTTATAAAGAAGAAATTTTACTTAAATATGAATCCAGTGGATAGTATAGTTAATAGTTTATATACACTAAAGGTCTTTGAAAAAGAAGAAATAAAAAATTTGATAATATCTCTCAAGACAGAAAAGAACAATATAAGAAATATAGAAATTGAATTAGTACAAGAATTAATGGAAAGGTCAATAGATACATTAGGAGGAGAAATTATCATAGAAAAGAAAATGAAATTCATAGACGAATTTATAAAATTTTTAGTAAATTGGTATAAAAAAGAAGCATTACCATGAATCAACAAGCTTTTAACACAATACATCGAGTAACAGAAGCCTTAGACCAAATGAATTTAGGTTGGGAAAATATGTTAACAGTCTATATAAATCTAATAGCTCAAAAAATAGAATTATTAGAGGAAGAAAGGATAGGAATAATAAATTTAGGACCAGAAATTTATTCTGAATTTATAGATTTATATGAATTTGATAATCATATACTATTGTTTAAAGAAATAAAAAGAACATTAGTGAGAATAAAGAATAGGTGTATAATCGATCAAAGAAGGCAAGTAAATCCATAAATAACAAAAGCATTAAAACTAAGGACTGGAATGAAATTTATCCACTAGAAAAAGACAAAGAATGTACGTTACATTTAACGTAACGTAGAGGAACCC

zf-CCHC Zinc knuckle (coat protein) e-value 8.5e-06

MP Movement protein e-value 3.9e-31

Gag-asp-protease Aspartyl protease e-value 0.089

RVP Retroviral aspartyl protease e-value 0.014

RVT_1 Reverse transcriptase e-value 1e-27

RT-RNAseH_2 RNAseH e-value 2.3e-21

>ORF1

MDNTLDNLTEKFSDINLSDLEDFEKEYQIKMNVAGGPEMKPNVAEPSHRHMPKKKHSKPFYEYIPTIKNTSWKKEFIPNQDILTPISNSGAFLNLDCKIDPRKTLAEWGIQMKLFFTYNGTNDDWNVTTDEEKIDIFVDILLTSFTGNIFNWWRGLSEDTQLLIKDSTKIAFRRSNALGIERIVEYIASEFLGEDWLENSEREANNEKQRARLNLLNLTICNMCFIKEYTCEFAKYYYEQYISLEDQEVYKNLYYSKLPYPWNSYFINEYAKDSTSNRLPDTLGARIRFLNIRLSDICIQRNLIKKSKNITTICCEKTEMPTQWGCSVPYKRKRKFYKKEKRYKKYAKFKRKYRKPGRSYYKRKYKSQYKSQTKQLDKTKCKCWNCGEIGHISTDCKKKKVRILQKDFDTIQEELEEINNLTDYEGEEIYVIKDSDDESE

>ORF2

MMNQNKKDIVETEEIEDIEEIEREENKSGYCLVATFNKEKYNKLLKLNVEKPEYGIVQKLRDLFKRKRIVLHEYYEQEKAVTVTQAEGNIRFSLINKRTIDLALRKVKNETTREKIQYVYLAEIQILIKSLFKEGIDSPVVLSIHDQRFHDPSIGHLGTVEGNLCYTKLLFTCHPRYCVHIKDKNIDETLSLHFKLLKKNLMKEGNRLMTIHYSALYSFCNSNYGEIYGNKPYIEINKDCEDIATLIEPIMQDYKIPITYELNIDDKKQFLNENNENSIIPISFAGRTLLRGSSSKRLSTDNQITNTHNLALTKSSRNNNVVRIFGQYFNGIEYTLRLPILIDTGTSHNYMNHTKNKGLLIKEKDIPYEYTDFNGNKHICKQEVKVPLIIEGIRIIVSCYIDSFMTTDPEKHIVLGNSFLNSLEYYKIKKHQITLRIEGQKIICETC

>ORF3

MLRQAYIKYPMSVYIQINIVEKDLDTCALIDTGSEITIMKTFLSSQWKVNGQIKLIGITGDKQHINQSLLNCEILVGSKVVRINQVFQYNQMDCDILLGNDFIQQFQYYQQTTYMITLKTPCNHILRIPREFKPYRVQPAQRGDQVIYEKHYLISKGQSYNIRLETIKSQLEKVYKENPLALWRADHPRAKIELIENRIIRLKPMMYTEEDREEFKVQIKELLNLHLIRHSNSPHNSPAFMVRKRAEQVRGKARMVINYKELNKYTKFDGYFLPNKEVLINLVKNKTYYSKFDCKSGFWQIKMEENSIPYTAFSTPQGHYEWIVMPFGLKNAPQIFQSRMDKVFKDYKYIIVYVDDILIASETLEDHRKHLKEFVNVCIKEGIVLSERKAVIEHKKIEFLGMIVDKQGIRLQSHIGKKISEFPDKLDNKQQIQRFLGCLNYAEGFIKDLAKKRNTLQRLLRKNNTRGWNIEHTETVKILKEECKNLPSLRLPEETDKLVIQTDASDLHWGAVLKTDINEICRYTSGTFNQAQVNYPVHEKELLAIYKGIKKFSLFLLQKPFIVETDNSQVSSLITKILPNEPQYRRLHRWQAYLSYYNFKIIHIKGINNFLADFLSRNIEFGNGS

>ORF4

MDPEIDKILSGIDAELHRLATEASDIIDAINYIKEASEAHEKGVHLLVSQINDLTKALSINKGPNTVPINTSPKGEGSSSGPIRENKPISDPVINPQNIISIEVEPQKSSPSKKFIQINNLQIEEEKEPLNSKEEEYKHFSTWIYSTCSHNNNIHKAPGKNMYPRMIVTKGADPLLVGKIARYGYLDLIYPDIDLREIASLDELLVKEISKFAQKRPIYLKFYTISPEYHQNVEYQAFYMISVGHITKNFQLEVGTEYTNVPQITKNWIQTRRARGIKAIWSIAKDLYKRNFRTIIRFQNYILVTAEDSHTESNILLELINEISTMRFPSSSKTMQQAYQLMESSNQTR

>CitRet-071; *Citrus reticulata*; Scaffold_85909 (5566899-5565997rev); https://www.citrusgenomedb.org/;

ACCCCTATATTTTATGGAAACTACATTAATCGAATTAAAAGAAATACTTTTAAATCTTAAAGAGGAAATGAGAGAAATAAAACAGGAAATAGGAAAAATAAAAATAGAATTATCAACAATTCAGATAAGTCGAAAGAAAAATAAAAAAGTTGAGAAACTTGAAATAATAAAATCAGGAATAATAGGGACAAGCATTGAAAAACATATGACAAAACCTCAACCAGGAAGTTATGATGACTGGTTTATAAAAACCCAGTATCCAAAAATATTAGAGGAATCAGAAAAGTTAAAAGCAGAATTAAAAAGAGAAGAAAAGGGAAAAGAAAAATTAGAGATAAAAGAAAATACACCTAGTGACACAGAATAATGGAAAATACATTAGAAGAATTAACTGAAAAATTTAGTGATATTAACCTTAGTGATTCAGAAGAATTTGAAAAAGAATATCAAATAAAGATGAATGTTGCTGGAGGTCCAGAATTAAAACCTGATATTGGAGAACCTAGCCATAGATATGAAACAAATAAAAAGAAAAATAGACCTTTTTATGAATACTATCCGCCCGTTAAAAATACCCCTTGGAAAAAAGATTACCAACCTAATAATGACCATTTGATGCCAATAAGCAATGCAGGTACATTTTTAGACTTAGATTGTAAAATAGACCCCCGAAAAGCTTTAGTAGAATGGGGAATGCAAATGAAATTATTTTTTATATTCAATGGATCCAAAGATGATTGGAATGTAGATACTGAAGAAGAAAAAATAAATATATTAACAGATATTCTAATAGCAAGTTTTACAGGAAATGTATTTAATTGGTGGAAAGGATTAAGTGACGATACACAAAAATTAATAAAAGACTCAACCAAAATAGCATTAGGAAAAGATAAAGGATTAGGAATAGAGAGAATAATCGAATACATTGCTAGTGAATTCTTAGGAGAAGATTGGTTACAAAATACAGAAAATGAAGCAAAATATGATAAATTAAATGCTAGAATGAAATTAATAAATCTGACAATCTGTAATATGTGTTTTGTAAAAGAATATACATGTGAATTTAGTAAATATTATTATACACAATTCATGAGTCATGAAGACCAAAACATTTATAAAAATTTGTATTATTCTAAATTACCTTATCCATGGAATGGATATTTCATAAATGAATATGAGAAATATACTAATAAAAGTGATAGTAGAATCCCAGATACATTAGGATCTAGGATCAGATTTTTAAATACAAGATTAAGTGAAATTTGTATCCAAAGAAGTTTAATAAAAAAGAGTAAAAATATAACAAAAATATGTTGTGAAAAGACAGAAATGCCTACCCAATGGGGATGTTATGTACCTCATAAAAAACGTAGAAAATCTTATAATAAAGAAAAGAGATATAAAAAATATAATAATTTTAAGAAAAAATATAAAAAATCAGGAAGAAAATATTATAAAAGAAAATATAAAAACCAGAGAAAAATATTAGATAAGTCCAAATGTAAATGTTGGAATTGTGGAGAAATAGGGCATATAAGTACAGATTGTACAAGAAAGAAAAAAGTTAAACTCTTAAAAGAAGATTTTGAAAATATAGAAAGTGACTTAGAGGAAATAAATAACTTATCTGACTATATAGGAGAGGAAATTTATATGGCTGAAGAATCTGAAAATGAATCAGAATAAAGAAGAGTTATTAGATAATGAAAAAATAGAAGAGATAGAAGAAATAGAAAAAGAAGAAAATAAAAGTGGATACAGCCTTGTTGCTACTTTTAACAAAGAAAAATATAATAAACTTTTAAAATTAAATATGGAAAAACCAGAATATGGAATAATCCATAGATTTAGAGATATGTTTAAAAGAAAACGAATAATATTACATGAATATTATGAACAAGAAAAAGCTGTGACAATTACACAAGCTGAAGGAAACATTAGATTTAGTTTAATAAATAAAAGATCCATAGACTTAGCACTGAGAAAAATAAAAAATGAGTCAACTAAAGAAAAAATTCAATATGTATATCTCGCTGAGATACAAATCTTAATAAAATCTCTTTTTAAAGAAGGAATAGATAGCCCAATAGTTTTATCATTGCATGATCAAAGATTTACAGATCCTAAGATAGGACATCTTGGAACAATAGAAGGAAATTTATGTTATACTAAATTATTATTTACTTGTCACCCTAGATATTGTGTACACATTAAAGATAAAAACATAGACGAAACATTAAGCTTACATTTTAAGTTATTAAAGAAAAATTTAATGAAAGAAGGTAATAGAATAATGACAATACATTACTCAGCTTTATATAGTTTCTGTAATTCAAATTACGGAGAAATATATGGTAATAAACCTTACATCGAGATAAATAAAGATTGTGAAGATATCATAACATTTATAGAACCAGTAATCCAAGATTACAAAATCCCTATAAATTATGAATTAAATATCGATGATAAAAAACAATTTTTAGATAATAGTAATGAAAACTCCATTATTCCTATAAGCTTTGCTGGAAGAACCTTAATAAGAGGAAGTTCTTCTAGAAGATTAAACACTGATAAGCAAATAATTAAATCGGTACCAACAAATAATGTGGTAAAAATACTTGGACAATACTTTAATGGAATAGATTATACAACAAAAGTACCTATACTATTAGACACTGGCACAAGCCATAATTATATGAATCACACAAAAAATAAAGGACTCTTAGTAAAAGAAAAGGAAGTTCCGTATGAGTATACTGACTTCAATGGGAATAAACATATTTGTAAACAAGAAGTTAAAGTCCCTATAATAATAGAAGGACTTAGAATAATAGTAGCCTGCTATATAGATTCATTCATGACTACAGACCCTGAAAAACATATAGTACTAGGAAATAGTTTTTTAAATGATTTAGAATATTACAAAATTGAACCTAATAAGATAACTTTCTTAATCCAAGGCCAAACAATAATTTGTTACACATGTTAAAACAAGCTTATGTAAAATACCCTATGTCTGTATATATACAAATAAACATAACCGAAAAAGATATAGATACTTGTGCTTTGTTAGACACTGGTAGTGAAATAACTATTATGAAATCCTTTTTATCTAATAAATGGGAATCAAATGGAAAATTAAAAATTATTGGTATTACAGGAGATAAACAACATGTCAATCAATCTATTTTAAATTTTGAAATCTTACTAGGAAGTAAAGTAGTACGTATTAACCAAATATTCCAATACAATAATATGGATTGTGATATCCTTTTAGGAAATGACTTCATCCAACAGTTCCAATATTATCAACAAACACCTTATATGATCACTTTAAAAACCCCTTGTAATCATATTCTCCGCATACCTCGCGAATTTAAACCCTACAGAGTTCAGCCAGCCCAGCGTGGTGATAAGTATATTTATGAAAAACATTATTTAATACAAAAAGGACAAAGTTATAATATACAACTAGAAACAATAAAATCACAGCTTGAGCAAGTTTATAAAGAGAATCCCTTAGCATTATGGAAACCAGATCATCCTAGAGCAAAAATTGAATTAATTGAAAATAGGATTATAAGACTTAAACCTATGATGTATACAGAAGAAGATAGAGAAGAATTTAAAATTCAAATAAAAGAATTATTAAATTTAAAACTTATTAGAAGTAGTAATAGCCCGCATAATAGTCCAGCTTTTATGGTAAGGAAAAGAGCAGAACAGGTTAGAGGAAAAGCGAGAATGGTAATAAACTACAAAGAACTAAATAAATATACAAAATTTGATGGATATTTTCTCCCAAATAAGGAAGTACTAATAAATCTTGTAAAAAATAAAACATATTATTCAAAATTTGATTGTAAGTCAGGATTTTGGCAAATAAAAATGGAAAATGATAGTATCCCCTACACAGCCTTTAGTACCCCACAGGGTCATTATGAATGGTTAGTAATGCCCTTTGGATTAAAAAATGCTCCACAAATTTTTCAAAGTAGAATGGATAAAATATTTAAAGACTACAATTACATTATAGTCTATGTTGATGATATATTGATAGCTTCAGAAACAATAGAAGAACATAGACACCATTTGAAAGAATTTGCTAATACATGTATTAGAGAAGGGATAGTACTGTCTAGTAAAAAAGCAGTAATAGAGCATAAGAAAATAGAATTTTTAGGAATGATACTTGATAAACAAGGAATCAAACTCCAGAGCCATATAGGAAGAAAAATAATTGAGTTTCCAGATAAATTAGAAAATAAACAACAAATACAAAAATTCTTGGGATGTTTGAATTATGCAGAAGGATTTATAAAAGACCTATCAAAAAAGAGAAATATACTCCAAAAATTATTAAGAAAAAATAATACTAGGGGATGGAATGAGGAACATACAGAAACTGTAAAGAATTTAAAAGAAGAATGTAAAAACCTTCCGACTTTAAGACTACCAGATGAAAAAGATAGATTAGTAATACAAACAGATGCGTCTGACTTATATTGGGGAGCAATACTTAAAACAGATATAAATGAAATTTGTCGATACACTAGTGGAACGTTTAACCAAGCTCAGGTTAATTATCCTGTACATGAAAAAGAACTCCTTGCAATATACAAAGGGATTAAAAAATTCTCATTATTTTTGCTCCAAAAACAATTTACTGTAGAAACTGATAATTCTCAGGTAAACTCCCTTATTAATAGAATTTTACCAAATGAACCTCAGTATAGAAGATTGCATAGATGGCAAGTTTATTTGTCATATTATAATTTCAAAATTGTCCATATAAAAGGAATTAACAATTTTCTTGCAGATTTCCTAAGCAGGAACATAGAATTTGGAGAATGAATCCCGAAGTTAATAGAATCATCGGGAAAATAGAAGCTGAGGTACAAAGGCTCAAAACAGAAGCATCTGATATAATTGATGCAATAAACTATTTGAAAGAAGCTTCTGAGGCTCATGAAAAAGGAGTAAATTTGTTAGAGTCATACATAGACGACCTAACAAAAGCTCTCTCTATAAATACGAGCCCAATAAACATTAACCCAATAAATATCAGAAGCAGTAGTGGACCCTTCAGAGAAGATAAACCAATCTCTGACCCAGTTCCTAATAAAGCCATATCTATAGAAGTCGAGTCTCCAAAATCAAAACCTCCAAAAAAGTTCATAGAAATAAACAATCCTTATACAGAAGAAGAGGAGAAACCCCTGAATCCAAAAGAAGAAGAATATAAACAATTCTCCATATGGGTGTACTCCACTTGTTCCCACAAGAATACAATTTTCAAAGCACCAGGAAAAAATATGTATCCAAGAATGATCGCTACAAAAGGAGCAGACCCACTCCTTGTTGGAAAGATAGCAAAATACGGGTATTTAGATCTAGTTTATCCAGATATAAATCTTCAGGAAATAGCCAGCCTTGATGATTTGTTAATAAAAGAAGTATCAAGGTTCGCCCAAAAAAGGCCCATATACCTAAAATTCTACACCATAAGCCCAGAATACTATCAGAACGTAGAATATCAAGCCTTTTATATGATCTCTATAGGTCATCTCACTAAGAATTTCAAGGTTGAAGTTGGGACAGACTACACAAATGTTCCACATATAAATGAACCTTGGATCAGAACACGAAGAGCCAGAGGAATAAAAGCCATATGGAGCATAGCCAAGGATCTATACAAAAGAAATTTCAGAACCATCATCAAATTCCAGAACTACATTCTCATAACATCTGAAGACAGTCATACACCATCAAATCTGTTGTTAGACTTTATCAATGAAATAAGCACTATGCGTTTTCCCAGTAGCAGTAAAACTATGGAACAAGCTTTTCAGCTCATGGAAGTGGCAGACAACATAAAGAAGATGCCCGATAAGAAGTGGCAGACATCATAAAGAAGATGCCCGACAAAGTAAAGTAAATGTCAAAAAGCAAAAAGAAATATGCTAAAATTAAAAGTAAGTGATATGTAACGTCAGCAGTGACGTAAGAAGCCCAATCATATAAGGGCAATAATGTAATAAAGCATGGAGGAGAATGTAAAAGGCCCAAAGATAGAAGGGCAAGAATGTAAACCTTTTTTATATAAATAAAAGAAAAAAAGAAAGAAGGAAGGCAGAGCTTCCATTACGAAATCTTTTCCAAAAACTCTCTCAAAAATGAAGAACTAAACAACTCTTCAGCTCAAAATCCAGAAATCAAGCCATCAGAAATCAACAAAACAACACCATAGAAAGGAAAGAGAAACCTAAATTTGTTCAAAATTTCCGAGCATAACGACCAAAATCCATACAGTAAGGTCGCTCGAGTCCGCTGATCCCGTTGAGGCAAGGAGGCCGTTGAGGGAATAAGGACAACCAGGCTGAAGGTCACTGGAAAAGGATCGGAGGCAGAGAAATAAGAACAACTCTAGGTAATAATTCTCAAACCTTATCTGTTGGTGGAAGTTTGTTGATGATTGATGATGAGGAGATTTATGGAGGAAGAGCTGCTAATTTAAACATGAATAACGATATTTTACTTGGGAATTCCAAAGAATATATGATAAACTCTACCACTAGAATAACATATAATGACTATATGATGATAATAGACTTTAACTTGGGATCTTTTAAACAAAGTTCATATCTTTTAAGTAAATCAGAGCTAGTTAGAACAGAAGATCTACAAGCAGTATATACTCTCAATTTACCAAAAGAAATAAAAAGAAAAATTATAAACCATATCGAAACAAATAATTTAAAACATAATATAATTATGTTAACAATAGAGCAAGGAATTGACATAGCTTTGGCATATATAGAACATGAAAGTCGAGAAATTGGACCATCATATATAAATTACGTTGAAAATTCGGATAACGAAGAACTTGAACTATAAGAATCTAGTAAAATAAAACAAATAAACTGGTTGTAAGGTATATCGGCAAGCAACCCATGACGTAAATTGGATAGACGATCCCTATTGGGCGTTAGTATCCTCTGCACTTTGGGATAACAAGCCACGTACTTGTAATCAGGATATTTTATAAAAATAGATACTCAAAGGGAAATTAGATACTATTTTAGTATAATAAATAATAATATATACTTTATACAGATCGAATTATTTTATGATATGACTAATAATCCTGCATATATTTTAGAAACTAATAATAATTATTATGATTTTTATTTTCCTAGTTTTCTTAATAATATACCTAGGATTATAGAAGGAGAACAAGTTACTGCCTTATATGTAAGAAATAACAGTAATAGTTATGATATGTATACCTTATAGAACAAAAATTTTTGTTTTATGTTTTTAGTTTTCTGTAATTCTTTATTCTATTATTTTTTTATTTATTTTAGTTATATTTTCATTCTTTATCTTTTTGGTTTTATTTGTTTTTTTTTAGCACACAGTGCAAGATGGTATCAGAGCCATAAGAACAAAATTATGGACCCAATACAGTCAATAGTTAATAGTCTGTATACATTAAAGGTATATAATAAAGAGGAAATAAAGAATTTGATAAAAGCTTTAAAAACTGATAAAAATAATATACAAAATATAGAAATGGAATTAACTGAAGAACTTATAGAAAGGAATATAGATACCACGGGAGGAGAAATATTGATAGAAAAGAAAATGAAATTTATTGATAAATTTATACAATTTTTAATATATTGGTATAAAACTGAAACATTATGAACCAAGCAGTTAACACAATACAGGAATTAATAAACAGTACGGAACATATGAATTTAGGATGGGAAAATATAGTAAGTACATATATAAATTTAATAAATCAGAAAATAGATTTATTAGAGCAAGAAAGAATAGGATTAATAAATTTAGGACCAGAAATATATTCTGAATTTATAGATCTATATGAGTTTGATAACCACTTGTTGTTATTAAAACAAATAAATACAACTCTGGAAAGAATAAAAAATAGATGTAGAAGAGATGGAAGGAGACAAATATTCCCTCAATTATAGAAAATAAAGAAACATCAAGGGACTCAAATGAAATCTACCAAAAAGAATAAAACAAAAAACGTAAGGTACATTGATCGCAACTTAGGCGCTCC

zf-CCHC Zinc knuckle (coat protein) e-value 6.7e-06

MP Movement protein e-value 1.5e-31

Gag-asp-protease Aspartyl protease e-value 0.06

RVP Retroviral aspartyl protease e-value 0.0035

RVT_1 Reverse transcriptase e-value 3.9e-28

RT-RNAseH_2 RNAseH e-value 9.4e-24

>ORF1

MENTLEELTEKFSDINLSDSEEFEKEYQIKMNVAGGPELKPDIGEPSHRYETNKKKNRPFYEYYPPVKNTPWKKDYQPNNDHLMPISNAGTFLDLDCKIDPRKALVEWGMQMKLFFIFNGSKDDWNVDTEEEKINILTDILIASFTGNVFNWWKGLSDDTQKLIKDSTKIALGKDKGLGIERIIEYIASEFLGEDWLQNTENEAKYDKLNARMKLINLTICNMCFVKEYTCEFSKYYYTQFMSHEDQNIYKNLYYSKLPYPWNGYFINEYEKYTNKSDSRIPDTLGSRIRFLNTRLSEICIQRSLIKKSKNITKICCEKTEMPTQWGCYVPHKKRRKSYNKEKRYKKYNNFKKKYKKSGRKYYKRKYKNQRKILDKSKCKCWNCGEIGHISTDCTRKKKVKLLKEDFENIESDLEEINNLSDYIGEEIYMAEESENESE

>ORF2

MNQNKEELLDNEKIEEIEEIEKEENKSGYSLVATFNKEKYNKLLKLNMEKPEYGIIHRFRDMFKRKRIILHEYYEQEKAVTITQAEGNIRFSLINKRSIDLALRKIKNESTKEKIQYVYLAEIQILIKSLFKEGIDSPIVLSLHDQRFTDPKIGHLGTIEGNLCYTKLLFTCHPRYCVHIKDKNIDETLSLHFKLLKKNLMKEGNRIMTIHYSALYSFCNSNYGEIYGNKPYIEINKDCEDIITFIEPVIQDYKIPINYELNIDDKKQFLDNSNENSIIPISFAGRTLIRGSSSRRLNTDKQIIKSVPTNNVVKILGQYFNGIDYTTKVPILLDTGTSHNYMNHTKNKGLLVKEKEVPYEYTDFNGNKHICKQEVKVPIIIEGLRIIVACYIDSFMTTDPEKHIVLGNSFLNDLEYYKIEPNKITFLIQGQTIICYTC

>ORF3

MLKQAYVKYPMSVYIQINITEKDIDTCALLDTGSEITIMKSFLSNKWESNGKLKIIGITGDKQHVNQSILNFEILLGSKVVRINQIFQYNNMDCDILLGNDFIQQFQYYQQTPYMITLKTPCNHILRIPREFKPYRVQPAQRGDKYIYEKHYLIQKGQSYNIQLETIKSQLEQVYKENPLALWKPDHPRAKIELIENRIIRLKPMMYTEEDREEFKIQIKELLNLKLIRSSNSPHNSPAFMVRKRAEQVRGKARMVINYKELNKYTKFDGYFLPNKEVLINLVKNKTYYSKFDCKSGFWQIKMENDSIPYTAFSTPQGHYEWLVMPFGLKNAPQIFQSRMDKIFKDYNYIIVYVDDILIASETIEEHRHHLKEFANTCIREGIVLSSKKAVIEHKKIEFLGMILDKQGIKLQSHIGRKIIEFPDKLENKQQIQKFLGCLNYAEGFIKDLSKKRNILQKLLRKNNTRGWNEEHTETVKNLKEECKNLPTLRLPDEKDRLVIQTDASDLYWGAILKTDINEICRYTSGTFNQAQVNYPVHEKELLAIYKGIKKFSLFLLQKQFTVETDNSQVNSLINRILPNEPQYRRLHRWQVYLSYYNFKIVHIKGINNFLADFLSRNIEFGE

>ORF4

MNPEVNRIIGKIEAEVQRLKTEASDIIDAINYLKEASEAHEKGVNLLESYIDDLTKALSINTSPININPINIRSSSGPFREDKPISDPVPNKAISIEVESPKSKPPKKFIEINNPYTEEEEKPLNPKEEEYKQFSIWVYSTCSHKNTIFKAPGKNMYPRMIATKGADPLLVGKIAKYGYLDLVYPDINLQEIASLDDLLIKEVSRFAQKRPIYLKFYTISPEYYQNVEYQAFYMISIGHLTKNFKVEVGTDYTNVPHINEPWIRTRRARGIKAIWSIAKDLYKRNFRTIIKFQNYILITSEDSHTPSNLLLDFINEISTMRFPSSSKTMEQAFQLMEVADNIKKMPDKKWQTS

>ForHin-314; *Fortunella hindsii*; tig00040100 (7454768-7462919dir); https://www.citrusgenomedb.org/;

ACCCGTATATTTTATGGAAACTACATTAATTGAACTAAAAGAGATACTTCTAAATCTTAAAGAAGAAATAAGAGAAATTAAACAAGAAATAGGAAAAATAAAAATAGAATTGTCAACAATCCAAATAAGTCGAAAGAAAAATAAAAAAGTCGAGAGACTTGAAATAATAAAATCAGGAATAATAGGAACAAACATTGAAAAAGATATAACAAAACCTCAACCAGGAAGTTATGATGACTGGTTTATAAAAACCCAATACCCAAAAATATTAGAGGAGTCAGAAAAATTAAAAGCAGAATTAAAAAGAGAAGAAAAGGGAAAAGAAAAATTAGAAATAAAGGAAAGCATACTTAGTGATACAGAATAATGGAAAATACATTAGAAGAATTAACAGAAAAATTTAGTGATATTAACCTTAGTGATTTAGAAGAATTTGAAAAAGAATATCAAATAAAAATGAATGTTGCTGGAGGTCCAGAATTAAAACCTGATATAGGAGGACCAAGCCATAGATATGAAACAAATAAGAAGAAAAATAGACCATTTTATGAATACTGTCCACCCGTCAAAAACACCACTTGGAAAAAAGAATATCAACCTAATAATGACCACTTGATGCCAATAAGCAATGCAGGAACGTTTTTAGACTTAGATTGTAAAATAGACCCTCGAAAAGCTTTAGTAGAATGGGGAATGCAAATGAAATTATTTTTTATATTCAATGGATCCAAAGATGATTGGAATGTAGATACAGAAGAAGAAAAAATAAATATACTTACAGATATTCTAATAGCAAGTTTTACAGGAAATGTATTTAATTGGTGGAAAGGATTAAGTGAAGATACGCAAAAATTAATAAAAGATTCAACCAAAATAGCATTAGGAAGGGATAAAGGGCTAGGAATTGAAAGAATAATTGAATATATTGCTAATGAATTTTTAGGAGAAGATTGGTTACAAAACACAGAAAAAGAAGCAAAACATGATAAATTAAATGCCAGAATGAAATTAATAAATTTAACAATATGTAATATGTGTTATGTAAAAGAATATACATGTGAATTTAGTAAATATTATTACACACAATTCATGAGCCATGAAGATCAAAATATTTATAAAAATTTATACTATTCTAAGTTACCATATCCATGGAATGGATGTTTCATAAATGAATATGAAAAATATACAAGTAATAATGAAAATAGGCTCCCAGATACATTAGGATCTAGGATCAGATTTTTGAATACGAGGTTAAGCGAAATATGTATCCAAAGAAGTTTAATAAAAAAGAGTAAAAATATAACAAAGATATGTTGTGAAAAGACAGAAATGCCTACTCAATGGGGATGTTATGTACCTCATAAAAAACGTAGAAAAATTTACCATAAAGAGAAGAGATATAAAAAGTATAACAATTTTAAGAAAAAATATAAGAAATCAGGAAGAAAATATTATAAAAGAAGATATAAAAACCAAAGGAAAATATTAGATAAATCTAAATGCAAATGTTGGAATTGCGGAGAAACAGGACATATAAGTACAGATTGTATAAAGAAGAAAAAGGTTAAACTTTTAAAAGAAGATTTTGAAAACATAGAAAGTGACTTAGAAGAAATAAATAATTTATCCGACTACATAGGAGAAGAGGTTTATATGGCAGAAGAATCTGAAAATGAATCAGAATAAAGAAGAAACATTAGATATTGAAGAAATAGAAGAAATAGAAAGAGAAGAAGATAAAAGTGGATATAGCCTTGTTGCTACATTCAATAAAGAAAAATACAATAAACTTTTAAAATTAAAAATGGAAAAACCAGAATATGGAATAATCCATAAATTTAGAGATATGTTTAAAAGAAAACAAATAATATTACATGAATATTATGAACAAGAAAAAGCTGTAACAGTTACACAAGCTGAAGGAAATATTAGGTTCAGTTTAATAAATAAAAGATCAATAGAATTAGCACTAAGAAAAATAAAAAGTGAGTCAATAAAAGAAAAAATTCAATATGTATATTTAGCTGAAATACAAATTCTAATAAAATCTCTTTTTAAAGAAGGAATAGATAGTCCTATAGTCTTATCACTACATGATCAAAGATTTACAGATCCTAGTATAGGACATCTTGGAACAATAGAAGGAAATTTATGTTATACTAAATTATTATTTACTTGCCACCCTAGATATTGTGTTCACATTAAAGATAAAAATATAGATGAAACATTAAGCTTACATTTTAAGTTATTAAAGAAAAATTTAATGAAAGAAGGTAATAGAATAATGACAATACATTACTCAGCTTTATATAGTTTCTGTAATTCAAATTACGGAGAAATATATGGTAATAAACCTTACATAGAGATAAATAAAGATTGTGAAGACATTGTAACATTTATAGAACCAGTAATCACAGATTATAAAATGCCTATTAATTATGAATTGAACATAGATGATAAAAAACAATTTCTAGATAATGGTAATGAGAACTCCATTATCCCAATAAGTTTTGCTGGAAGAACTTTAGTAAGAGGAAGCTCTTCTAGGAGATTAAACACTAATAGACAAATAATCAAATCAGTACCAACAAATAATGTGGTAAAAATACTTGGACAATACTTTAATGGAATAGATTATACAACAAAAATACCTATACTATTAGACACAGGCACAAGCCATAATTATATGAATCACACAAAAAATAAAGGACTCTTAGTAAAAGAAAAAGAAGTTCCATATGAATATATTGATTTCAATGGAAATAAACATATTTGCAAACAAGAAGTTAAAGTCCCTATAATAATAGAAGGACTTAGAATGATAGTAGCTTGTTACATAGATTCATTCATGACTACAGACCCTGAAAAACATATAATATTAGGAAATAGTTTTTTAAATGATCTAGAATATTACAAGATTGAACCAAATAAAGTAACTTTCTTAATAGGAGATCAAACAATAACTTGTTACACATGTTAAAACAGGCTTACATAAAATATCCTATGTCTGTATATATTCAAATAAATATAACGGAGAAAGATATAGATACTTGTGCTCTAATAGACACTGGTAGTGAAATAACTATTATGAAATCCTTCTTATCCAATAAATGGGAAGCCAATGGAAAATTAAAAATCATTAGAATTACAGGAGATAAACAACATATAAATCAATCTATCCTAAACTTTGAAATATTACTCGGTAGTAAAATAGTACGAATTAACCAAATATTCCAATACAATGGTATGGATTGTGATATCCTTTTAGGTAATGATTTTATACAACAGTTTCAATATTACCAACAGACACCTTATATGATCACTTTAAAAACCCCTTGCAACCATATTATCCGTATCCCTCGTAAATTTAAACCTTACAGAGTTCAGCCAGCCCAGCGTGGTGATAAGTATATTTATGAAAAACATTATTTAATACAGAAAGGACAGAGTTATAATATACAATTAGAAACAATAAAATCACAGCTTGAGCAAGTATATAAAGAAAACCCGTTAGCATTATGGAAACCAGATCATCCTAAAGCAAAAATAGAATTAATTGAAAATAGGATTATAAGACTAAAACCTATGATGTACACAGAAGAAGATAGAGAAGAATTTAAAATCCAAATAAAAGAATTATTAAATTTAAAACTTATTAGAAACAGTAATAGTCCACATAATAGTCCAGCTTTTATGGTTAGAAAAAGAGCTGAACAAGTTAGAGGAAAAGCAAGAATGGTAATAAACTACAAAGAGCTAAATAAGTATACAAAATTTGATGGATATTTTCTCCCTAATAAAGAAGTACTAATAAATCTTGTAAAAAATAAAACATATTACTCAAAATTTGACTGTAAGTCAGGCTTTTGGCAAATAAAAATGGAGAATGATAGTATTCCCTATACAGCCTTTAGTACACCACAAGGTCATTATGAATGGCTAGTAATGCCCTTTGGATTAAAAAATGCCCCACAAATTTTCCAAAGTAGAATGGATAAAATATTCAGGAACTATAAATATATTATAGTTTATGTTGATGATATACTGATAGCTTCAGAAACATTAGAGGAACACAGACACCATTTGAAAGAATTTGCTAATACATGTATTCGAGAAGGAATAGTACTATCTAGTAAAAAAGCAGTAATAGAGCATAAGAAAATAGAATTTTTAGGAATGATACTTGATAAGCAAGGAATCAAACTCCAGAGTCACATAGGAAGAAAAATAATTGAGTTTCCAGATAAATTAGAAAATAAACAACAAATACAAAAATTCTTAGGATGTCTAAATTATGCAGAAGGATTTATAAAAGACTTATCAAAAAAGAGAAATACACTCCAAAAATTATTAAGGAAAAATAATACTAGAGGTTGGAATGAAGAACATACAGAAACTGTAAGAAGTTTAAAAGAAGAATGTAAAAACCTCCCAACTTTAAGATTACCAGATGAAAAAGACAGACTAGTTATACAAACCGATGCGTCTGATTTATATTGGGGAGCAATACTCAAGACTGATATAAATGAAATTTGTCGATATACTAGTGGAACGTTTAATCAAGCTCAGATTAATTATCCTGTACATGAAAAGGAACTCCTTGCAATATACAAAGGAATTAAAAAATTCTCATTATTTTTACTCCAAAAACAATTTATTGTAGAAACTGATAATTCCCAGGTAAATTCTCTTATCAATAAGATTTTACCAAATGAACCCCAGTATAGAAGATTGCATAGATTGCAAGTTTATTTGTCATATTATAATTTTAAAATTGTTCATATAAAAGGAATTAACAATTTCCTTGCAGATTTCCTAAGCAGGAACATAGAATTTGGAGAATGAATCCCGAAGTAAATAGAATCATCGGGAAAATAGAAGCTGAAGTGCAAAGGCTCAAAACAGAAGCATCTGATATAATTGATGCAATAAACTACCTAAAAGATGCCTCTGAGGCACATGAAAAAGGAGTAAATTTGTTAGAATCATACATAGACGATCTGACAAAGGCACTCTCTATAAACACAAGTCCAATAAACATTAACCCAATAAGTATAGGAAGCAGTAGTGGACCCTTCAGGGAAGATAAACCAATTCCTGACCAAGTTCCAAATAAAACCATATCCATAGAAGTCTGTTCACAAGAAGCCGGTTCACCAAAATCAGATCCTCCAAAAAAGTTCATAGAAATAAACAATCCTTATATAGAAGGAGACGAAAAACCCCTGAACCCAAAAGAAGAAGAATATAAACAATTTTGTACATGGGTATACTCCACTTGTTCCCACAATAATACAATTTTCAGAGCACCTGGAAGAAATATGTACCCCAGAATGATTGTTACAAAAGGAGCAGATCCACTCCTTGTTGGAAAGATAGCAAAATATGGGTATTTAGACTTGATATATCCAGATATACACCTTCAAGAAATAGCCAGTTTTGATGATTTACTAATAAAAGAAATATCAAGGTTTGCCCAAAAAAGGCCCATATACTTGAAATTCTACACCATAAGCCCAGAATACTATCAGAACGTAGAATATCAAGCCTTTTATATGATCTCTATAGGTCATCTTACTAAGAATTTCAAGGTTGAAGTTGGGACAGAATATACAAATGTTCCACATATAAATGAACCTTGGATCAGAACAAGAAGAGCCAGAGGTATAAAGGCTATATGGAGTATAGCCAAAGATCTGTACAAAAGAAACTTCAGAACCATCATCAAGTTCCAGAACTACATTCTCATAACCTCCGAAGACAGTCATACACCATCAAGTCTATTATTAGATTTCATCAATGAAATAAGCACTATGCGTTTTCCAAGCAGCAGTAAAACTATGGAGCAAGCTTATCAGCTCATGGAAGTGGCAGACAACATAAAGAAGCTGCCCGACAAGATAAGGCAGACAACATAAAGAAGATGCCAGACAAGATAAAAATGTAATAGTTGAGTGGCATGTAACGTAAGCAATGATGTAAGATGCCCAATCATAAAAGGGCAATAATGTAATAGTTGAGTGGCATGTAACGTAAGCAATGATGTAAGATGCCCAATCATAAAAGGGCAATAATGTAATCTTTGGTTATAAATACAAAGAAAAAAGAAGAAGCTCTCAGAGTTTTGAGAAAGAAGCATCAAAATCAAAATTTTTCTCTCAAAATGAATAACTAAACAACTCCTCAACTCTAAATCCAAAAAATCAAATCATCGGAAATCATAAACAAAACAACACCATAGAAAGGTAAGAAAAACCTAAACTTATTCAAAATTTCCAAGGCTTAGCGATCAGAGATCCATACAGAAAGGCCGATCTAGTCCGCTGATCCCGTTGAGGCAAGGAGGCCGTTGAGGGAATAAGGACAACCAGGCTGCAGGTCACTGGAGAAGGATCGAGGAGCAAGGAAACAAGGATAAGCTAGGTAATAATTTTCAAACTTTATCTATTGGTGGAAGTTTGTTGATGATTGATGATGAGAAGATTTATGGAGGAAGAGTTAATTTAAACATGAGTAATGTTATTCTGCTCGGAAACTCTAAAGAATATGTAATAAACCCTACTACTAAAATTACATATAATGATTATATGATGATAATAGATTTCAACCTAGGATCTTTTAAACAAAGTTCATATCTTGTAAGTAAAGAAGAACTAGTCAGAACAGAAGATCTGCAAGCAGTATATAATTTTAATTTACCAAAAGAAATAAAAAGAAAAATTATAAACCATATAGAAATAGATAATTTAAGGCATAATATAATTATGCTAACAGTAGAACAGGGAATTGATATAGCCTTAGCATACATAGAACATGAAAGTCGAGAAATTGGACCATCATATATAAATTACATAGAAAATTCAGATAACGAAGAACTTGAAATATAAGAATTTAAAATAAAATACATATACTGATTATAAGGTATATCGGCAAGCAACCCATAACGTAAATTGGATAGACGATCCCGATTGGGCGTTAGTATCCTCTGTATTTTGGGAAAACAAGCCACGTACCTATAATTAGGATACTTTGAAAACATAGGTACTCTAAGAGAAACCAGATATTATTTTAGTATAATAAATAATAATATATACTTTATACAAATAGAATTATTCTATGATCAGGCTAATGGCCCTGCATATATTTTAGAAACTAATCATAATTATTATGATTTCTATTTTCCTAATTTTCTCAATAATATACCTAGGATAATAGAAGGAGAACAAGTTATAGCTTTATATATAAGAAATAATGATAATACAGGTTATAATATGTATATCTTATAAAAATTCTCTATCTTTAGATTAAACAATTGTTATCTTATTGTATTTTTATATTCTGTTTTATTTTATTTACAATTTATATTTATATTTGTATTTTTTATTTTTGTTTACATTTTAGTTCATTAAGAACAAGATGGTATCAGAGCCATAAAAAGACTTAAAAACTACAATTATGGACCCAATACAAAAAATAGTAGATAGCTTATATACATTAAAAGTATATAATAAAGAAGAAATAAAAAATTTGGTAAAAGCATTAAAAACTGATAAAAATAATATTAGAAATATAGAACTTGAGTTGACTGAGGAACTCTTAGAAAGAAATATAGATACAACTGGAGGAGAAATATTGATAGAAAAGAAAATGAAATTTATTGATAAATTTATACAATTCTTAATATGTTGGTATAAAACTGAAACATTATGAATCAAGCTGTTCATACAATACAGGAGTTAGTAAATAGCATAGAACGTATGTTTTTAGGATGGGAAAATATAGTAAGTACATATATAGATTTAATAAATCAAAAAATAGACTTGTTAGAACAAGAAAGAATTGGATTAATAAATATAGGACCAGAAATATATTCTGAATTTATAGATCTATATGAATTTGATAATCATCTTTTGTTACTAAAAGAAATAAAAATAACTTTAGAAAGAATAAAAAACAGGTGTAGAAGAGATGATAGAAGACAAATACTTCCTCAACTATAGAAAATAAAGAAACATTGAAGGACTCAGATGATATTTACCAGAAAGAATAAGACAAAAAACGTAAGATACGTTGATCGTAACTTAGGTGCTCCC

zf-CCHC Zinc finger (coat protein) e-value 2.1e-05

MP Movement protein e-value 1.4e-30

Gag-asp-protease Aspartyl protease e-value 0.099

RVP Retroviral aspartyl protease e-value 0.096

RVT_1 Reverse transcriptase e-value 3.5e-28

RT-RNAseH_2 RNAseH e-value 4.6e-23

>lcl|ORF1

MENTLEELTEKFSDINLSDLEEFEKEYQIKMNVAGGPELKPDIGGPSHRYETNKKKNRPFYEYCPPVKNTTWKKEYQPNNDHLMPISNAGTFLDLDCKIDPRKALVEWGMQMKLFFIFNGSKDDWNVDTEEEKINILTDILIASFTGNVFNWWKGLSEDTQKLIKDSTKIALGRDKGLGIERIIEYIANEFLGEDWLQNTEKEAKHDKLNARMKLINLTICNMCYVKEYTCEFSKYYYTQFMSHEDQNIYKNLYYSKLPYPWNGCFINEYEKYTSNNENRLPDTLGSRIRFLNTRLSEICIQRSLIKKSKNITKICCEKTEMPTQWGCYVPHKKRRKIYHKEKRYKKYNNFKKKYKKSGRKYYKRRYKNQRKILDKSKCKCWNCGETGHISTDCIKKKKVKLLKEDFENIESDLEEINNLSDYIGEEVYMAEESENESE

>lcl|ORF4

MNQNKEETLDIEEIEEIEREEDKSGYSLVATFNKEKYNKLLKLKMEKPEYGIIHKFRDMFKRKQIILHEYYEQEKAVTVTQAEGNIRFSLINKRSIELALRKIKSESIKEKIQYVYLAEIQILIKSLFKEGIDSPIVLSLHDQRFTDPSIGHLGTIEGNLCYTKLLFTCHPRYCVHIKDKNIDETLSLHFKLLKKNLMKEGNRIMTIHYSALYSFCNSNYGEIYGNKPYIEINKDCEDIVTFIEPVITDYKMPINYELNIDDKKQFLDNGNENSIIPISFAGRTLVRGSSSRRLNTNRQIIKSVPTNNVVKILGQYFNGIDYTTKIPILLDTGTSHNYMNHTKNKGLLVKEKEVPYEYIDFNGNKHICKQEVKVPIIIEGLRMIVACYIDSFMTTDPEKHIILGNSFLNDLEYYKIEPNKVTFLIGDQTITCYTC

>lcl|ORF2

MLKQAYIKYPMSVYIQINITEKDIDTCALIDTGSEITIMKSFLSNKWEANGKLKIIRITGDKQHINQSILNFEILLGSKIVRINQIFQYNGMDCDILLGNDFIQQFQYYQQTPYMITLKTPCNHIIRIPRKFKPYRVQPAQRGDKYIYEKHYLIQKGQSYNIQLETIKSQLEQVYKENPLALWKPDHPKAKIELIENRIIRLKPMMYTEEDREEFKIQIKELLNLKLIRNSNSPHNSPAFMVRKRAEQVRGKARMVINYKELNKYTKFDGYFLPNKEVLINLVKNKTYYSKFDCKSGFWQIKMENDSIPYTAFSTPQGHYEWLVMPFGLKNAPQIFQSRMDKIFRNYKYIIVYVDDILIASETLEEHRHHLKEFANTCIREGIVLSSKKAVIEHKKIEFLGMILDKQGIKLQSHIGRKIIEFPDKLENKQQIQKFLGCLNYAEGFIKDLSKKRNTLQKLLRKNNTRGWNEEHTETVRSLKEECKNLPTLRLPDEKDRLVIQTDASDLYWGAILKTDINEICRYTSGTFNQAQINYPVHEKELLAIYKGIKKFSLFLLQKQFIVETDNSQVNSLINKILPNEPQYRRLHRLQVYLSYYNFKIVHIKGINNFLADFLSRNIEFGE

>lcl|ORF5

MNPEVNRIIGKIEAEVQRLKTEASDIIDAINYLKDASEAHEKGVNLLESYIDDLTKALSINTSPININPISIGSSSGPFREDKPIPDQVPNKTISIEVCSQEAGSPKSDPPKKFIEINNPYIEGDEKPLNPKEEEYKQFCTWVYSTCSHNNTIFRAPGRNMYPRMIVTKGADPLLVGKIAKYGYLDLIYPDIHLQEIASFDDLLIKEISRFAQKRPIYLKFYTISPEYYQNVEYQAFYMISIGHLTKNFKVEVGTEYTNVPHINEPWIRTRRARGIKAIWSIAKDLYKRNFRTIIKFQNYILITSEDSHTPSSLLLDFINEISTMRFPSSSKTMEQAYQLMEVADNIKKLPDKIRQTT

>HelAnn-006; NC_035449.2 chromosome17 (135624773-135617343rev); https://www.ncbi.nlm.nih.gov/nuccore/NC_035449.2?report=genbank

TCGGAAAATACCGCTCTGAATGGTATCAGAGCCACGCAGAATTCCTTCGCGGGGGCTCTAGATATTGATAGTACTTTGCATGAGATTGAGTTTGATAATTTAATTGTATTAAATTTAAACTTTATAAATAATAAAGTTATCATGCTTAGATTAAATCAACAAAATGATTTTTTAGAAAACGAAAATTTAGATTCTGAAGTTGATTTTGATGAAAATTATATCTCTGACGAAGATTTTAATTTTAGTAGATATGAATGGACTAGTTCTGACTTTGTATATGATGATACTGATTGGCAATATAATTCATCTTAATGGATAACCATAATTTTAATAAACTAAAAGAAAGTGAAAATTCTTTAAAAATAGATAAAGATTTGATAGAAGTACAAAAAACTATTATATTTTATAGAGAAAATATAATTTTTAGACTAGATAAAATTATGGAATTATTAGAGTCTACATATCCTAATAGGAAAGATAAAGAAAAAGTAAGTAGGCTACAAGATCCTTTGATCCAACTCTTACAAAATATGAAAATACCTAAGGGTTATAAAGAAATTAGAAATGAGTTCAGATACGGAAAGTGAAAATAATATAGTCCCTAACGTAGAAGATTCTGAGGTAGAAGATTTAGTAACCAAAGTAAGAGATATGGAGTTAAATGATTATAAATATTAAAGTTGGTGAAAATTTTAATATAAATGATACACCTAGTACAAGTAAGGATAAAAATATACCAATAAGAAATGAATCTCAAAGGCCTACTCTAGGATATGCTGTAATAAAGTCAACAATGAAAGAAGAGGATAATTATAAACCATACCAAGTTATGAAAAATGAACCAATTAATCCTTTTGGAATATATTTAGATCTAGATTGTGTAAACAATACTGAAGAAGCAATAGATAAATGGGAAACCGCTTTTAGAATAGAAGTTTCTGTTAATAAAATGGACATTGAAACAATAAAAGGATTTTTAGAAAGGACTCTGTTAAACAGTGCTTTAAGATATTGGCAAAACATAAGCCCTGAGGCAAAAAGCTATATATTTGAATCAGAAGATCAAAATATAGCAAACATAATAACAAGAGCTGTTGAAGCTTTTAGATTAGAGTTTTGTGGGGAAGGTAATATAATAAAAGACCCAGCAACTATACAAAAATATTTGACAGCTTTATTACGTCTACAATTATGTGATATTTGTGAAATTGATAGATATATTTGTGTTTTTCAAGATTATTATTTTCATATTTATAACCAAGTACCAGATACATGTAATTATTTACCCTTATTTTTTGCAAAGATACCTGACCCATGGGGGAAAAAACTAATAAATACATATAACCCAGGAACAACTGATACTTTAGGAAAAAGAATAACACATGCTAGAGATAAATTGAGCGAGTGGTGTGGAGATGCCATACTAGCTAAAATGAGTAAAAATTTAAAAAGAAAAATATCTTTATGTTGTAGTAGCCCAAAAATGCCTTTAATGATAGGATGTGATAGTTCTTTTTACTATGGAAAGATAAAAAGAAAATATAAGAAAAGAAGCCAATTTAGGAAAAGATATTTAAAAAAGCGTAAAACCAAATACTATAAAAGAAAAGGATATAAACAGACTTTTAAGCATACATATAAAAGAAAACTAAATCCCAACAAATGTAGATGCTTTATTTGTCATAAGGAAGGACATCTTGCAAATAATTGTCCTAAAAAGTTTAATAAAAATCTTAAAATATTTGAAATAGATGATGATATGGAAAAAATGATAGATGAAGGAGAATTCATACAAATTAACGATCTTCATGATATAGATTCTGATGAAAGTATATTTATTTTAACTGAAACTGAATATACTTCCTCTGAAAATGAATAAATCTGACATAGCTACTACTAGTAAAACAGATAATTTTATAATAGATTGTCAAGAAGAGGAACTAGGAGAATATAAACAAGAAATTTTAATAGATAAGAAAAAATTAAAACAAATACAACATGAAAATTTTTCATTATCAAATTATTTGGGATTTAAAATAAATACTTTACAAAGGTTATGGATAAAACCAAGAAATCATAATCTATATTATGGGTTAAAAATTCAGGAACGAGCTTTAGATATTAATATAACATCTAACAAAGTAATGATACCATTATTGTCTAAAAAAGATATTCAAAAAAACTACAAAAGATAAAGCCTGAAATAAGGAATACTTTAGGATGGGTACATGTTGGAGCTATCCAAATAATAATAAAATCCACCTTTAAAGAAGGTATAGATACCCCTATAGAATTAGCAGTTATGGATAATAGAATCCAAAACAGAGAAGAAGCATGTCTAGGAATACTACGAGGAAATTTGCAATATGGTAAACTAAAGTTTAATATATACCCAAGAATTTCCTATTATATCCAAGACAAAGATTTTGATAAAACTTTAAGTTTGTTACAAGATTTTAAAAGAAAAGATTTCTTTAAACAACAAAATAGGCCATATTCTATTACATATGCAATATCATATGCTATATCAAATACCCACCATTCTGATTGTTTTAGTATAAAAGATACTATAGATTTTCTTTTTTTGTTTAATGAAGTATGCCAAATACAAATACCAACCCTACCTAAAATTGAGGAAATAGATTCAAGACCATTAAGTCTTGATTTACAAGATAAACCACTTCTATTTACCAACCAGATCACCCCTAGATTATCCTTTACAAATAAAGGTGTAGTTAGTCATCCCCTTGCAAGGCTGACTAATTCTAGTAGATTTTATAATATCATTGATGCTAAAGAAGATAACATAAGAAATTATAAAATAAAAGGAGAATATTTCAATGGAAAGAAATTCTCTCATATAGATATACTAATAGATACAGGTGCAAATGGAAATTATATAAATCATAAACTATGTGACCATTTGCAAAAATATCCGTTAGAAGAACCTCACCAATATATTAACTTTAATGGTGAGTTACATGAAATAAATGAAGCCATAGAAACTATAATTAAATTTGGAGAAGAAAAAATTCCATTAAGATTATTAATAGGTAATGAAGATGAAAAGGATACATTAGAAATAATGCTAGGATTAACCTTTTTAGAAAATGTTAAACCTTATCAAATAACCTCTTATGGGTTAAAAATCACATATAATGATAAGATGATATATATACCAAAATGACAAGTCCTAAAAGTATATACATCCCTATAGGAATAACCTATAAAGATTATAAAGCTGAATACTTTGCTGCTTATATAGATAGTGGATCAGGGTTATGCATATGTAAACCTGATTGCTTTCCAATAGAATATCATGAAGATTTAACACCTTGTAACGGTGTTAGTTTTTCAAAAGATATAATCCCTCTGAGAAGAGGAGTAAAAAATCCAACAATATTAATAGGATCATATATGGTTAAATGTCCACCATTTTATTTTTATAATTCAGGATCAGATGTACTATTAGGAAATGATTTCCTAGAAAGATTTAACAAACTAACTTTTGATATAATAGCATATCAAATAATATTAAAAACACCATGTCACCGCTTAATAGTTGTAAAAAGAATAAAAAATGCATATGGGAGAAAATTCCCAATTAATTTTACAACACGCGCAAGCCAGCGTGGTGATTTAGGATACAAACAAAAACCCAAACTTGAAAAAGGTATACCAATTTTGGAATATTATCCAGAAGAACCATTAGATTACCAAGTAAATTTAAGAAACTTAGATAATCACATAGAAAATATAAAATATAAGTTAAGACAGTGTTATACAGATAATCCATCACAATTTTGGAATAAAAATCAAATAATGGCAAAATTAGAAATGAAAGACAAAGATAAAGAAATTAGAGTAAAGCCTATGAGATATAATCCAAAAGACCGGAAAGAATTTAGCTCCCAGATAAAAGAGCTCCTGGATTTAAAATTAATCAAAGCTAGTCATAGCCCTCATAGTTCTCCTGCATTCATGGTAAGAAAACATGCAGAAATTAAGAGAGGTAAGCGTAGGATGGTAATAAATTATAAAAAAATTAAATGATAATACTGTTTTTGATGGATATTTTTTACCTCATAAAGAAAGTCTAATAAATTGGACAAGAAATAAGAAGATATTTTCTAAGTTTGATTGTAAAAGTGGATTTTGGCAAATAAAAATGCATCCTGATAGTATTGGATACACAGCTTTTTCTACTCCTCAAGGGCAATATGAATGGCTTGTAATGCCTTTTGGATTAAAAAATGCACCACAAATATTCCAAAGGAAAATGGATATAATATTCAAAGATTATGATTTTATTTTTGTTTACATAGATGATATTTTAATTTTATCAGACAATGTTAATTTGCATCTAAAACATCTTGATATTTTTGCAGATTTATGTATAAAACTTGGATTAGCCTTATCTGAAAAGAAAACTAGTCTATTACAAGAGAAAATAGACTTTTTAGGGATGAAAATAGATGGAAAGGGAATAGAACTCCAAAGTCACATTCTTGAAAAAATTACAGCCTTTCCTAATAAGTTAGTAGATAAAAAACTGGTACAAAGTTTTCTTGGCATATTAAATTATGCTTCAGTTTTTATAAAAAATCTTGCAGAATTAAGAAAGCCCTTTCAAAATCTACTTAAAAAAGATAAAATATTTACTTTTGATAAAAATCTAGAAAACCAAGTTAAAAAGATTAAGGAATATTGTAAAAACCTACCAAAATTACCACTTCCTAAAGAAAATGATAATCTTATTTTAGAAACGGATGCCTCAGAATGCTATTGGAGTGGGTTACTTAAGAAAATAGATTATAATTCTGAAAATGAAAAAATAGGAGAAAGTATTTGTAGATATTGTTCAGGTACTTTTAGTGATACTGAAACAAGATATCATATAAATGAAAAAGAATTATTGGCTGTTATTAAAAGTTGTCAAAAGCTTTATTATTTTTTATTACCAAATAAATTTTTGCTAAGGACAGATAATACACAGGTAAAGGCTTTTATTAAAAATAACTTGCCTTCCAAACCAGAATATAAAAGACTTATTAGGTGGCAAACACTATTATCTGAATATCATTTTGATATAGAAATAATCAAATCTGATAAAAATGCTTTAGCAGATTTCTTAACCAGAGATGGAGGAGCAAGAATTAGCCCAAAGGATGGATAAATATATTGATGATATGGATAAACAAATAAAGTCGATAAAAGAAATTACAAAAGATATGGGAGAAATGCTAAACATCATTGAAACAATCCCCGAGAAGATCCCAGATATAAAGGATTTGATTGAAAGAAATTGTCTTCTCATAAATTCTAAGATATCATTTTATGAGTTTGACAGTATGAAGATAAAGCTTCTGGTAAAGCATCACATAAAAGAAAGAGTAGATCTTTCTTTCATTTATCCTGAAGTAAAAAAGGGAAAAAAGAGATAATCCTTCTACTTCATCAGTTGGAGTACAAACGATAGACCTTACTTCTGACCAGGAAGAACCTAAAGAAAAATCTTTTTCATTTTTCCCCAATACAGGGAAAAGCTCTACCTTTAAAAATATTTTTCAAGAATTGCCAACTCAATCAAATTATGTTGAGCAACAATGATTCAAGATGAAGGAACTATATGGCCTTCAAAATACAGATATAAGATATATTGATTTTACAGGGATCTTCCCTAGAATCAATTATCTACAAGGAAACTCTCCTGAAGAGATAAGATACTGGTATGATTTTGGAGTAGTCAATAAGATATATCTTACTTTACCCGATTTTCCCGAAATCTCCTTTTTACCAAAATGGGTCAAAGATGGAGTTAGAGATTGTTATTTAAATAATCCCACAATAACTCCAAAAGATGTCATGGCTCTAAAACTCTTTTCTGCAGGGCCTGATTTTTATAATGAATTTTATTATCCAGCTTTTCACTTTATTCAGATAGAAAAGGTTAATTTAGCCTTTACTTATTCAGTTAATACTGAAAGAAAGATTTTTCATAACTTTAATGTAAATGATGTTCATTACAGAAGAGGATTAGGAATTAAAGTAATACTCCAAGCATTGCAAGCTGCTTTTAAAAATGGATTTAGAACATATGGTGGGAAAAATATGTTTTCTTCTGTCATGATTTCTCCAGCAAAAAATACTCTACCATCAGCCAAAAAACATTTAGAAGATAAAATCCAATTTTTAGAAAAAGGCATAATAAGATCGAGTCCTCAGGCTCAAGAAAGGGTTTGCATATTTAGAGATCATAAAAAAGGTATGAATCCAAGAGATATGTGTCCAATATGCTATAAACAAAAAGACAAGGACACTATGAGTATTGATTCTCAAAATGAGGGTCAAACATCTGATAATTAAGGATTTTATAAATCCTTTACTCCAAAAATGATTTTCAAATCATCAGATTTTACCGTTCAAAGATATAGCATACAAACAAGGATTTAAGACTCACATAGAGTCAAAATTTGCTTTTCCCATGCCCACCTACTTGAGAATGCAAGGATGAACGTGGCTAAAGAGGAAATAATGGATAAATAAGCTCAAGTTCCCAAGAGTAGACTTTCTTTTACAAAAAAACAACTTCCAAGGCTTTATGACAAAAGCTAAAGTTACAATAAGGCTCATGTCTTAAGGAAGCATGTGAAAAGAAAAGGACAACGACCTGTAGCTTATCTAAAGAAGGAAGTTATAAAAAGAAGGAAGAAGACTGATGGGAGGCATCAAGCTTCAACGAGTGTTTAAAAAAGTTTTTATGTACTAAAAAAGTTTGTTATATTTTTGCTTAAGTTTTGTATCTTCTCTAAACATCTTCCATTGTAAATCATCCACCATGAAATAAAAGCTTTCCCTTTTCCTTATAAAGTCTTTTTGAGTGTTCTTTTTGTTTTTAGATATTTCTCTATTAAAAAGAAAAAATCTCCAATCTCAAACCGTTTCTTTTTACCCTACACCGGAGGTAGGAAACGAAACCAGGTTGTGCTGGATATTGCTGAAGGATCCATAGGGGAGTTACCTAAGGCCTCTTAGGTTTGGTGTGAAAGAACCTAGTAAAAAGACCCGTGGTGTAACCTGGCAATATTTTGGTCAAAACGGAAAAAAGGTATGGAAGCATGTTGGACCCTTGTTCTTTTTAATTTTGAAATATTTTAAAAATAATTGACACTCTTAGGTTTTATAAGGAAACTTTTAACAAACAAATTCAAACAAAGTTTTATTTTATGATTGTTTTTATAATCTAGAAAAAAAATGTTACTTTTTAATTTTAAAGTTTTTCTCAAAAATTTTTGTCATATAAAATAGATTTAATATGTGGTTAACTTTAATAATAAACATAAACAATTTTGATATAATCTTTAATCTTGTAGATAATAATTTACTTGAGTTATCAATTTTGAACCGACAAAATATTTCGGAAAATACCGC

zf-CCHC Zinc knuckle (coat protein) e-value 0.018

MP Movement protein e-value 3.8e-15

Gag-asp-protease Aspartyl protease e-value 0.00021

Peptidase_A3 Cauliflower mosaic virus peptidase (A3) e-value 2.3e-10

RVT_1 Reverse transcriptase e-value 2.5e-19

RT-RNAseH RNAseH e-value 2.1e-20

>ORF1

MIINIKVGENFNINDTPSTSKDKNIPIRNESQRPTLGYAVIKSTMKEEDNYKPYQVMKNEPINPFGIYLDLDCVNNTEEAIDKWETAFRIEVSVNKMDIETIKGFLERTLLNSALRYWQNISPEAKSYIFESEDQNIANIITRAVEAFRLEFCGEGNIIKDPATIQKYLTALLRLQLCDICEIDRYICVFQDYYFHIYNQVPDTCNYLPLFFAKIPDPWGKKLINTYNPGTTDTLGKRITHARDKLSEWCGDAILAKMSKNLKRKISLCCSSPKMPLMIGCDSSFYYGKIKRKYKKRSQFRKRYLKKRKTKYYKRKGYKQTFKHTYKRKLNPNKCRCFICHKEGHLANNCPKKFNKNLKIFEIDDDMEKMIDEGEFIQINDLHDIDSDESIFILTETEYTSSENE

>ORF2

MDNRIQNREEACLGILRGNLQYGKLKFNIYPRISYYIQDKDFDKTLSLLQDFKRKDFFKQQNRPYSITYAISYAISNTHHSDCFSIKDTIDFLFLFNEVCQIQIPTLPKIEEIDSRPLSLDLQDKPLLFTNQITPRLSFTNKGVVSHPLARLTNSSRFYNIIDAKEDNIRNYKIKGEYFNGKKFSHIDILIDTGANGNYINHKLCDHLQKYPLEEPHQYINFNGELHEINEAIETIIKFGEEKIPLRLLIGNEDEKDTLEIMLGLTFLENVKPYQITSYGLKITYNDKMIYIPK

>ORF3

MTSPKSIYIPIGITYKDYKAEYFAAYIDSGSGLCICKPDCFPIEYHEDLTPCNGVSFSKDIIPLRRGVKNPTILIGSYMVKCPPFYFYNSGSDVLLGNDFLERFNKLTFDIIAYQIILKTPCHRLIVVKRIKNAYGRKFPINFTTRASQRGDLGYKQKPKLEKGIPILEYYPEEPLDYQVNLRNLDNHIENIKYKLRQCYTDNPSQFWNKNQIMAKLEMKDKDKEIRVKPMRYNPKDRKEFSSQIKELLDLKLIKASHSPHSSPAFMVRKHAEIKRGKRRMVINYKKIK

>ORF4

MHPDSIGYTAFSTPQGQYEWLVMPFGLKNAPQIFQRKMDIIFKDYDFIFVYIDDILILSDNVNLHLKHLDIFADLCIKLGLALSEKKTSLLQEKIDFLGMKIDGKGIELQSHILEKITAFPNKLVDKKLVQSFLGILNYASVFIKNLAELRKPFQNLLKKDKIFTFDKNLENQVKKIKEYCKNLPKLPLPKENDNLILETDASECYWSGLLKKIDYNSENEKIGESICRYCSGTFSDTETRYHINEKELLAVIKSCQKLYYFLLPNKFLLRTDNTQVKAFIKNNLPSKPEYKRLIRWQTLLSEYHFDIEIIKSDKNALADFLTRDGGARISPKDG

>ORF5

MKELYGLQNTDIRYIDFTGIFPRINYLQGNSPEEIRYWYDFGVVNKIYLTLPDFPEISFLPKWVKDGVRDCYLNNPTITPKDVMALKLFSAGPDFYNEFYYPAFHFIQIEKVNLAFTYSVNTERKIFHNFNVNDVHYRRGLGIKVILQALQAAFKNGFRTYGGKNMFSSVMISPAKNTLPSAKKHLEDKIQFLEKGIIRSSPQAQERVCIFRDHKKGMNPRDMCPICYKQKDKDTMSIDSQNEGQTSDN

>AtaBux-077; *Atalantia buxifolia*; Scaffold27195 (1635419-1635778rev); https://www.citrusgenomedb.org/;

GGATAACGTTATGTTACAATAAATGTAACATACATTTAGGAGGAAAATATCACAGAAACCCCTAAAATTTATGAAAACTACATTAATAGAATTAAAATAACTACTTTTAAATTTAAAAGAAGAAATAAAAGATATAAGGCAAGAAATAGGAAAAATCAAAATAGAATTATCAGCAATTCAACTAAGCAGAAAGAAAAATAAAAAATTAGAAAAATTTAATATAATAAAAACGGGAATAATTGCAACAGAATTCAAAAGACCTATACAAAAATCCCAAGTAGGAAGTTATGATGAATGGTTCATGAAAACCCAATATCCTAAAATATTAGAAGAAGCAGAAAAATTAAAAGCAGAAATGAAGAGTAAAGAAAAAGGTAAAGAAAAAATAGAAATTAAAGAAGAAGTTCTCAGTGAGTCAGATTAATGGAAATTGAATTAGAAGATTTAACTGAAAAGTTTAGTGATATAAATTTAAGTGATTTAGAAGATTTTGAAAAAGAATATCAAATAAAAATGAATACAGCTGGAGGTCCAGAACTAAAACCTAATATACCTGAACCTAGTCATAGATATGATAATAAAAGAAAAAAAAAACCATTTTTTGAATATTACCCACCAATCAAAAATACTCCTTGGAAAAAAGAATATCAACCTAATAATGAGAAACTAGTACCTGTAAATAATACAGGAACATTTTTAGATTTAGATTGTAAAATAGACCCTTGAAAAACTTTAGTTGAATGGGGAATGCAAATGAGATTATTTTTCACATATAATAGTTCAAATGATGATTGGAATGTAGAAACAGAAGAAGAAAAATTAAATATATTTGTTGAAATCTTAATAGCCAGTTTAACAGGAGATGTATTTAACTGGTGGAAAGGATTAAATGAAGAAACACAAAGATTAATAAAAGATTCAACAAAAATAGCTTTTACAAGAAGCAAAGCATTAGGAATCGAAAGAATAATTGAATATATTGCTAATGAATTTCTAGGAGAAGATTGGTTGGAAAACAGTGCAGCAGAGGTAAAACATGACAAGTTAGAAGCCAGAATGAAATTAATAAATTTGTCAATTTGTAATATGTGTTATGTAAAAGAATACACTTGTGAATTCAAAAAATATTATTATACACAATTTTTAAGTAATGAAGATCAAAATGTTTATAAAAATTTATATTATTCTAAATTACCTTATCCTTGGAATGGATACTTTATAAATGAATATGAGAGAAACAATAATATAAATAGAAATCCTGATACGTTAGGATCCAGAATAAGATTTTTAAATACAAGATTAAGTGAAATTTGTATTCAGAGAAGTTTAATAAAGAAAAGTAAAAATATAACAGAAATATGTTGTGAAAAAACAGAAATGCCAACCCAATGGGGTTGTTATAATCCTTATAAAAGAAAAAAGAGATTTCATAAAAAAGAAAAAAGATATAAAAAGTATAAAAAATATAATAATTTTAAAAAGAAATATAGAAAGCCTGGAAGAAAATATTACAAAAGAAAATATAAGAATCAAAAGGGAAAATTAGATAAATCCAAATGCAAATGCTGGAATTGTGGAGAAATAGGACATATAAGCCCAGACTGTACAAAGAAAAAAGTTAAACTTTTAAAAGAAGATTACGAAATCATTAAAAATGATTTAGAAGAAATAAGTGATCTATCAGATCATGAAGGAAATGAAGTTTATGCAATAATAGAATCAGAATCTGATAATGAATCCTAAAAAAGAAAAAGAGGAAGCCGATCTAGAAATAGAAGAAATTGAAGAAATAGAAAAAGAAGAAAATAAAACCGGATATAGTCTAGTAGCCACTTTTAATAAAGAAAAATATAATAAAATTTTAAAAAAGAATATGAAAAAAACTGAATATGGAATAATACATAAACTCGGAGATATATTCAAAAGAAAACAAATAGTATTGCATGAATACTATGAACAAGAAAAAGCAGTAACAATCACACAAGCTGAAGGAAATATTAGATTTAGTTTAATAAATAAAAGAACTATAGATTTAGCCTTGAGAAAAGTAAAAAATGAATCAATAAGAGATAAAATTCAATATGTATATTTAGCAGAAATACAGATATTAGTAAAATCTCTTTTTAAAGAAGGTATAGATAGTCCAATAATCTTATCTTTACATGATCAAAGATTTAATGATGCTAGCATAGGACATTTAGGAACTATTGAAGGAAATTTGTGTTATACTAAACTACTATATACTTGTCATCCTAGATATTGTGTACACATTAAAGATAAAAGTATAGATGAAACATTAAGCCTACATTTTAAATTATTAAAAAAGAACTTAATGAAAGAAGGAAATAGATTAATGACAATATATTATTCAGCATTATACAGTTTCTGTAATTCAAATTATGGAGAAATATATGGAAATAAACCTTATATAGAAATAAATAAAGAATGTGAAGAGATAGCAACACTTATAGAACCTACGATCCAAGATTATAGTATACCAATAGAATATAACCTTAATGTAGATGATAAGAAACAATTTATAAATACTGATAATGAGAATTCTATAATCCCCATAAATTTCGCAGGAAGAACTTTAATAAGAGGAAGTTCATCTAGAAGATTAAGTACTGATAGACAAATAATTAAATCATTACCAACTAATATAAAAATATTAGCACAATATTTTGATGGGATAAATTATACAAATAAAATACCTATATCAATAGATACAGGAACAAGCCATAATTATATGAACTATCTTGAAAATAAAGGACTTATTATTAAAGAAAAAGAAACTCCATATGAATATATTGATTTTAATGGAAATAAACATATATGTAAACAAGAAGTCAAAGTTCCAATAATAATAGAAGGATTAAGAATCATAGTACCTTGCTACATAGATTCCTCTATGAATACTGATTTAGACAAACATATTATATTAGGCAATAGCTTTTTAAATGATTTAGATTATTATAAAATAGAAAAAGATAAAATAACTTTACAAATTGAAGGACAAAAAATAATATGTGAGACATGTTAAATCAAGCTTATATAAAATATCCTATGTCTGTTTATATACAAATAAATATTCCAGAACACGATATAGATACATGTGCTCTTATTGATACAGGAAGTGAAATTACTATTATGAAATCATTTTTATCAAACCAGTGGAAACAAAATGGAAGCTTAAAAATTATTGGAATCACAGGAGATAAACAACAAGTTACACATTCATTAGTAAATTATGAAATACTTTTAGGAAGTAAACTAATTCGTATTAATCAAATTTTCCAATATAATAACATGGATTGTGATATACTCTTAGGCAATGATTTCTTACAACAATTTCAATATTATCAACAAACCACTTATATGATCACCCTTAAAACTCCATGTAACCATATACTTCGTGTTCCTCGTGAATTTAAACCCTACAGAGTTCAGCCAACCCAGCGTGGTGAAGTCATCAGTTATGAAAAACATTATTTAATACAAAAAGGACAGAGTTATAATATAAAATTAGAAACAATAAAATCACAACTTGAACAAGTTTATAAAGAAAATCCTTTAGCATTGTGGAAACCTGAACATCCTAGAGCAAAAATAGAGTTAATTACAGAAAAAATTATAAGACTTAAACCTATGATGTATACAGAAGAAGATAAAAATGAATTTAAAATTCAGATAAAAGAATTATTAAATTTGAGGTTAATAAGATATAGTAATAGTCCACATAATAGTCCAGCTTTTATGGTAAGAAAAAGATCAGAACAAATAAGAGGTAAAGAAAGAATGGTAATAAATTATAAAGAATTAAATAAATATACAAAATTTGATGGATATTATCTCCCAAATAAAGAGGTATTGATAAATCTTGTAAAAAATAAAACATATTATTCTAAATTTGACTGTAAATCAGGATTTTGGCAAATAAAAATGGAAGAAAATAGCATCTCTTATACAGCTTTTAGTACTCCGCAAGGACATTATGAATGGCTAGTAATGCCTTTTGGATTAAAAAATGCCCCACAAATTTTTCAAAGTAGAATGGATAAAATATTCAAAGATTACAATTATATTATAGTATATGTTGATGATATATTAATAGCCTCAAAAACATTAGAAGAACATAGAAAACATTTAAAAGAATTTGTTAATTTATGTATAAAAGAAGGAATAATACTTTCAGAAAGAAAGACAATTATAGAACATAAGAAGATTGAATTTCTAGGAATGATTTTAGATAAAAAAGGGATAAGACTACAAACACATATAGGAAAAAAGATATTGAAATTTCCAGATAAATTAGATAATAAACAACAAATACAAAATTTTTTAGGATGTTTAAATTATGTAGAAGGATTCATAAAAGATTTAGCTAAAAAGAGAAATACTCTTCAAAAATTACTTAGAAAAAATAATACTAGGGGATGGAGTGAAGAACATACAGATGCTATTAGAAACCTTAAAGAAGAATGTAAAAATCTCCCAGCATTAAGACTACCAGATGAAAAAGATAAGTTAATAATTCAAACAGATGCTTCTGATTTATATTGGGGAGCAATACTAAAAACTGACATAAATGAAATCTGTAGATATACTAGTGGAACATTTAATCAAGCACAAGTAAATTATCCAACTCATGAAAAAGAACTCCTTGCTATATACAAAGGAATAAAAAAGTTTTCTTTATTTTTACTTCAAAAACAATTCATTGTAGAAACTGATAACAGCCAGGTAAGCTCACTTATAAATAGAATATTACCAAATGAACCTCAGTATAGAAGATTACATAGATGACAAGCATATTTATCTTATTACAATTTTAAAATTATACATATCAAAGGAACTAACAATTTCCTTGCAGATTTCCTAAGCAGGAATATAGAATTTGGAAAATGAATACAGAGACAAATAAAATCCTCTCTGTCATAAACACGGAAATACAAAGACTCAAGGCCGAGGCATCAGACATGATCGATACAATAAACTATTTGAAAGAAGCTTCAGAATCACATGAAAAAGGTATACATTTGTTAGAATCATATATAAACGATCTAACAAAAGGGTTTTCAATAAACACTAGCCCAATAAATATAGGAAGCAGTAGTGGACCATTCCCACAACAGGAACCCGCTTCTGATTCTACTTCAAGTCGTACTATATCAATAGAAGTTGATCCAGTAAAAATATCACATGAGTCACCAAAAGCATCCTCCTCAAAAAAGTTTATCAAAATAAACACTACACTCGAAGAAGAAGAAAAACCAATTCCTTTAAATCCAAAAGAAGAAGAATATAAACAATTTTCTACTTGGATCTATTCTACCTGTTCTTACAATAATACCATATTTAAAGCACCAGGGAAAAATATGTACCCTAGAATGATAGCCGTAAAAAGAGCAGATCCACTCCTCATAGGTAAAATAGCTAAATATGGATTTTTAGATTTAATATATCCTGATATAGACTTAAAAGAAATAGCTTGTTTTGATGAATTACTAGTCAAAGAAATTTCTAGATTCTCCCAGAAAAAATCTATATATTTAAAGTTTTATACCATCAGTCCAGAATACGACCAGAATGTAGAATATCAAGCCTTTTATATGATCTCTATAGGTCATTTGTCAAAAAATTTCCAAATAGAAGTAGGGACTGAATATACTAATGTTCCTCAAATAACCAAGTCATGGATTAAAACCCGTAGAGCCAGAGGAATAAAAGCAATTTGGAGTATAGCTAAAGACCTTTATAAAAGAAATTTTAGAACCATAATCCGTTTTCAAAATTATATCCTTGTCACTTCTGAAGATAGTCATACACCTTCAAATATACTCTTGGAATTTATAAATGAAATTAGTACATTAAGATTCCCAAGCAGCAGTGAGACACTACAACAAGCTTATCAGCTAATGGACTTAAAGGCACCTGACAAGTGAAGCATGTGACAAACAAAAAAGATGAAGGTAGCAGATAAAAAATAGACAGCTACAGAATAATGGCCGACAAAAGCAAAGCGCACTCAGAAAAGAAGATAAAGCTCGTGACAACAAAGTGAAGAAGACAAAAGAAGAAAAGAAAGCATATGCCAACAGACATAATGGCAGACAAAAGAAAAAGTCTTGTAATTATTTTTGCTTTTAAGTCTTATAATCATTTTTGCTTTTGTAATTTTGTAGTTTTGTAATAATTTCGAATCGGGCGATGTAAAGAGAAAGAAGTAAATAAAAAGAGGAGAAAGACAGAAGTCTTCTCAGACGCAGATTTTACAACAATAATTCATTCTCTCTGAAATCAACTTACTCTGAAAACCAAAAAACTCTCGAAAAACATCCATCTTTCCTTAGAAAGCAAATCAAAAATAAAGATCATCCAAAAAATAAAAAGATGACGAACTAAACACCTTCTTGCTCAAATCCAAAAACCAAAACACCGTTAATCAAAAACAAAAGAACCAGAGAAGAGAAAGAGAGACCTAAGAATTAATCAACCCTTTTGCAGGAAGATCTAAGCTCCATACAGTAAAGCCGTGCTGGTCCGCAGATCCCGTTTAGGCAGAAGACCGTTGAGGGAGTTTGGACAATTAGGCTGCAGGTCACTGGAGAACAGGTCAGAAAGCAAATGAGAAGATATATTTTTGGGTAATAATTCTCAAACTTTATCTCTTGGGGGAGTTGTTGATGATTGATTGGTGAAGTGGTTTGTGGAGGATGAGCAAACTATTAAAAATGTTAACAGACGTTAAAATTTTATTATGAATAATCAAGTTTATTTAGGAGAATCTAAAGAATATATAATAGATCCGACAACAAAAATAACATATAATGATTATCATATGATAATAGATTTTAATATAGGATCTTTTAAACAAAGTTCTTATTTGTTTAATAAACAAGAATTAGTCAAAAGAGAAGACCTTTTGGCGGTATACTCGTTAGAATTACCTAAAGAATTAAAACGAAAAATAATAAATTATATAGAAATAGATAATTTAAAACATAATATAATCATGTTAACAATAGAAGAATGGATTGATATAATGAAAGTATATATAACTATTGAACATACAGGAGAATCTTCATATACTCATACTTATAACTCAGATAATGAAGAATTTGATATATAATTTGAATATAAAGGAATATAGGATAGAAACCAAGACATGCAGACTATTGGTCCAAAGGACAATTAAAGATCTTGACTGAATTGTCAAGGATTTCATTGTCCTGGCCTAACAATTCCATGTACCTTATATTCATAATAAATTAATAGAAACTTTATATGGATTTTGTAGTTATTCTAAGATTAAAAATAAATGTTAATAAAGTGGACATAATTTTAATTATTTGTATTTTAATTCATTTAATTTGTATATTAATTTATTTGAAATATTATTAGATCTTATAAGATCTTGTTAATAGTTTTGAATATTTGATTTTGATAATCTATTTTATAATTTTGTTAATATTTGTTATATTTTGTTTGTTAATTTTGTTAATTTGTTAATATTTGTTAAATTTGTTAATATTTATTGCCCCCGGTAAACAATGGTATCAGAGCCATAGATCTAATGGTTTATAACGAATAAAAATATAAAATGAATTTCGGATAAAAGTTTATACTATAAAGATAATAAATGGCAGATCCAATACAATCAATAGTTAATAGTCTATATTTATTAAAGGTATTTAGTAAGGAAGAAATAAAAAATTTGATAAAAGCAATAAAAACAGATAAAAATAACATAAGAAATATAGATTTAGAATTAACAGAAGAATTAAGAGAAAGAAATATGGATACATTAGGTGCAGAAATTATAATAGAAAAGAAAATGAAATTTATAGATGAATTTGTAAAATTTTTAATTTATTGGTATAAAAATGAGACATTATGAATCAAGCCTTTAATATAATACATCAATTGTCAGACTGCATAGATAACATGTGGTTAAGATGGGAAAATATAATTATTACATATATAAACCTAATCAATCAAAAAATACATATATTAGAACAAGAAAGAACTGATTACATAAATGCAGGACCAGAAATCTATTCCGAATTTGTAGATTTATATGAGTTTGATAATCATTTACTAATGTTTAAAGAGATAAAGAAAACATTAGAAAAAATAAAAAATAGATGTAGAAGGGATGGAAGAAGACAAATAAATCCACAACAATAAAAAGCAAAGTTAGAGGATTCAAATGAAATTTGCCAAGTAGAATAAGACAAGGAACGTAAGATACATCAAGTGTAA

zf-CCHC Zinc finger (coat protein) e-value 5.5e-06

MP Movement protein e-value 1.1e-27

Gag-asp-protease Aspartyl protease e-value 0.12

Gag-asp-protease Aspartyl protease e-value 0.028

RVT_1 Reverse transcriptase e-value 1.1e-28

RT-RNAseH_2 RNAseH e-value 4.7e-24

>ORF1

MQMRLFFTYNSSNDDWNVETEEEKLNIFVEILIASLTGDVFNWWKGLNEETQRLIKDSTKIAFTRSKALGIERIIEYIANEFLGEDWLENSAAEVKHDKLEARMKLINLSICNMCYVKEYTCEFKKYYYTQFLSNEDQNVYKNLYYSKLPYPWNGYFINEYERNNNINRNPDTLGSRIRFLNTRLSEICIQRSLIKKSKNITEICCEKTEMPTQWGCYNPYKRKKRFHKKEKRYKKYKKYNNFKKKYRKPGRKYYKRKYKNQKGKLDKSKCKCWNCGEIGHISPDCTKKKVKLLKEDYEIIKNDLEEISDLSDHEGNEVYAIIESESDNES

>ORF2

MNPKKEKEEADLEIEEIEEIEKEENKTGYSLVATFNKEKYNKILKKNMKKTEYGIIHKLGDIFKRKQIVLHEYYEQEKAVTITQAEGNIRFSLINKRTIDLALRKVKNESIRDKIQYVYLAEIQILVKSLFKEGIDSPIILSLHDQRFNDASIGHLGTIEGNLCYTKLLYTCHPRYCVHIKDKSIDETLSLHFKLLKKNLMKEGNRLMTIYYSALYSFCNSNYGEIYGNKPYIEINKECEEIATLIEPTIQDYSIPIEYNLNVDDKKQFINTDNENSIIPINFAGRTLIRGSSSRRLSTDRQIIKSLPTNIKILAQYFDGINYTNKIPISIDTGTSHNYMNYLENKGLIIKEKETPYEYIDFNGNKHICKQEVKVPIIIEGLRIIVPCYIDSSMNTDLDKHIILGNSFLNDLDYYKIEKDKITLQIEGQKIICETC

>ORF3

MLNQAYIKYPMSVYIQINIPEHDIDTCALIDTGSEITIMKSFLSNQWKQNGSLKIIGITGDKQQVTHSLVNYEILLGSKLIRINQIFQYNNMDCDILLGNDFLQQFQYYQQTTYMITLKTPCNHILRVPREFKPYRVQPTQRGEVISYEKHYLIQKGQSYNIKLETIKSQLEQVYKENPLALWKPEHPRAKIELITEKIIRLKPMMYTEEDKNEFKIQIKELLNLRLIRYSNSPHNSPAFMVRKRSEQIRGKERMVINYKELNKYTKFDGYYLPNKEVLINLVKNKTYYSKFDCKSGFWQIKMEENSISYTAFSTPQGHYEWLVMPFGLKNAPQIFQSRMDKIFKDYNYIIVYVDDILIASKTLEEHRKHLKEFVNLCIKEGIILSERKTIIEHKKIEFLGMILDKKGIRLQTHIGKKILKFPDKLDNKQQIQNFLGCLNYVEGFIKDLAKKRNTLQKLLRKNNTRGWSEEHTDAIRNLKEECKNLPALRLPDEKDKLIIQTDASDLYWGAILKTDINEICRYTSGTFNQAQVNYPTHEKELLAIYKGIKKFSLFLLQKQFIVETDNSQVSSLINRILPNEPQYRRLHR

>ORF4

MNTETNKILSVINTEIQRLKAEASDMIDTINYLKEASESHEKGIHLLESYINDLTKGFSINTSPINIGSSSGPFPQQEPASDSTSSRTISIEVDPVKISHESPKASSSKKFIKINTTLEEEEKPIPLNPKEEEYKQFSTWIYSTCSYNNTIFKAPGKNMYPRMIAVKRADPLLIGKIAKYGFLDLIYPDIDLKEIACFDELLVKEISRFSQKKSIYLKFYTISPEYDQNVEYQAFYMISIGHLSKNFQIEVGTEYTNVPQITKSWIKTRRARGIKAIWSIAKDLYKRNFRTIIRFQNYILVTSEDSHTPSNILLEFINEISTLRFPSSSETLQQAYQLMDLKAPDK
